# DEAD‐Box Helicase 17 exacerbates non‐alcoholic steatohepatitis via transcriptional repression of cyp2c29, inducing hepatic lipid metabolism disorder and eliciting the activation of M1 macrophages

**DOI:** 10.1002/ctm2.1529

**Published:** 2024-02-01

**Authors:** Deng Ning, Jie Jin, Yuanyuan Fang, Pengcheng Du, Chaoyi Yuan, Jin Chen, Qibo Huang, Kun Cheng, Jie Mo, Lei Xu, Hui Guo, Mia Jiming Yang, Xiaoping Chen, Huifang Liang, Bixiang Zhang, Wanguang Zhang

**Affiliations:** ^1^ Department of Hepatic Surgery Center Tongji Hospital Tongji Medical College Huazhong University of Science and Technology Wuhan China; ^2^ Hubei Key Laboratory of Hepato‐Pancreato‐Biliary Diseases Wuhan China; ^3^ Department of Hepatobiliary Surgery Union Hospital Tongji Medical College Huazhong University of Science and Technology Wuhan China; ^4^ Department of Neurology Tongji Hospital Tongji Medical College Huazhong University of Science and Technology Wuhan China; ^5^ Institute of Organ Transplantation Tongji Hospital, Tongji Medical College, Huazhong University of Science and Technology Wuhan China; ^6^ Institute for Management in Medicine and Health Sciences University of Bayreuth Bayreuth Germany; ^7^ Key Laboratory of Organ Transplantation Ministry of Education and Ministry of Health Wuhan China

**Keywords:** 14,15‐EET, CTCF, Cyp2c29, DDX17, DDX5, non‐alcoholic steatohepatitis

## Abstract

**Objective:**

Our study was to elucidate the role of RNA helicase DEAD‐Box Helicase 17 (*DDX17*) in NAFLD and to explore its underlying mechanisms.

**Methods:**

We created hepatocyte‐specific *Ddx17*‐deficient mice aim to investigate the impact of *Ddx17* on NAFLD induced by a high‐fat diet (HFD) as well as methionine and choline‐deficient l‐amino acid diet (MCD) in adult male mice. RNA‐seq and lipidomic analyses were conducted to depict the metabolic landscape, and CUT&Tag combined with chromatin immunoprecipitation (ChIP) and luciferase reporter assays were conducted.

**Results:**

In this work, we observed a notable increase in *DDX17* expression in the livers of patients with NASH and in murine models of NASH induced by HFD or MCD. After introducing lentiviruses into hepatocyte L02 for *DDX17* knockdown or overexpression, we found that lipid accumulation induced by palmitic acid/oleic acid (PAOA) in L02 cells was noticeably weakened by *DDX17* knockdown but augmented by *DDX17* overexpression. Furthermore, hepatocyte‐specific *DDX17* knockout significantly alleviated hepatic steatosis, inflammatory response and fibrosis in mice after the administration of MCD and HFD. Mechanistically, our analysis of RNA‐seq and CUT&Tag results combined with ChIP and luciferase reporter assays indicated that *DDX17* transcriptionally represses *Cyp2c29* gene expression by cooperating with CCCTC binding factor (*CTCF*) and DEAD‐Box Helicase 5 (*DDX5*). Using absolute quantitative lipidomics analysis, we identified a hepatocyte‐specific *DDX17* deficiency that decreased lipid accumulation and altered lipid composition in the livers of mice after MCD administration. Based on the RNA‐seq analysis, our findings suggest that *DDX17* could potentially have an impact on the modulation of lipid metabolism and the activation of M1 macrophages in murine NASH models.

**Conclusion:**

These results imply that *DDX17* is involved in NASH development by promoting lipid accumulation in hepatocytes, inducing the activation of M1 macrophages, subsequent inflammatory responses and fibrosis through the transcriptional repression of *Cyp2c29* in mice. Therefore, *DDX17* holds promise as a potential drug target for the treatment of NASH.

## Graphical abstract

- *DDX17* expression is elevated in the livers of patients with NASH.
- *DDX17* accelerates NASH development by promoting lipid accumulation in hepatocytes.
- *DDX17* alters lipid composition in murine NASH model.
- Hepatocyte *DDX17* induces the activation of M1 macrophages and subsequent inflammatory response and fibrosis through the transcriptional repression of *Cyp2c29* in mice.
- *Cyp2c29* expression is decreased in the liver of NASH models and meditates the function of *DDX17*.

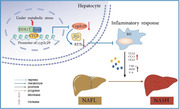

## 1 INTRODUCTION

Non‐alcoholic fatty liver disease (NAFLD) has become one of the most common causes of chronic liver disorder globally, with an estimated incidence rate 20−30% in the global population.^1^, ^2^ NAFLD covers a broad range of diseases, spanning from steatosis to non‐alcoholic steatohepatitis (NASH), liver fibrosis and hepatocellular carcinoma (HCC). Due to various risk factors, including genetic mutations, metabolic disorders and chemical injury, NAFLD is characterised as a heterogeneous disease with different mechanisms and progression. Although many studies have been conducted to assist in early diagnosis and to reveal specific mechanisms and therapeutic methods, effective pharmaceuticals are still lacking.^3^ Thus, further studies on its pathogenesis, mechanisms, animal models and specific treatments are required to provide molecular targets and treatment strategies for clinical use.^4^

DEAD‐box Helicase 17 (*DDX17*), also named *p72*, RNA helicase and its paralog, *DDX5*, also named *p68*, belong to the DEAD‐box family and function in most steps of gene expression.^5^ Except as RNA helicases, they also play essential roles in alternative splicing (AS) and transcriptional regulation. It has also been reported that *DDX17* and *DDX5* promote the invasiveness of tumour through the regulation of AS in several DNA‐ and chromatin‐binding factors, such as macroH2A1 histones.^6^ *RELA* recruits *DDX17* by activating the NF‐κB signalling pathway.^7^ Recently, *DDX17* has been reported to contribute to inflammation as well as tumour initiation and progression by regulating AS.^8^, ^9^ Furthermore, *DDX17* and *DDX5* dynamically orchestrate splicing and transcription by cooperating with several key factors, such as heterogeneous nuclear ribonucleoprotein (hnRNAP) H/F splicing factors^10^ and oestrogen and androgen receptors (ER and AR).^11^ Studies have shown that *DDX17* and *DDX5* play critical roles in transcription with a series of classic transcription factors (TF), such as ERα^12^ and nuclear factor of activated T Cells 5 (*NFAT5*).^13^ They have also been demonstrated to have significant functions in transcription, acting either as coactivators or corepressors by interacting with essential elements of the transcriptional apparatus like RNA polymerase II, histone deacetylases and CREB‐binding protein (CBP)/p300.^5^ Interestingly, *DDX17* also assists RE1‐silencing TF (REST) in transcriptional repression activity by promoting its interaction with the promoter of a portion of REST‐target genes.^14^ In addition, several recent studies have shown that *DDX17* promotes HCC and metastasis.^8^, ^15^, ^16^ However, few studies have explored the function of *DDX17* in cell metabolism and related diseases, with the exception of one study that demonstrated *DDX17*‐mediated transcriptional regulation of macrophage cholesterol efflux during atherogenesis.^17^ We previously found that *DDX17* exhibited an increased expression in the liver or HCC tissues of diethylnitrosamine (DEN)‐treated C57 mice fed‐with high‐fat diet (HFD) for 8 months compared with those treated with DEN and fed with normal diet (ND).^18^ Thus, we hypothesised that *DDX17* is involved in the progression of NASH and NASH‐HCC; nonetheless, the specific mechanism of *DDX17* in lipid metabolism and NASH remain to be thoroughly clarified.

This study highlights a notable increase in DDX17 expression in the livers of individuals diagnosed with NASH and in murine NASH models. We found that, mechanistically, depleting *DDX17* in hepatocytes alleviates hepatic steatosis, inflammation and fibrosis in mouse models of NASH induced by both HFD and methionine and choline‐deficient l‐amino acid diet (MCD), while the overexpression of DDX17 in hepatocytes markedly promoted lipid accumulation. Our analysis of RNA‐seq and CUT&Tag, combined with chromatin immunoprecipitation (ChIP) and luciferase reporter assays indicated that *DDX17* transcriptionally repressed *Cyp2c29* gene expression by cooperating with CCCTC binding factor (*CTCF*) and DEAD‐Box Helicase 5 (*DDX5*). Using absolute quantitative lipidomics analysis, we found a hepatocyte‐specific *DDX17* deficiency decreased lipid accumulation and altered lipid composition in the livers of mice after MCD administration. Furthermore, combined with RNA‐seq analysis, lipidome analysis and rescue experiments, we found that *DDX17* potentially contributes to the control of lipid metabolism and activation of M1 macrophages by regulating the expression of *Cyp2c29* in murine NASH models (Additional files 1). Collectively, our study reveals a novel mechanism and offers promising targets for the treatment of hepatic steatosis, inflammation and fibrosis.

## 2 MATERIALS AND METHODS

### 2.1 Clinical specimens

Human liver tissues were acquired from individuals without steatosis, with NAFLD or NASH, who had undergone a liver transplantation. The detailed flowchart is presented in Additional files 2. Normal liver, NAFLD and NASH were diagnosed by two pathologists based on the scoring system established by the NASH Clinical Research Network.^19^ The normal liver was defined as cases with a NASH activity score (NAS) of 0. Simple steatosis was determined by NAS of 1−2, a ballooning score of 0 and the absence of fibrosis. NASH was defined as the cases with NAS ≥ 5 or the presence of fibrosis, NAS of 3−4. Clinical and histological characteristics of the patients are presented in Table S1. Subjects or the immediate families of liver donors provided written informed consent. The research protocols related to human samples received ethical clearance from the Review Board at Tongji Hospital, Huazhong University of Science and Technology, Wuhan, China, and adhered to the ethical principles set forth in the Declaration of Helsinki.

### 2.2 Animal models and treatment

Hepatocyte‐specific Mouse *Ddx17* conditional knockout mouse strains (*Ddx17*‐CKO) were acquired from Cyagen (CKOAI190719XW1‐B; Cyagen). Male 6−8 week‐old mice were subjected to either a HFD (Research Diets, NJ, USA; catalog number D12942) or a ND treated as controls for 24 weeks. The MCD model was fed with an MCD diet (Research Diets; A02082002BR/A06071301B) for 16 weeks. Meanwhile, 8‐week‐old male Ob/ob and lean mice that were purchased from Beijing HFK Bioscience (Beijing, China) were fed with a ND. All mice were housed in a controlled environment with temperatures maintained at 22−24°C and subjected to a 12‐h light–dark cycle. Mice were provided with humane care in accordance with guidelines established by the National Institutes of Health. All experiments involving mice were performed with approval from the Tongji Hospital Animal Care and Use Committee (TJH‐202101106).

### 2.3 Cell culture

293T cells, L02 cells and RAW264.7 macrophages were cultured in an environment with 5% CO_2_, DMEM containing 10% foetal bovine serum (FBS) and 1% penicillin–streptomycin for their growth. Murine AML12 hepatocytes were cultured in DMEM/F12 supplemented with 10% FBS, 40 ng/mL dexamethasone and 1% ITS (Table S3). The cell lines utilised in our study were obtained from the China Center for Type Culture Collection, and we verified their authenticity via STR analysis.

### 2.4 Co‐culture system

The indicated AML12 hepatocytes (1 × 10^5^/mL) and RAW264.7 macrophages (5 × 10^5^/mL) were respectively seeded into six‐well culture plates. After 24 h, the culture supernatants of indicated AML12 hepatocytes were transferred into the RAW264.7 with indicated treatments.

### 2.5 Induction of the lipid accumulation in hepatocytes

L02 hepatocytes were cultured in a suitable medium, and then they were exposed to palmitic acid and oleic acid (PAOA) at specified concentrations in a 1:2 ratio for a duration of 12 h. We used bovine serum albumin (BSA) without fatty acids as the control.

### 2.6 Plasmid construction, transfections and stable cell line construction

Related plasmids used in this study were constructed according to Vazyme ’s homologous recombination method (Cat: C112). In brief, the linearised vector was obtained by digesting the pCDNA3.1− circular vector with indicated resection enzymes. *DDX17*, *DDX5* and *CTCF* genes were cloned into the pcDNA3.1− vector after respectively amplifying by PCR. And before transfection, all constructs were verified through DNA sequencing. Prior to transfection, DNA sequencing was performed to validate all constructs. Subsequently, using the Lipo3000 transfection reagent for plasmids transfection, recombinant lentiviruses with Flag‐tagged *DDX17* were obtained from GENECHEM Co., Ltd, Shanghai. pLKO.1 plasmid was used to construct the gene knockdown stable cell lines. A detailed method for the production of lentiviral or retroviral supernatants was described previously.^18^ Briefly, 293T were transfected with the plasmids pMD2.G, psPAX2, shRNA plasmids by Lipofectamine 3000. For the formation of stable cell lines, hepatocytes underwent lentiviral transfection for at least 24 h and selected with 2.5–5 μg/mL puromycin or corresponding resistance medium for at least 7 days. The effect of gene overexpression and knockdown was validated by RT‐PCR and WB. Detailed information on plasmids was listed in Table S3.

### 2.7 Histological analysis

The haematoxylin and eosin (H&E) was stained to represent the accumulation of lipid and inflammation status using the liver sections. The NASs were calculated similarly to clinical specimens. Masson trichrome (MT) and Picrosirius red staining (PSR) were performed to visualise the content and type of fibres in the paraffin‐embedded sections. The histological features were obtained using the light microscope (Nikon ECLIPSE 80i, Japan). For Oil red O staining, use OTC to embed liver tissue and stain these slides with oil red O working solution, and then these slides were scanned using digital slide scanner (NanoZoomer S360; Hamamastu). For liver tissues immunohistochemistry (IHC), paraffin sections (4 μm thick) were prepared to deparaffinise and rehydrate by xylene and ethanol. Subsequently, endogenous peroxidase was inactivated using the 0.3% hydrogen peroxide. The detailed experimental procedures were executed following the protocol of the ZSGB‐BIO kit. The immunohistochemical staining scores were analysed by combining the staining intensity score with the proportion of masculine cells as described previously.^20^ For the histochemical staining score of the clinical NAFLD patient samples, the tissue sections underwent scoring, considering both the intensity of staining (ranging from 0 to 3 points, corresponding to non‐staining, light yellow, light brown and dark brown, respectively) and the extent of positive staining (ranging from 0 to 25, 0 to 25, 26 to 50, 51 to 75 and 76−100%, respectively, rated from 1 to 4 points), and then the two scores were added into the final score. For the histochemical staining score of mouse liver tissue sections, the software Image J was utilised for the quantification of the stained area in the images and the positive staining area was recorded for subsequent analysis. The related primary antibodies are specifically listed in Table S4.

### 2.8 Immunofluorescence analysis

*Immunohistochemical fluorescence*: Tissue sections were incubated with 10% BSA for 1 h at 37°C after the antigen retrieval, and subsequently, primary antibodies were applied and left to react overnight at 4°C. The primary antibodies are listed in Table S4. Then, tissue sections were exposed to goat anti‐rabbit immunoglobulin for 1 h. Nuclei were stained by DAPI. Images were acquired using the fluorescence microscope (Pannoramic MIDI; 3DHISTECH) and quantified using Image J software. *Immunofluorescence* (IF): Cells were fixed with paraformaldehyde after co‐transfected with indicated plasmids for 48 h. Then, cells were subjected to incubation with the specified primary antibodies after being permeabilised with 0.5% NP‐40 (Nonidet P 40) and blocked with 5% goat serum albumin, and subsequently being incubated fluorescein‐conjugated secondary antibodies for 1 h. DAPI is used for staining cell nuclei. Images were obtained under the fluorescent microscope (Nikon Digital ECLIPSE C1, Japan).

### 2.9 Oil Red O staining

Indicated cells were cleaned using 60% isopropyl alcohol, then dyeing with Oil red O working solution for about 10−30 min. Then, the stained cells were rinsed with 75% ethanol and fixed by glycerol after being washed by PBS.

### 2.10 Mouse metabolic studies

During the experiments, body weight (BW) was assessed at different time points, and liver weight (LW) was assessed at the end point. In the glucose tolerance test (GTT) experiment, mice were subjected to a 16‐h fast followed by intraperitoneal glucose (1.5 g/kg) injection. And in the insulin tolerance test (ITT) experiment, mice were fasted for 6 h before intraperitoneal insulin (0.5 U/kg) injection. Subsequently, blood glucose levels were measured every half hour continuously for 2 h post‐administration for both the GTT and ITT. We utilised the animal monitoring system (Columbus Instruments, USA) to track the energy expenditure of the mice. Over a 48‐h period, we recorded metabolic parameters, including oxygen consumption (VO_2_), carbon dioxide emission (VCO_2_) and food intake, in a single‐chambered enclosure, following established procedures.^21^

### 2.11 Mouse serum and liver assay analyses

The levels of serum alanine transaminase (ALT), aspartate aminotransferase (AST), total triglycerides (TG) and total cholesterol (TC) were measured on an Chemray 240 and Chemray 800 (Rayto Life and Analytical Sciences Co., Ltd.) according to the instructions. The hepatic TG and TC contents were extracted and measured with the detection kit (C061 and C063; Changchun Huili Biotech Co., Ltd) according to the guidance.

### 2.12 Western blot analysis

Western blotting analyses were conducted following a standard protocol. The primary antibodies are listed in the Table S4. Three different primary antibodies were used to detect different expression of DDX17. Proteintech (19910‐1‐AP) was used in the western blot and co‐immunoprecipitation (COIP), Abcam (ab70184) was used in human liver in IHC and the Bethyl Laboratories (A300‐509A) was used in the livers of mice. Each experiment has been repeated for three times.

### 2.13 RNA isolation and RT‐qPCR

Total RNA was isolated from liver tissues and indicated cells using TRIzol™ Reagent and a real‐time fluorescent quantitative PCR assay was conducted following its instructions. The exact method and analysis of RT‐qPCR was described previously^18^ and Table S5 has listed all primer information.

### 2.14 | RNA‐sequencing and analysis

The liver tissues from the *DDX17*‐Flox mice and *DDX17*‐CKO mice after MCD administration were lysed in TRIzol™ Reagent. Total RNA extraction was accomplished using TRIzol™ Reagent from Invitrogen™, followed by a real‐time fluorescent quantitative PCR assay to confirm the knockout efficiency. Subsequently, the samples were sent to Novogene Bioinformatics Technology Co. Ltd (Beijing, China) for further processing. The transcriptome sequencing and subsequent analysis were conducted by Novogene. In a concise summary, the PCR products were subjected to purification utilising the AMPure XP system, and the quality assessment of the library was performed through the utilisation of the Agilent Bioanalyzer 2100 system. Genes were considered as differentially expressed if *p* < .05 assessed by DESeq2. Differentially expressed genes (DGEs) were analysed by Gene Ontology (GO) enrichment analysis with the R package. The statistical enrichment of DGEs was acquired by using the cluster Profiler R package in KEGG pathways. Reactome pathways of DGEs (*p* < .05) were performed by combining the human model species' reactions and biological pathways. GSEA was implemented using GSEA analysis tool, GO, KEGG and Reactome. Related results were listed in Tables S6–S8.

### 2.15 CUT&Tag analysis

The CUT&Tag experiments, including the construction of DNA library, were carried out according to the protocol of hyperactive Universal CUT&Tag Assay Kit (TD903‐01/02, vazyme). Briefly, cells were harvested and counted (100,000 cells). The genomic DNA (gDNA) samples were eluted using 26 μL of Millipore water, quantified with the Onedrop system, and then made ready for library amplification. The amplification cycles were 16 according to the index of Illumina (Vazyme#TD202‐01) and the products were purified by 1.2× magnetic beads (Vazyme#N411‐01), and the final library elution volume was 22 μL. The cDNA libraries underwent sequencing on the Illumina Novaseq 6000, employing 150‐bp paired ends and delegated to Novogene Biotech Co., Ltd. (Beijing China) after the size was determined by the Agilent 2100 Tape Station analysis. Detailed analysed results were listed in the final report of biological information analysis (Additional files 3: X101SC21061883‐Z01‐J001‐B1‐36)

### 2.16 Absolute quantitative lipidomics

The liver tissues from the *DDX17*‐Flox mice and *DDX17*‐CKO mice after MCD administration were consigned to Applied Protein Technology (Shanghai, China) to perform the lipid extraction and mass spectrometry‐based lipid detection. In each group, six replicates were mixed equally as the QC sample. QC samples were conducted to assess the system stability and data reliability during the process. Samples were divided by the UHPLC Nexera LC‐30A (SHIMADZU), followed by LC–MS/MS analysis. The lipid identification, peak extraction with alignment and quantification of lipid molecules and internal standard lipid molecules were assessed using the Lipid Search software (Thermo Scientific™). In this experiment, the stability of devices, the repeatability of tests and the reliability of the data were fully evaluated by six quality control items. Detailed data analysis including the lipid class, lipid species, lipid composition, lipid concentration and so on were conducted by Applied Protein Technology and shown in the Additional files 4.

### 2.17 ChIP assays

The ChIP assays were conducted following the protocol outlined in the SimpleChIP® Plus Sonication Chromatin IP Kit (CST, #56383). Before harvested, cells were washed with prechilled PBS, and then followed by incubation in 1% formaldehyde for 15 min at RT for chromatin cross‐linking. Then, the cell pellets were lysed in the specific lysis buffer prepared according to the protocol and then sonicated to obtain 200–500 bp DNA fragments. Immunoprecipitations were carried out using anti‐DDX17, anti‐Histone 3 and normal rabbit IgG. Using RT‐PCR to detect the immunoprecipitated DNA fragments with the validated primers, which were predicted and designed for the region of cyp2c29 promoters were listed in Table S5.

### 2.18 Dual‐Luciferase reporter assay

Briefly, indicated cells were seeded in a 24‐well plate with 5 × 10^4^ per well and cultured for 24 h. Subsequently, we performed a co‐transfection of the promoter reporter plasmids (0.48 μg) and the pRL‐TK plasmids (0.02 μg) into the cells using Lipofectamine 3000 from Invitrogen. After 6 h, the culture medium was changed with fresh medium for 48 h. Then, we quantified luciferase activity utilising a GloMax 20/20 Luminometer manufactured by Promega. To ensure accuracy and consistency, we normalised the luciferase activity to the Renilla activity by following the prescribed protocol of the Dual‐Luciferase Reporter Assay System (Promega, Madison, WI, USA).

### 2.19 Co‐immunoprecipitations

Cells were lysed using IP‐lysis buffer (P0013; Beyotime Biotechnology) and protease inhibitor cocktail (Roche). The following steps were conducted following a standard protocol.

### 2.20 Statistical analysis

We conducted statistical analysis by employing GraphPad Prism software. To compare two sets of data, we utilised a two‐tailed Student's *t*‐test. For the comparisons involving multiple groups, we executed a one‐way ANOVA. Quantitative data were analysed using Spearman's correlation when applicable. Statistical significance was established as follows: *p < .05; **p < .01; ***p < .001.

## 3 RESULTS

### 3.1 *DDX17* expression is enhanced as NASH progresses

**FIGURE 1:**
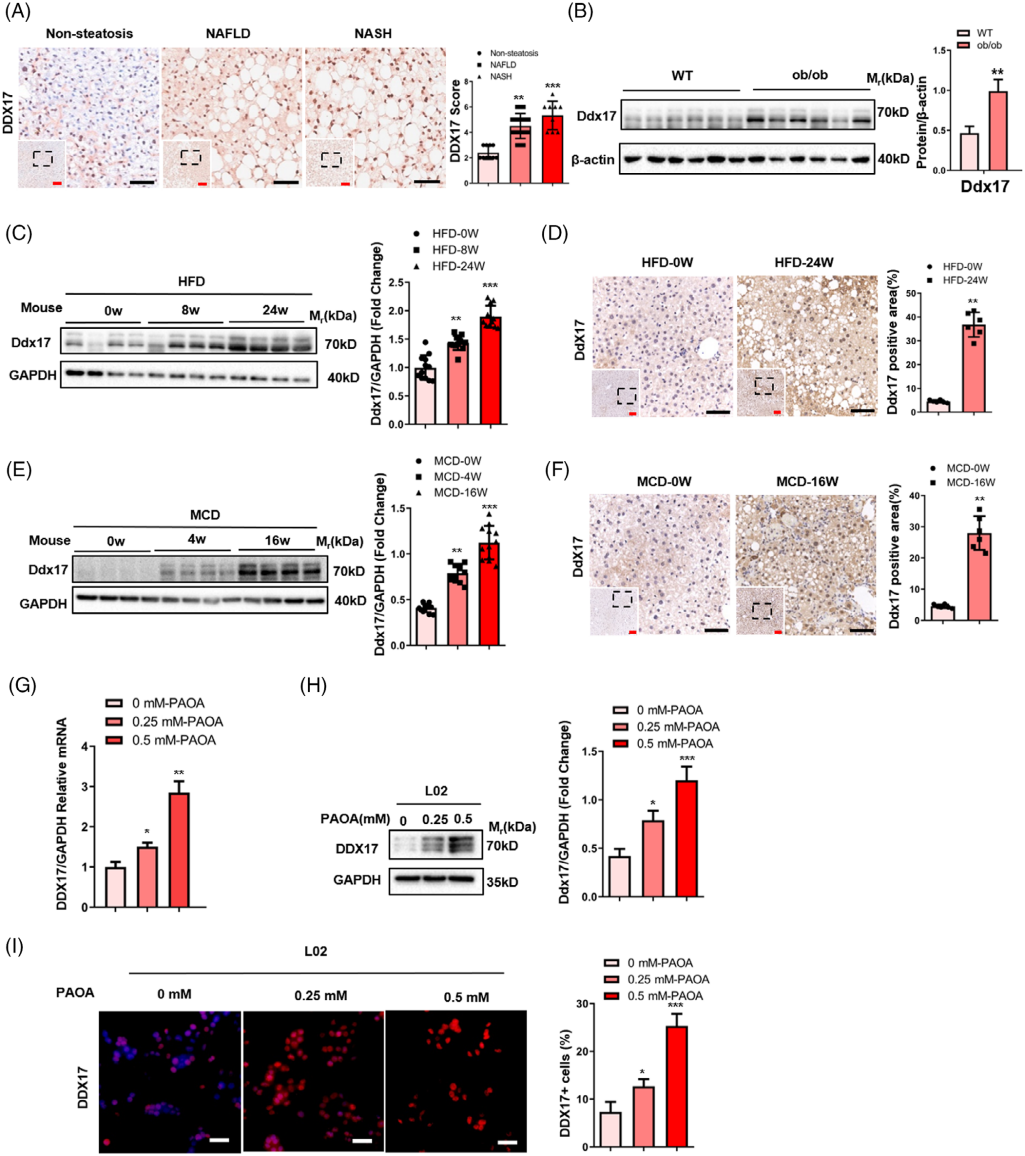
*DDX17* expression is up‐regulated in fatty liver and correlates with NASH progression. (A) Representative IHC images (left) and corresponding quantification (right) of *DDX17* expression in the livers of subjects without steatosis (non‐steatosis, *n* = 10), with NAFLD (NAFLD, *n* = 17) or with NASH (NASH, *n* = 9) (**p < .01, ***p < .001; red bar, 100 μm; black bar, 50 μm). (B) Representative western blots (WB) (left) and quantification (right) of *Ddx17* expression in the livers of C57BL/6J wild‐type (WT) mice and ob/ob (leptin‐deficient) mice fed for 8 weeks (**p < .01; *n* = 6 per group). (C) Representative WB (left) and quantification (right) of *Ddx17* expression in the livers of C57BL/6J mice that were fed with a high‐fat diet (HFD) for 0, 8 and 24 weeks (**p < .01, ***p < .001; *n* = 4 western blots). (D) Representative IHC images (left) and quantification (right) of *Ddx17* expression in the livers of C57BL/6J mice treated with normal diet (ND) or a HFD for 24 weeks (**p < .01, Red bar, 100 μm; Black bar, 100 μm; *n* = 6 per group). (E) Representative WB (left) and quantification (right) of *Ddx17* expression in the livers of C57BL/6J mice that were fed with a methionine and choline‐deficient l‐amino acid diet (MCD) for 0, 4 and 16 weeks (**p < .01, ***p < .001; *n* = 4 western blots). (F) Representative IHC images (left) and quantification (right) of *Ddx17* expression in the livers of C57BL/6J mice treated with ND or a MCD for 16 weeks (**p < .01; red bar, 100 μm; black bar, 100 μm; *n* = 6 per group). (G–I) The mRNA levels (G), representative WB (H) and immunofluorescence (IF) (I) of *DDX17* expression in L02 hepatocytes that was treated with palmitic acid and oil acid (PAOA: PA; 0.25 mM, OA; 0.5 mM) for 24 h (*p < .05, **p < .01, ***p < .001; white bar, 50 μm). For (B), β‐actin was used as the loading control. For (C) and (E), GAPDH was used as the loading control. For (A), (C), (E) and (G–I), statistical analysis was carried out by one‐way ANOVA. For (B), (D) and (F), statistical analysis was carried out by two‐tailed Student's *t*‐test. All data are shown as the mean ± SD.

Hepatic DDX17 protein levels were elevated in patients with NAFLD or NASH than in those with non‐steatosis. Furthermore, compared with the non‐steatosis group, the NAFLD and NASH group demonstrated substantially higher *DDX17* expression (Figure 1A) in the quantification of IHC. To prepare for *DDX17* causation studies, we explored *DDX17* expression in various NASH mouse models. Consistent with results in human subjects, the levels of *DDX17* protein and mRNA were significantly up‐regulated in the livers of HFD‐fed, MCD‐fed and ob/ob mice during NASH progression (Figures 1B–F and S1A–D). Then, L02 cells were stimulated by PAOA to investigate the mechanism underlying the up‐regulation of *DDX17* expression. We found that PAOA (PA, 0.25 mM; OA, 0.5 mM) treatment increased the mRNA and protein expression levels of *DDX17* in L02 cells (Figures 1G–I and S1E). The findings from this study indicate a potential association between *DDX17* and NASH and *DDX17* potentially exerting a crucial influence on its in its progression.

### 3.2 *DDX17* promotes hepatic steatosis in murine NASH models

Next, we infected L02 cells with lentivirus *DDX17* overexpression and *DDX17*‐knockdown clones (Figures S2A and 2E) in order to investigate how DDX17 impacts lipid metabolism in hepatocytes. Oil Red O staining, cellular TG and cellular TC analysis showed a distinct increase in lipid accumulation induced by PAOA in the DDX17‐overexpressing group and a significant decrease in the DDX17 knockdown group compared with the control group. There were no statistically significant distinction observed between the two groups when exposed to BSA (Figures S2B–D and S2F–H). In order to assess the distribution of *DDX17* in the liver, we first performed bioinformatic analyses of single‐cell RNA sequencing (scRNA‐seq) data of the human liver (GSE115469) to detect the characteristics of *DDX17* expression in human liver cells. The results indicated that in a normal liver, the expression of *DDX17* is low to moderate in hepatocytes, Kupffer cells, hepatic stellate cells (HSCs), endothelial cells, cholangiocytes, erythroid cells, T‐cells and B‐cells (Figure S3A). In order to investigate the function of hepatocyte *DDX17* in NASH in vivo, we constructed hepatocyte‐specific *DDX17*‐knockout mice (*DDX17*‐CKO) and subjected them to a chow diet or a HFD for 24 weeks. Subsequently, the mice underwent metabolic assessments utilising a Comprehensive Lab Animal Monitoring System. Comparing the DDX17‐CKO mice with the DDX17‐Flox mice, differences observed in terms of oxygen consumption (Figure S3B), carbon dioxide emission (Figure S3C) or food intake (Figure S3D) were not significant.

**FIGURE 2:**
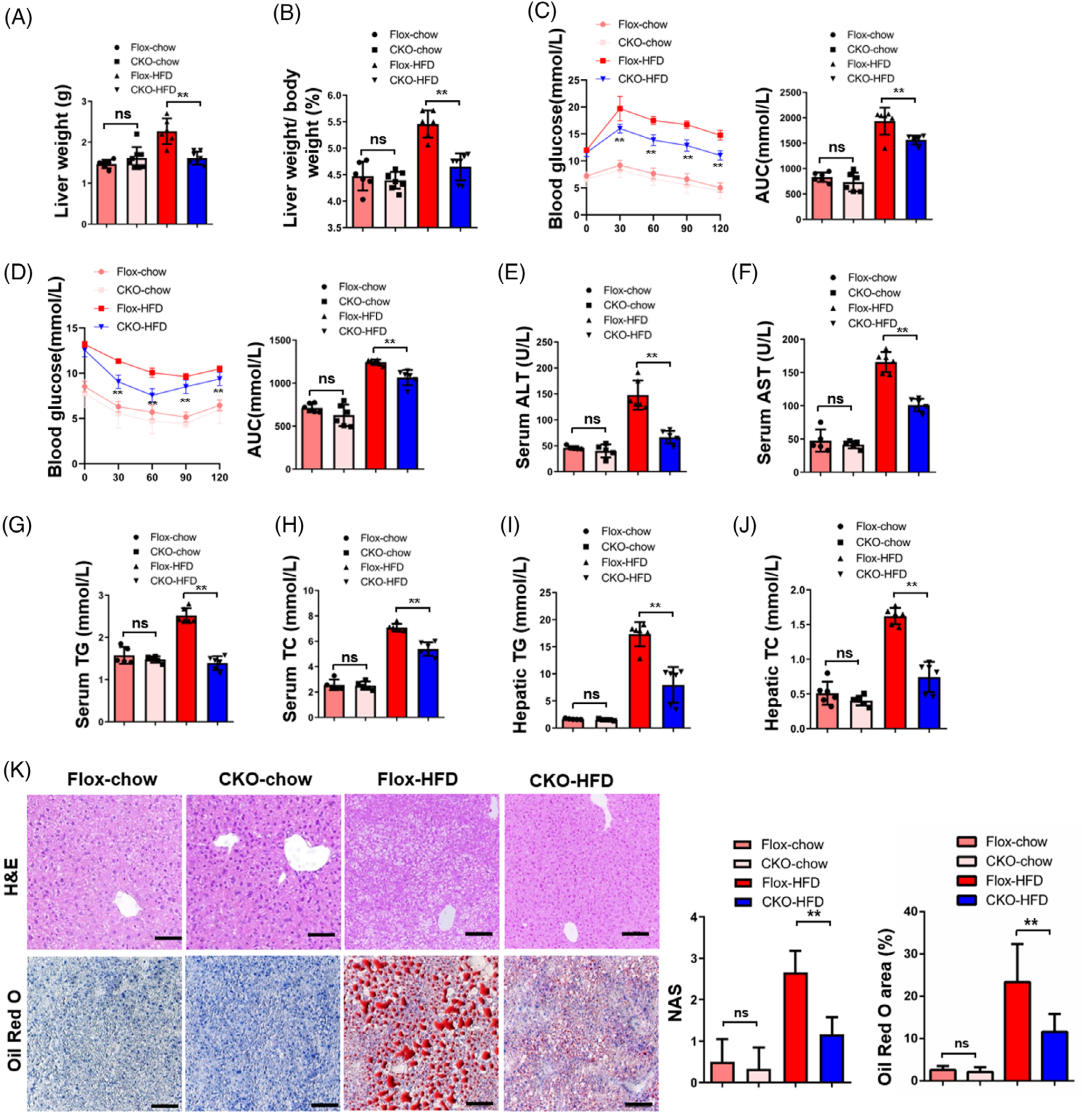
Hepatocyte‐specific *DDX17* deficiency alleviates HFD diet‐induced hepatic steatosis. (A and B) Liver (A) and liver weight/body weights (B) of *DDX17*‐Flox and *DDX17*‐CKO mice were fed with chow diet or HFD for 24 weeks (**p < .01; n.s., not significant; *n* = 6 mice/group). (C and D) Blood glucose levels in the glucose tolerance test (GTT) (C) and the insulin tolerance test (ITT) (D) in *DDX17*‐Flox and *DDX17*‐CKO mice that were fed with chow diet or HFD for 24 weeks (**p < .01; n.s., not significant; *n* = 6 mice/group). (E–H) Serum ALT (E), serum AST (F), serum total triglyceride (TG) (G) and total cholesterol (TC) (H) levels from *DDX17*‐Flox and *DDX17*‐CKO mice that were fed with chow diet or HFD for 24 weeks (**p < .01; n.s., not significant; *n* = 6 mice/group). (I and J) Hepatic TG (I) and hepatic TC (J) levels from *DDX17*‐Flox and *DDX17*‐CKO mice that were fed with chow diet or HFD for 24 weeks (**p < .01; n.s., not significant; *n* = 6 mice/group). (K) Representative images (left) and quantitative results (right) of haematoxylin and eosin (H&E)‐stained and Oil Red O‐stained liver sections from *DDX17*‐Flox and *DDX17*‐CKO mice that were fed with chow diet or HFD for 24 weeks (**p < .01; n.s., not significant; *n* = 6 mice/group; scale bar, 100 μm). In all statistical plots, data are statistically analysed using the one‐way ANOVA and shown as the mean ± SD.

After 24 weeks of HFD consumption, the CKO‐HFD group exhibited a significant reduction in BWs, LWs and liver‐to‐BW ratios as compared with the Flox‐HFD control group (Figures S4A, 2A and B). The GTT demonstrated improved glucose tolerance, while the ITT indicated less insulin resistance in CKO‐HFD mice compared with Flox‐HFD mice (Figures 2C and D). In CKO‐HFD mice, the serum concentrations of ALT, AST, TG and TC were found to be lower in comparison with those observed in Flox‐HFD mice (Figures 2E–H). Furthermore, Flox‐HFD mice shown more severe steatosis in the liver than CKO‐HFD mice, which was supported by hepatic TG and TC concentrations (Figures 2I and J), H&E and Oil Red O staining (Figures 2K and S4B). These findings imply that *DDX17* may contribute to the accumulation of lipids in hepatocytes and promote NASH progression in mice.

### 3.3 Hepatocyte‐specific *DDX17* deficiency alleviates MCD‐induced NASH

**FIGURE 3:**
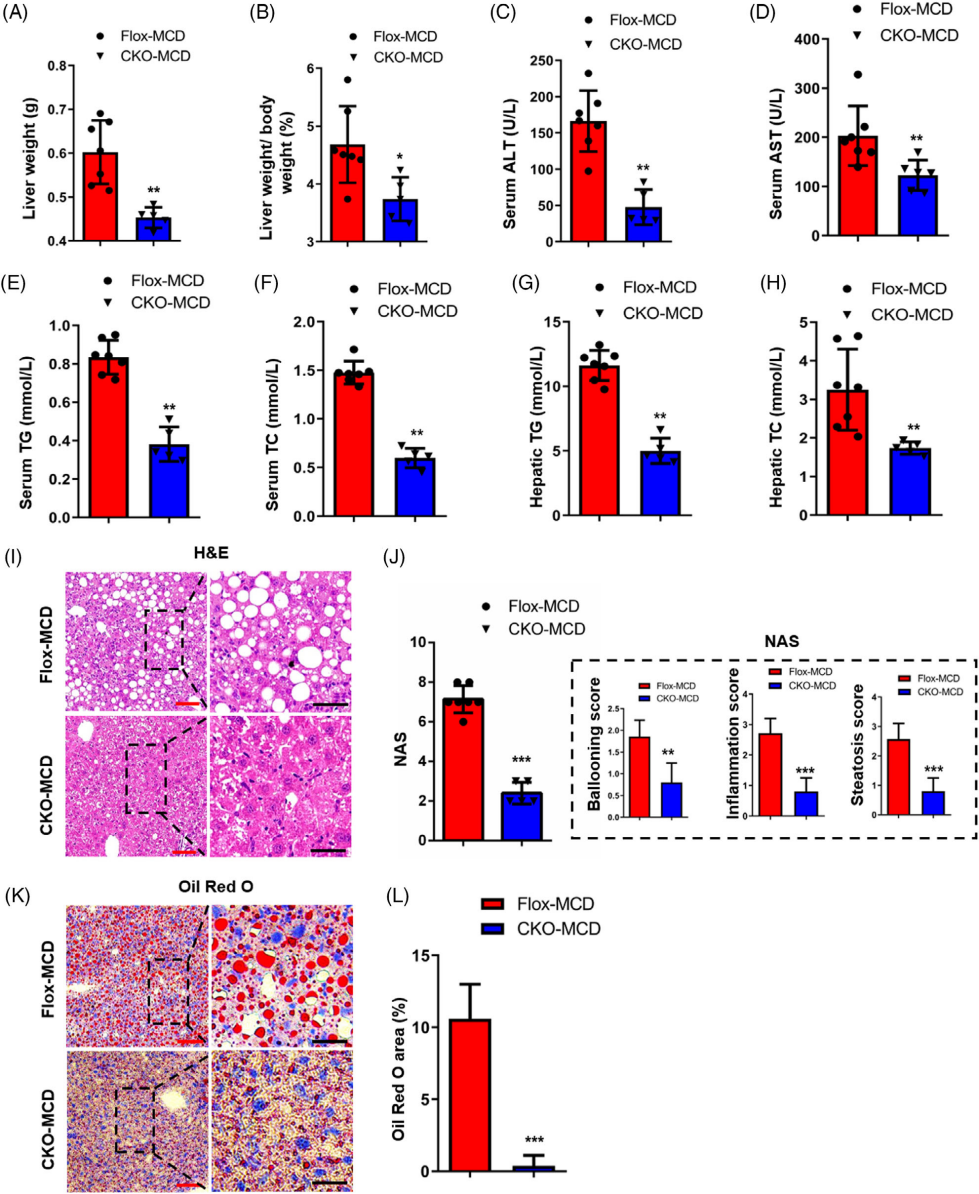
Hepatocyte‐specific *DDX17* deficiency alleviates MCD diet‐induced hepatic steatosis. (A and B) Liver (A) and liver weight/body weights (B) of Flox‐MCD and CKO‐MCD mice were fed with MCD for 16 weeks. (C–F) Serum ALT (C), serum AST (D), serum TG (E) and TC (F) levels from Flox‐MCD and CKO‐MCD mice that were fed with MCD for 16 weeks. (G and H) Hepatic TG (G) and hepatic TC (H) levels from Flox‐MCD and CKO‐MCD mice fed with MCD for 16 weeks. (I–L) Representative images (left) and quantitative results (right) of H&E‐stained (I) and Oil Red O‐stained (K) liver sections from Flox‐MCD and CKO‐MCD mice fed with MCD for 16 weeks (red bar, 100 μm; black bar, 100 μm). For the above analysis, Flox‐MCD group (*n* = 7 mice/group) and CKO‐MCD group (*n* = 5 mice/group) were compared (*p < .05; **p < .01; ***p < .001). In all statistical plots, data are statistically analysed using the two‐tailed Student's *t*‐test and shown as the mean ± SD.

To further validate the role and precise mechanism of hepatic DDX17 in the development of NASH, we subjected *DDX17*‐Flox and *DDX17*‐CKO mice to a MCD diet for 16 weeks, which were respectively labelled as Flox‐MCD and CKO‐MCD mice for short. After these 16 weeks, the CKO‐MCD mice showed significantly lower BWs, LWs and liver‐to‐BW ratios than the Flox‐MCD controls (Figures S5A, 3A and B). Significantly higher oxygen consumption (Figure S5B), carbon dioxide emission (Figure S5C) and food intake (Figure S5D) were noted in the *DDX17*‐CKO mice compared with those of *DDX17*‐Flox mice. The levels of ALT, AST, TG and TC in the liver were decreased in CKO‐MCD mice compared with Flox‐MCD mice (Figures 3C–F). Furthermore, Flox‐MCD mice exhibited more severe steatosis in the liver than CKO‐MCD mice, as supported by the concentrations of hepatic TG and TC (Figures 3G and H) as well as H&E and Oil Red O staining (Figures 3I–L).

### 3.4 AAV8‐mediated hepatic DDX17 overexpression aggravates MCD‐induced NASH

**FIGURE 4:**
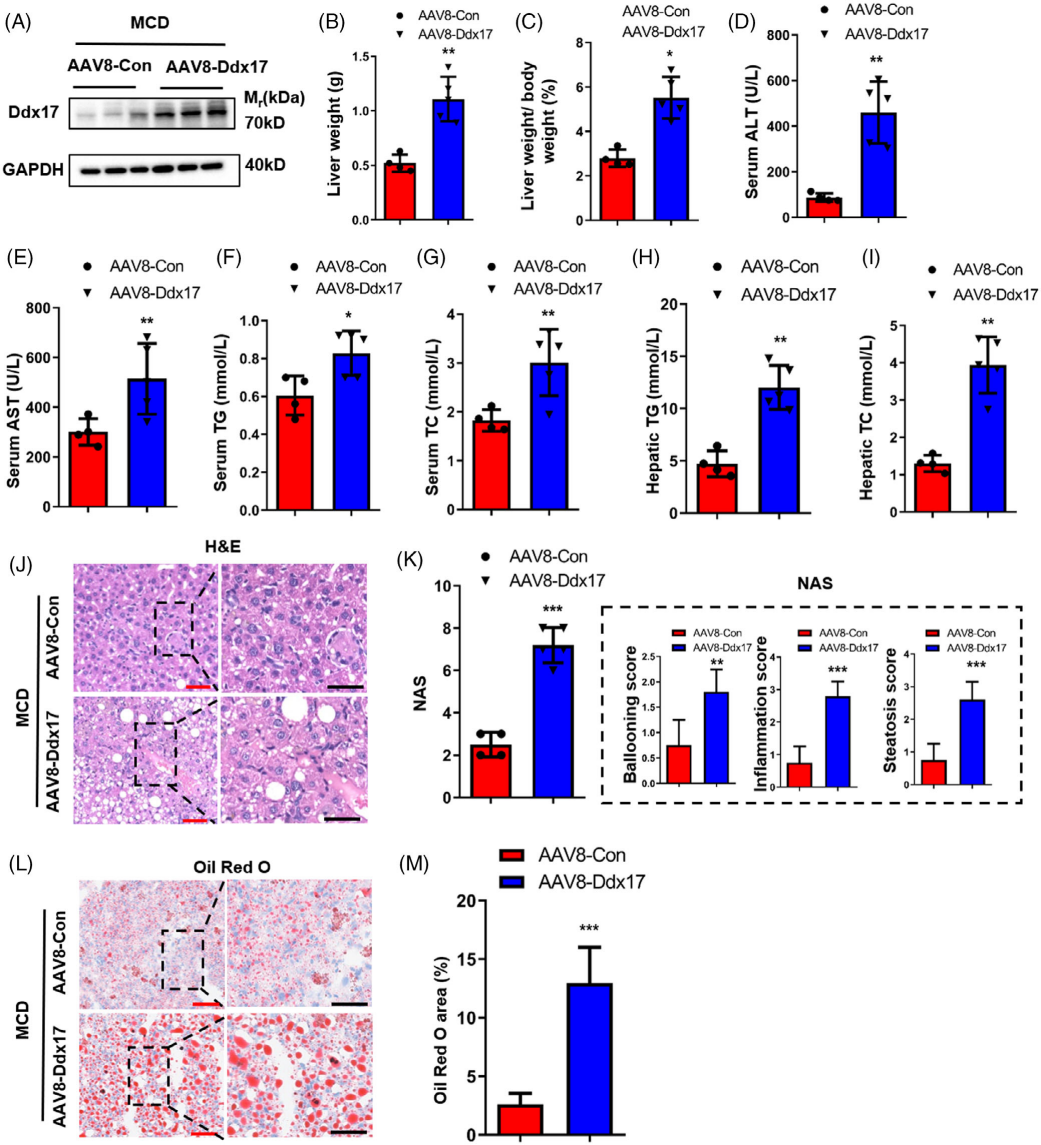
AAV8‐mediated hepatic DDX17 overexpression aggravates MCD diet‐induced hepatic steatosis. (A) DDX17 expression in the livers of AAV8‐Con and AAV8‐Ddx17 mice. The WT mouse infected by adeno‐associated virus (AAV) carrying Ddx17 (AAV8‐Ddx17) or the control virus (AAV8‐Con). (B and C) Liver (B) and liver weight/body weights (C) of AAV8‐Con and AAV8‐Ddx17 mice were fed with MCD for 16 weeks. (D–G) Serum ALT (D), serum AST (E), serum TG (F) and TC (G) levels from AAV8‐Con and AAV8‐Ddx17 mice that were fed with MCD for 16 weeks. (H and I) Hepatic TG (H) and hepatic TC (I) levels from AAV8‐Con and AAV8‐Ddx17 mice fed with MCD for 16 weeks. (J–M) Representative images (left) and quantitative results (right) of H&E‐stained (J) and Oil Red O‐stained (L) liver sections from AAV8‐Con and AAV8‐Ddx17 mice fed with MCD for 16 weeks (red bar, 100 μm; black bar, 100 μm). For the above analysis, AAV8‐Con group (*n* = 4 mice/group) and AAV8‐Ddx17 group (*n* = 5 mice/group) were compared (*p < .05; **p < .01; ***p < .001). In all statistical plots, data are statistically analysed using the two‐tailed Student's *t*‐test and shown as the mean ± SD.

To further validate the role of hepatocyte *DDX17* in the development of NASH, we overexpressed DDX17 in 6‐week‐old mice by injection of an adeno‐associated virus expressing Ddx17 (AAV8‐Ddx17) in parallel with its control followed by MCD feeding for another 16 weeks (Figure 4A). After these 16 weeks, the AAV8‐Ddx17 mice exhibited notable increased LWs and liver‐to‐BW ratios in comparison with the AAV8‐Con mice (Figures 4B and C). The AAV8‐Ddx17 mice exhibited elevated serum levels of ALT, AST, TG and TC in comparison with the AAV8‐Con mice within the liver samples (Figures 4D–G). Furthermore, AAV8‐Ddx17 mice displayed a more pronounced liver steatosis than AAV8‐Con mice, as supported by the concentrations of hepatic TG and TC (Figures 4H and I) as well as H&E and Oil Red O staining (Figures 4J–M). These findings suggest that *DDX17* could be involved in lipid accumulation in hepatocytes and promote the NASH progression in an MCD‐induced mouse model.

### 3.5 *DDX17* regulates lipid metabolism and the subsequent inflammatory response by regulating the CYP450 family genes

**FIGURE 5:**
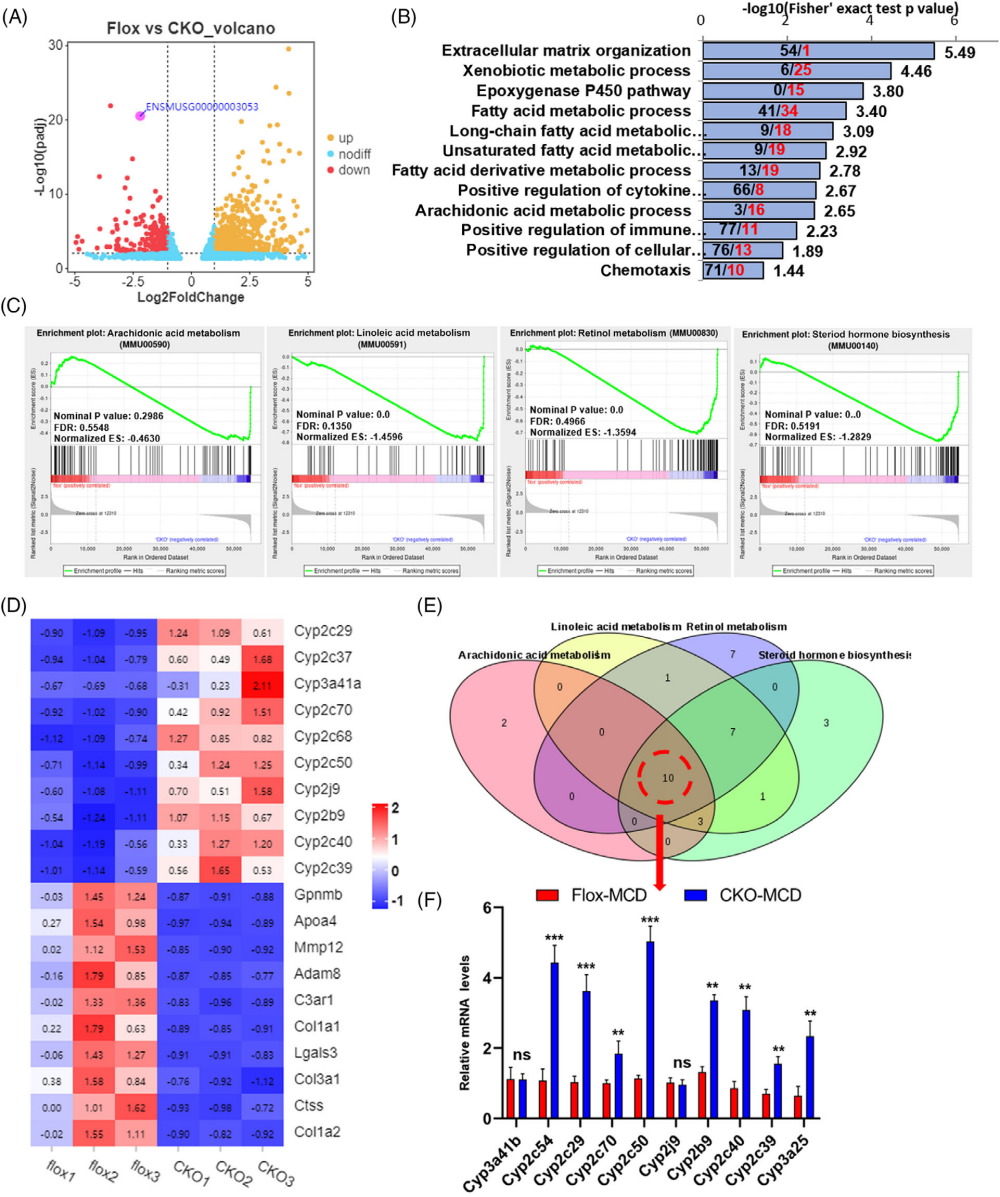
*DDX17* may alter the landscape of lipid metabolism, inflammation and fibrosis by regulating the expression of CYP450 family genes in murine NASH. (A) Volcano plots indicating the DEGs (red, down‐regulated genes; yellow, up‐regulated genes) between *DDX17*‐Flox and *DDX17‐*CKO mice were fed with MCD for 16 weeks. (B) GO function enrichment analysis of differential up‐regulated genes in MCD‐induced steatohepatitis in hepatocyte‐specific *DDX17* knockout mice. Down/up‐regulated genes enriched in pathways were indicated (black font, down; red font, up). (C) GSEA analysis of up‐regulated genes in *DDX17*‐CKO mice were mainly enriched in four metabolic pathways (arachidonic acid metabolism, linoleic acid metabolism, retinol metabolism and steroid hormone biosynthesis). (D) Heatmap analysis of up‐regulated and down‐regulated genes between *DDX17*‐Flox and *DDX17*‐CKO group. (E) Venn diagram analysis of genes in four differential metabolism related pathways was conducted to obtain ten CYP450 family genes. (F) Relative mRNA levels of ten CYP450 family genes in the livers of MCD‐fed *DDX17*‐CKO and *DDX17*‐Flox (**p < .01, ***p < .001; n.s., not significant). For (F), statistical analysis was performed using the two‐tailed Student's *t*‐test. All data are shown as the mean ± SD.

Next, we further elucidated the underlying mechanisms involved in the regulatory role of *DDX17* in promoting NASH. RNA‐seq of *DDX17*‐CKO and *DDX17*‐Flox groups that had been fed a MCD was conducted and the DEGs are displayed in volcano plots (Figure 5A). GO function enrichment analysis revealed that epoxygenase activity (arachidonic acid [AA] monooxygenase activity, oxidoreductase activity, etc.), related to metabolic pathways (xenobiotic stimulus, epoxygenase P450 pathway), inflammation (immune response, cytokine production) and extracellular matrix organisation, may be associated with the progression of NASH (Figures 5B and S6A). To further analyse the RNA‐seq results, we identified several lipid metabolism pathways, such as AA metabolism, retinol metabolism, steroid hormone biosynthesis and linoleic acid metabolism using GSEA analysis and a heatmap of the DEGs (Figures 5C and D). Ultimately, 10 CYP450 family genes with significant difference were obtained through Venn diagram analysis (Figure 5E) and validated by RT‐PCR (Figure 5F). Furthermore, the epoxygenase P450 pathway and positive regulation of cytokine production were further demonstrated via directed acyclic graph analysis of the differentially up‐regulated and down‐regulated genes in the two groups (Figures S6B–4D).

Based on the RNA‐seq analysis, our findings suggest that *DDX17* may play a role in lipid metabolism and in regulating the progression of inflammation by regulating the CYP450 family genes in MCD‐induced NASH.

### 3.6 *DDX17* transcriptionally repressed *Cyp2c29* gene expression by cooperating with *CTCF* and *DDX5* in L02 hepatocytes

**FIGURE 6:**
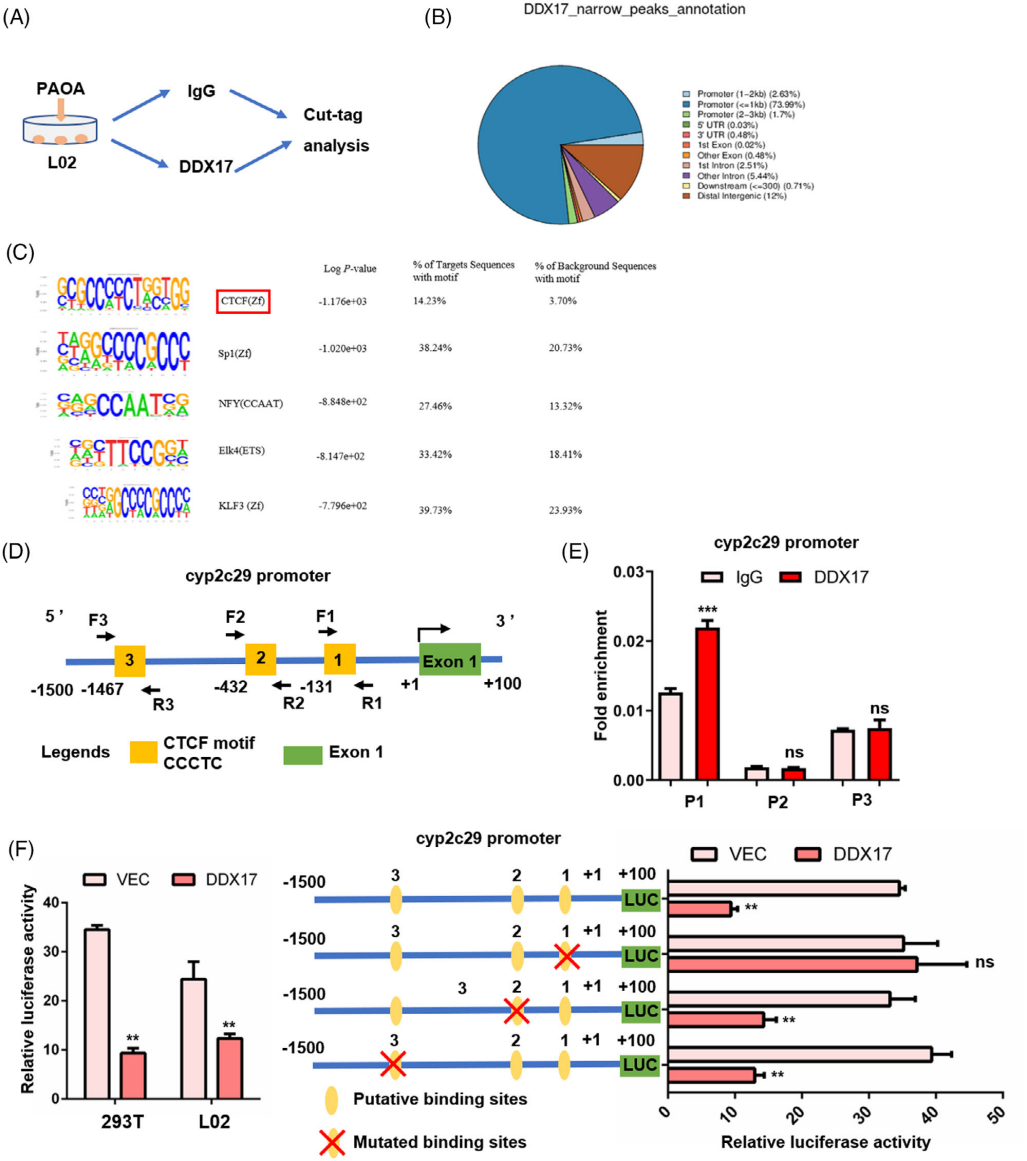
*DDX17* transcriptionally repress the *Cyp2c29* gene expression by binding the classic CTCF motif of its promoter. (A) A schematic diagram of CUT&Tag analysis in L02 hepatocytes treated with PAOA. (B) Genome‐wide peak annotation of *DDX17* showing the proportion of exon, intron, promoter, TSS and intergenic region were shown in the pie chart. (C) *DDX17* bound motifs identified by Homer known motifs in L02 cells. The motif sequence is shown in the first column, the corresponding transcription factor (TF) is shown in the second column, the *p* value is shown in third column and the proportion of target sequences and background sequences with motif are shown in the last two column. (D) The predicted binding sites of *DDX17* and *CTCF*. (E) The ChIP of L02 cells show the binding sites (***p < .001; *n* = 3 independent experiments). (F) The relative luciferase activity of wild‐type (left panel) or the sites‐mutated (right panel) *Cyp2c29* promoter was respectively detected in indicted cell groups (**p < .01; n.s., not significant; *n* = 3 independent experiments). For (E) and (F), statistical analysis was performed using the two‐tailed Student's *t*‐test.

Given the potential role of *DDX17* in inhibiting the expression of CYP450 family genes, we conducted a CUT&Tag of *DDX17* in L02 cells treated with PAOA. The genome‐wide peak annotation of *DDX17* indicated that the highest proportion of the promoter within 1 kb (Figures 6A, B and S7A) and the metabolic pathways and NAFLD were enriched in the pathway enrichment analysis of differential peak between IgG and *DDX17* groups (Figure S7B). Next, the bound motifs of *DDX17*, which were identified through HOMER known motifs, were shown (Figure 6C) and found to contain ‘CCCTC’, which has been previously reported to be the classic CTCF motif and is shared with the *DDX17* paralog, *p68* (*DDX5*).^22^, ^23^ Thus, we hypothesised that *DDX17* may bind to the *CTCF* motif in order to transcriptionally regulate *Cyp2c29* gene expression, which was found to be the most significantly up‐regulated gene in the *DDX17*‐CKO group. We then performed the ChIP assay to prove that *DDX17* binds to the promoter of *Cyp2c29*, especially the first ‘CCCTC’ motif close to exon 1 (Figures 6D–E and S7C), and the relative luciferase activity of the wild‐type or sites‐mutated *Cyp2c29* promoter was respectively detected in indicted L02 cell groups (Figure 6F).

**FIGURE 7:**
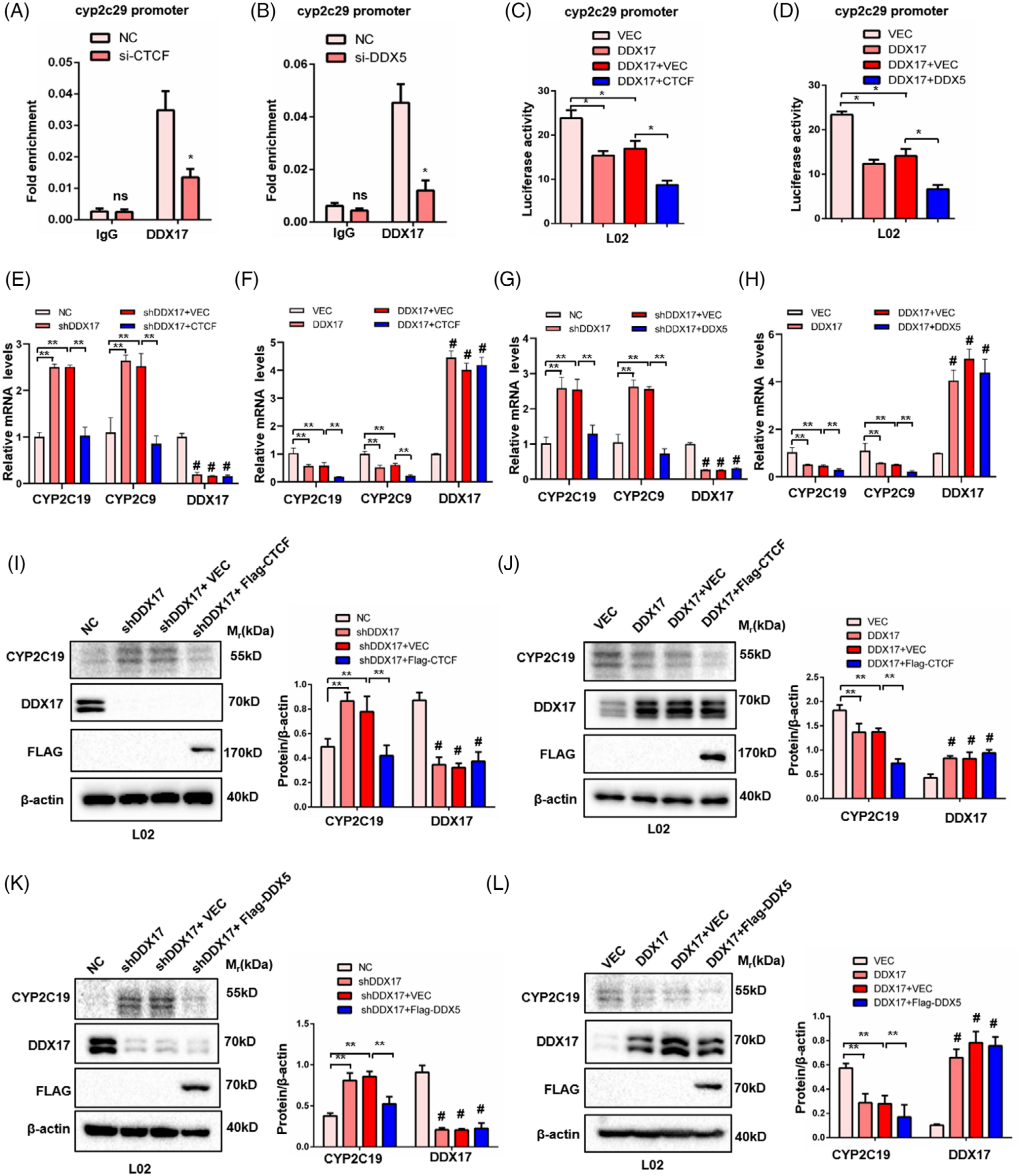
*DDX17* down‐regulate the gene expression of *Cyp2c29* through cooperating with *CTCF* and *DDX5* in L02 hepatocytes. (A and B) The ChIP of L02 cells transfected with *CTCF* (A) or *DDX5* (B) siRNA (*n* = 3 independent experiments). (C and D) The relative luciferase activity of *Cyp2c29* promoter was detected in indicted cell groups in L02 cells respectively transfected with *CTCF* (C) or *DDX5* (D) plasmid. (E–H) Relative mRNA levels of *CYP2C19*, *CYP2C9* and *DDX17* in indicated cell group in L02 hepatocytes. (I and J) Representative western blots (left) and quantification (right) of *CYP2C19*, Flag‐*CTCF* and *DDX17* in indicated cell group in L02 hepatocytes. (K and L) Representative western blots (left) and quantification (right) of *CYP2C19*, Flag‐*DDX5* and *DDX17* in indicated cell group in L02 hepatocytes. *p < .05; **p < .01; #*p* < .001; n.s., not significant, that was compared with the control group. Each experiment was repeated three times. For (A) and (B), statistical analysis was performed by two‐tailed Student's *t*‐test. For (C)–(L), statistical analysis was performed by one‐way ANOVA.

Previous studies have shown that *CTCF* regulates key aspects of gene expression, including transcription activation and repression.^23^, ^26^ Thus, we hypothesised that *DDX17* and its paralog, *DDX5*, may cooperate with *CTCF* to regulate the transcriptional repression of *Cyp2c29*. Therefore, we conducted further ChIP assays and identified a decreasing interaction between *DDX17* and the *Cyp2c29* promoter after knock‐down of *CTCF* or *DDX5* via siRNA transfection in L02 cells (Figures 7A and B). According to the above data, we speculated that *DDX17* interacts with *CTCF* and *DDX5* to function as a co‐TF. Furthermore, the co‐localisation and interaction of *DDX17* with *CTCF* or *DDX5* were identified using IF and COIP assays in 293T cells (Figures S8A–C). We then conducted a series of luciferase reporter assays in L02 cells and 293T cell with the indicated treatment. We found that *CTCF* or *DDX5* could cooperate with *DDX17* to repress the *Cyp2c29* luciferase reporter (Figures 7C, D and S8D–G). In addition, the corresponding mRNA and protein levels of *CYP2C19* and *CYP2C9*,^27^, ^28^ which are reported as the human orthologs of *Cyp2c29* in mice, were altered, which is consistent with the results of the luciferase reporter assays (Figures 7E–L).

Our analysis of RNA‐seq and CUT&Tag, combined with ChIP and luciferase reporter assays, indicated that *DDX17* might cooperate with *CTCF* and *DDX5* in order to regulate the transcriptional repression of *Cyp2c2*9 gene expression in mice and hepatocytes.

### 3.7 DDX17 promotes NASH by repressing cyp2c29 gene expression in mice and hepatocytes

**FIGURE 8:**
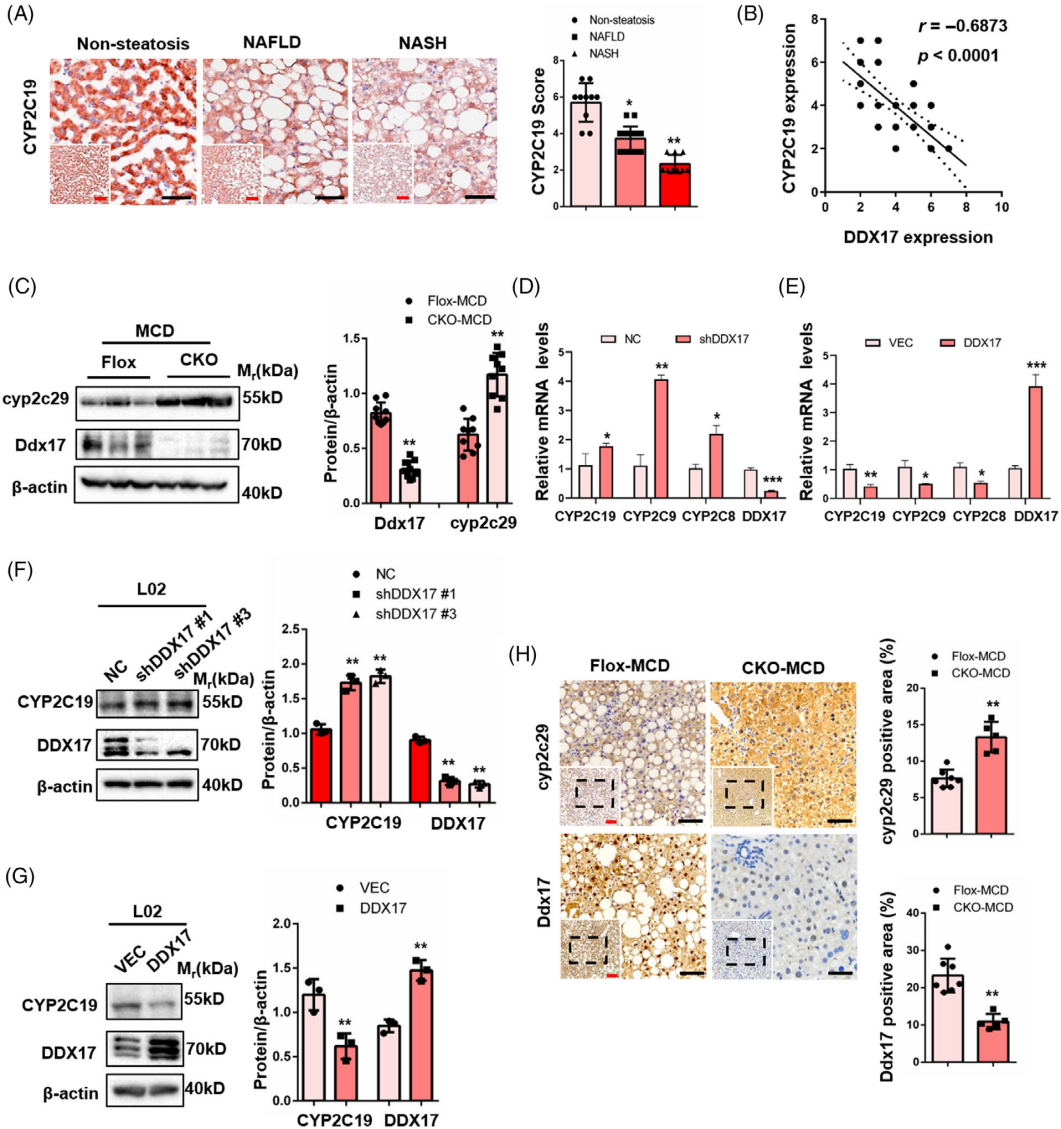
*DDX17* inhibits the *Cyp2c29* gene expression in mice and hepatocytes. (A) Representative immunohistochemistry images (left) and quantification (right) of *CYP2C19* expression in the livers of subjects without steatosis (non‐steatosis; *n* = 10), with NAFLD (NAFLD; *n* = 17) or with NASH (*n* = 9) (*p < .05; **p < .01; red bar, 100 μm; black bar, 50 μm). (B) The scatter plot showed significantly negative correlation between *DDX17* and *CYP2C19* in the livers of individuals without steatosis (non‐steatosis; *n* = 10), with NAFLD (NAFLD; *n* = 17) or with NASH (NASH; *n* = 9). The correlation coefficient and *p* value plotted on the chart. (C) Representative western blots (left) and quantification (right) of DDX17 and cyp2c29 in the livers of MCD‐fed DDX17‐CKO and DDX17‐Flox mice (**p < .01; *n* = 3 western blots). (D) Relative mRNA levels of orthologous genes of *CYP2C19*, *CYP2C9*, *CYP2C8* in L02 hepatocytes with *DDX17* gene overexpression or its control (*p < .05; **p < .01; ***p < .001). (E) Relative mRNA levels of orthologous genes of *CYP2C19*, *CYP2C9*, *CYP2C8* in L02 hepatocytes infected with pLKO.1 control vector or shDDX17 (*p < .05; **p < .01; ***p < .001). (F) Representative western blots (left) and quantification (right) of CYP2C19 and DDX17 in L02 hepatocytes infected with pLKO.1 control vector or shDDX17 (**p < .01; *n* = 3 western blots). (G) Representative western blots (left) and quantification (right) of CYP2C19 and DDX17 in L02 hepatocytes with DDX17 gene overexpression or its control (**p < .01; *n* = 3 western blots). (H) Representative immunohistochemistry images (left) and quantification (right) of Ddx17 expression and cyp2c29 expression in the livers of MCD‐fed DDX17‐Flox (*n* = 7 mice) and DDX17‐CKO mice (*n* = 5 mice) (**p < .01; red and black bar, 100 μm). For (A) and (F), statistical analysis was carried out by one‐way ANOVA. For (C)–(E) and (H), statistical analysis was carried out by two‐tailed Student's t test. All data are shown as the mean ± SD.

Based on the abovementioned results, our next objective was to authenticate the mechanism through which DDX17 governs the expression of the *cyp2c29* gene in both human and mouse liver tissues. We first verified that the protein and mRNA expression of *cyp2c29* were considerably down‐regulate with the progression of NASH in the liver of HFD‐ or MCD‐induced mice and ob/ob mice (Figures S9A–E). This observation aligns with prior research findings.^29^, ^30^ We conducted additional investigations into its expression in the livers of individuals who do not have steatosis, as well as in patients with NAFLD and NASH. We found that hepatic *CYP2C19* (the human ortholog of *Cyp2c29*) protein levels were notably reduced in the liver of individuals with NAFLD or NASH compared with those with non‐steatosis livers (Figure 8A). We also observed a notable negative correlation between *DDX17* and *CYP2C19* in the livers of patients (Figure 8B). Furthermore, we identified a higher *Cyp2c29* protein and mRNA expression in the liver of *DDX17*‐CKO mice than in Flox mice after MCD administration (Figures 8C and 5F). In line with the outcomes observed in vivo, *CYP2C19/C9/C8* (the human orthologs of *Cyp2c29*) mRNA and protein levels were increased in the L02‐shDDX17 group but markedly decreased in the L02‐DDX17 group after PAOA treatment (Figures 8D–G). In addition, we detected a higher expression of *Cyp2c29* in the liver of DDX17‐CKO mice than in Flox mice after MCD administration (Figure 8H). Subsequently, we also found that *Cyp2c29* was down‐regulated and demonstrated a significant negative relationship with *DDX17* in the livers of HFD‐ or MCD‐diet‐fed mice over time (Figures S9F–I). Meanwhile, we validated that *Cyp2c29* deficiency promotes the lipid accumulation in L02 cells (Figures S10A–D). These results suggest that *DDX17* may promote NASH progression by transcriptionally repressing *Cyp2c29* gene expression.

### 3.8 *DDX17* alters lipid composition in murine NASH

**FIGURE 9:**
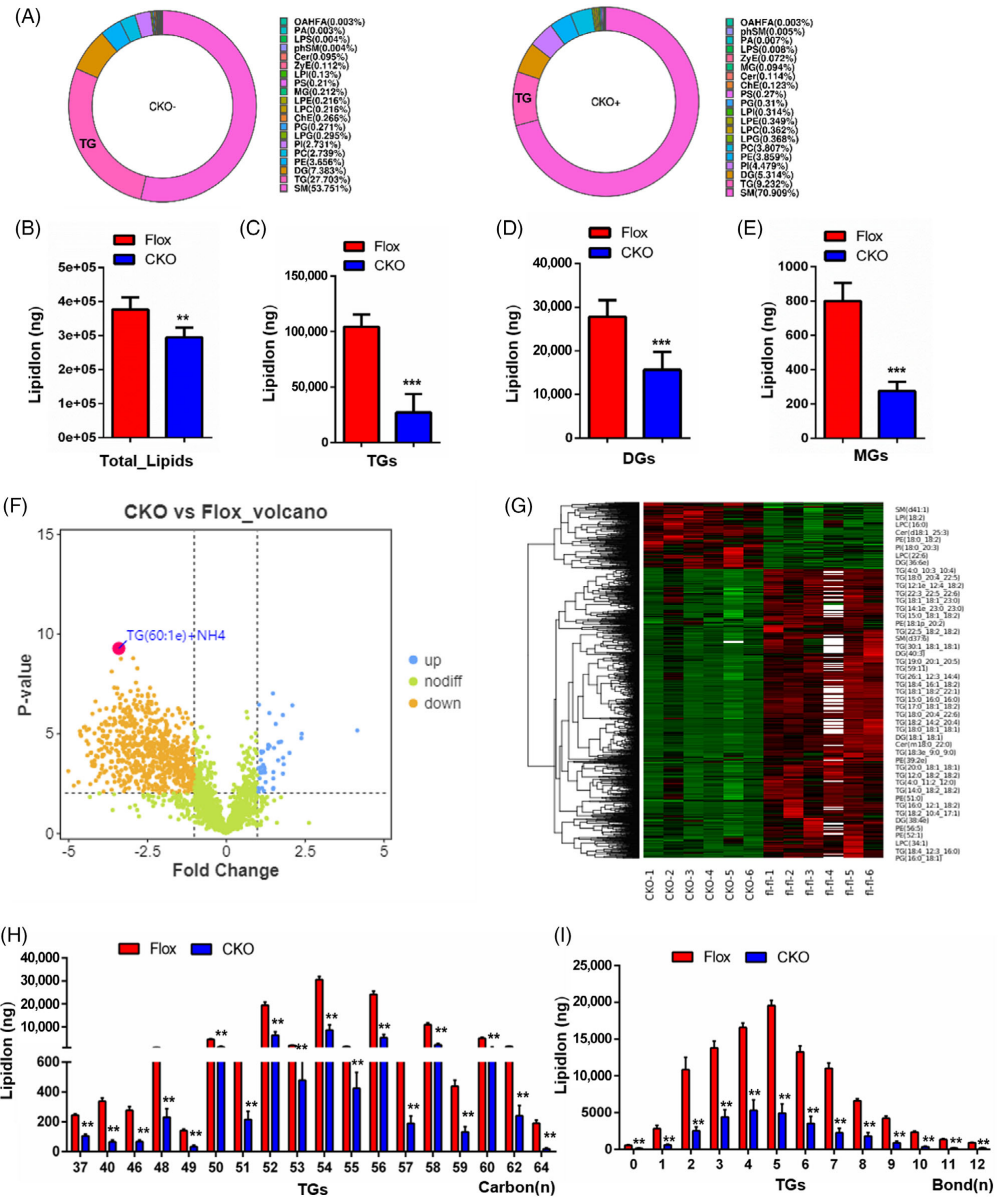
*DDX17* alter the lipid composition primarily by metabolising the glycerides in murine NASH. (A) The composition of lipid subclass in the livers of DDX17‐CKO mice and their corresponding *DDX17*‐Flox controls after MCD consumption were shown in the ring diagram respectively. (B–E) The lipid content of total lipids, TAGs, DAGs and MAGs between the indicated group. (**p < .01; ***p < .001). (F) The volcano plot shows the different lipids between the indicated group. (G) The hierarchical clustering of the indicated group was conducted using the expression of lipid with significant difference (VIP > 1, *p* value < .05) and shown in the heatmap. (H and I) The lipid chain length and saturation analysis of TAGs between the indicated group. The horizontal axis represents lipid molecules of different carbon chain lengths (H) and bond numbers (I), and the vertical axis represents the amount of lipid molecules (**p < .01; n.s., not significant). In all statistical plots, data are statistically analysed using a two‐tailed Student's *t*‐test and expressed as the mean ± SD.

As we know, metabolic dysfunction such as hepatic steatosis is regarded as a significant initial stage in the development of NASH. Lipid accumulation and composition not only constitute a first hit in the progress, but inappropriate lipid metabolism might also drive key further steps such as inflammation and fibrosis in NASH.^31^ *Cyp2c29* has been demonstrated to contribute to fatty acid metabolism and inflammatory response in fatty liver.^32^, ^33^ In order to further investigate the effects of DDX17 in lipid metabolism in NASH, we performed an absolute quantitative lipidomics analysis in liver tissues from *DDX17*‐CKO mice and *DDX17*‐Flox mice after MCD administration. A total of 20 lipid classes and 1811 lipid species were quantified, including various glycerolipid classes such as monoradylglycerols (MGs), diradylglycerols (DGs) and triradylglycerols (TGs), along with sphingolipid classes like ceramide and sphingomyelin (Figure S11A). The lipid subgroup composition and dynamic distribution range of lipid content were analysed in *DDX17*‐CKO and *DDX17*‐Flox mice after MCD administration, as shown in Figures 9A and S11B. Furthermore, lipid content was detected and analysed at a subgroup‐ and molecular level, along with the total lipid content. We found that the total lipids and the main compartment involved glycerolipids (primarily TGs) with considerable amounts of MGs and DGs, which were markedly different between the two groups (Figures 9B–E and S11C–F). Based on the univariate analysis, all detected lipid molecules were analysed. The outcomes were graphically represented through a volcano plot, as illustrated in Figure 9F. Additionally, we applied each sample to hierarchical clustering analysis using the lipids with significant differences (VIP > 1, *p* value < .05) (Figure 9G). These lipid molecules are listed in Additional files 3. Lipid chain and saturation analyses of TGs, DGs and MGs were also conducted between the two groups (Figures 9H–I and S11G–J). It is well known that the lipid chain length and saturation of lipid play a role in regulating the biological functions of the cell, such as unfolded protein response^34^, ^35^ and other membrane signals.^36^, ^37^ Furthermore, lipid composition may influence the extracellular environment or cells such as macrophages.^38^ Collectively, our lipidomic analysis revealed the effect of *DDX17* on liver lipid metabolism in response to MCD feeding, which may support the function of *DDX17* in fatty acid metabolism mediated by *Cyp2c29*.

### 3.9 *DDX17* promotes the progression of liver inflammation and liver fibrosis in murine NASH model

**FIGURE 10:**
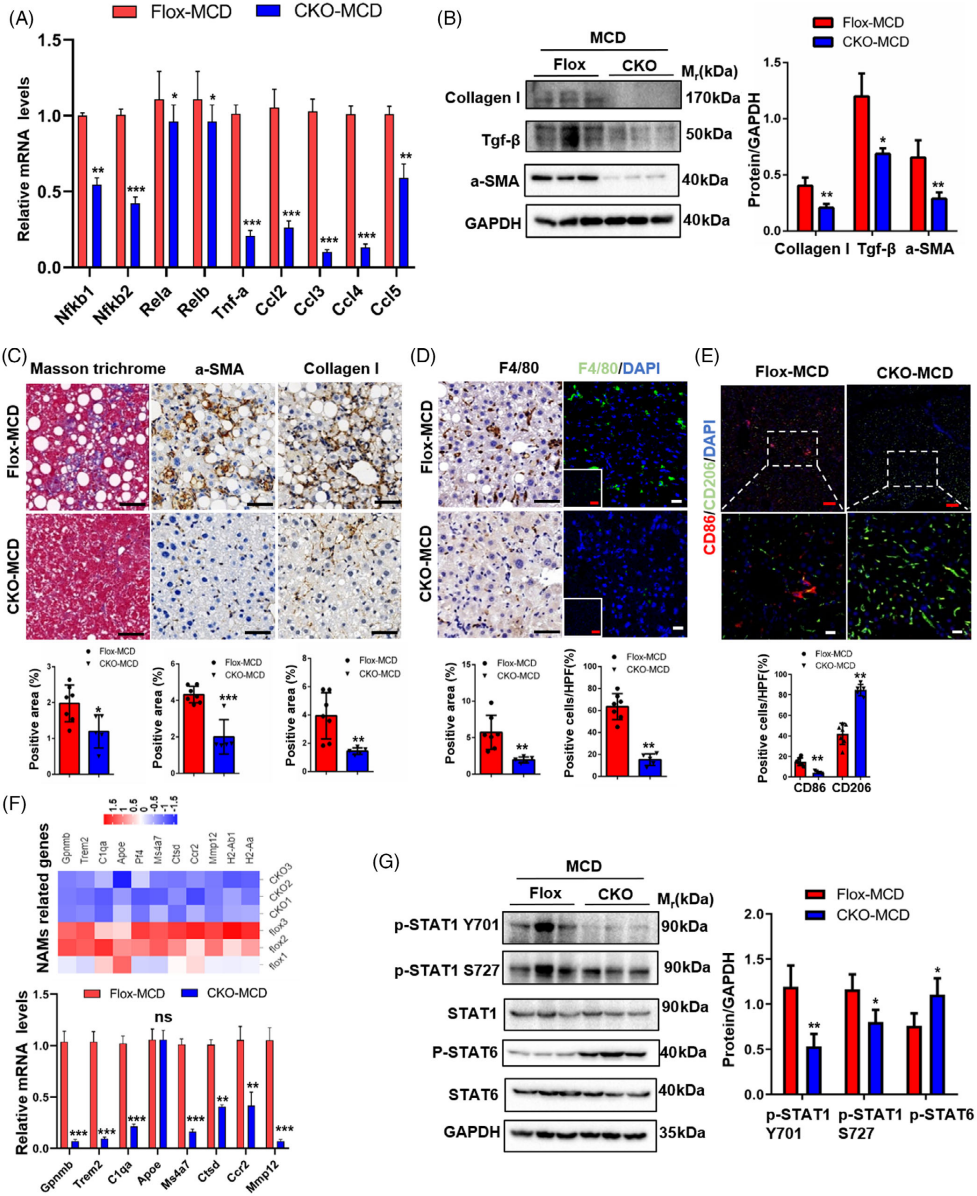
*DDX17* may promote the activation of M1 macrophages and subsequent liver inflammation and liver fibrosis. (A) Relative mRNA levels of inflammation‐related genes in the livers of MCD‐fed *DDX17*‐CKO and *DDX17*‐Flox (**p < .01, ***p < .01). (B) Representative western blots (left) and quantification (right) of fibrotic markers, such as, Collagen I, TGF‐β, a‐SMA, in the livers of MCD‐fed *DDX17*‐CKO and *DDX17*‐Flox (*p < .05; **p < .01; *n* = 3 western blots). (C) Masson trichrome(left), a‐SMA (middle), Collagen (right) staining of liver sections in MCD‐fed *DDX17*‐Flox group (*n* = 7 mice) and *DDX17*‐CKO group (*n* = 5 mice). The positive areas were analysed and quantified (bottom) (*p < .05; **p < .01; ***p < .001; scale bar, 50 μm). (D) Representative immunohistochemistry and immunofluorescence images (left) and quantification (right) of F4/80 expression in the livers of MCD‐fed *DDX17*‐CKO (*n* = 5 mice) and *DDX17*‐Flox (*n* = 7 mice) group (**p < .01; black bar, 100 μm; red bar, 100 μm; white bar, 20 μm). (E) Representative immunofluorescence images of CD86+ (red), CD206+ (green) and DAPI (blue) in liver sections from MCD‐fed *DDX17*‐CKO (*n* = 5 mice) and *DDX17*‐Flox (*n* = 7 mice) group. (**p < .01; red bar, 100 μm; white bar 20 μm). (F) Heatmap analysis of NAMs (NASH‐associated macrophages) genes between *DDX17*‐Flox and *DDX17*‐CKO group. Relative mRNA levels of NAMs genes in the livers of MCD‐fed *DDX17*‐CKO and *DDX17*‐Flox. (*p < .05, **p < .01, ***p < .01). (G) Representative western blots (left) and quantification (right) of p‐STAT1, STAT1, p‐STAT6 and STAT6 in the livers of MCD‐fed *DDX17*‐CKO and *DDX17*‐Flox (*p < .05; **p < .01; *n* = 3 western blots). In all statistical plots, data are statistically analysed using the two‐tailed Student's *t*‐test and expressed as the mean ± SD.

To detect inflammation activation and fibrosis in our murine NASH model, inflammatory and fibrosis‐related genes were further validated using RT‐PCR (Figures 10A and S12A–C). Fibrotic markers, such as *Collagen I*, transforming growth factor beta (*tgf‐β*) and α‐smooth muscle actin (*α‐SMA*), were detected in liver tissues from *DDX17*‐Flox and *DDX17*‐CKO mice groups (Figure 10B). Furthermore, we found that a‐SMA and Collagen, as well as MT, PSR and Sirius red‐stained areas were significantly down‐regulated in the *DDX17*‐CKO mice group in comparison with the *DDX17*‐Flox mice group (Figures 10C and S12D).

The above RNA‐seq analysis suggests that immune response, cytokine production and extracellular matrix organisation may participate in inflammation progress and fibrosis, respectively. Therefore, using the IHC/multiplex IF (IHC/mIF), we assessed the expression of F4/80 (a marker for macrophages), CD86 (a marker for M1 macrophages) and CD206 (a marker for M2 macrophages) in the two experimental groups (Figures 10D and E). Differential quantification revealed that F4/80 and CD86 were dramatically decreased in *DDX17*‐CKO mice compared with *DDX17*‐Flox mice. Recent, studies^39^ on macrophages in NASH progression have identified NASH‐associated macrophages (NAMs), which notably possess a distinct array of molecular markers, including *Gpnmb, Trem2, C1qa* and *Apoe*, which were identified via our analysis of bulk RNA‐seq and validated by RT‐PCR (Figure 10F). Furthermore, our discovery revealed a substantial down‐regulation in the protein levels of p‐STAT1 and p‐STAT6 levels were significantly up‐regulated in the liver tissues of MCD‐fed *DDX17*‐CKO mice when compared with *DDX17*‐Flox mice (Figure 10G). Relative mRNA levels (Figure S12E) of pro‐inflammatory cytokines (*Tnfa, Il6, Il1b* and *Cxcl10*) and serum ELISA levels of pro‐inflammatory cytokines *IL‐6* and *TNF‐a* (Figures S12F and S12G) were also significantly inhibited in the livers of MCD‐fed *DDX17*‐CKO mice when compared with *DDX17*‐Flox mice.^40^ What is more, we validated that the levels of *DDX17* in AML12 shown no effect on RAW264.7 (Figures S13A and S13B). By integrating RNA‐seq and lipidome analyses, our hypothesis suggests that DDX17 could potentially be involved in governing lipid metabolism and M1 macrophage activation in our murine NASH model.

Overall, we identified that *DDX17* depletion alleviated MCD‐induced NASH, and it might play a role mechanistically in the progression of inflammation and fibrosis through the regulation of lipid metabolism and the activation of M1 macrophages.

### 3.10 *Cyp2c29* mediates the function of *DDX17* in lipid accumulation and macrophage activation

**FIGURE 11:**
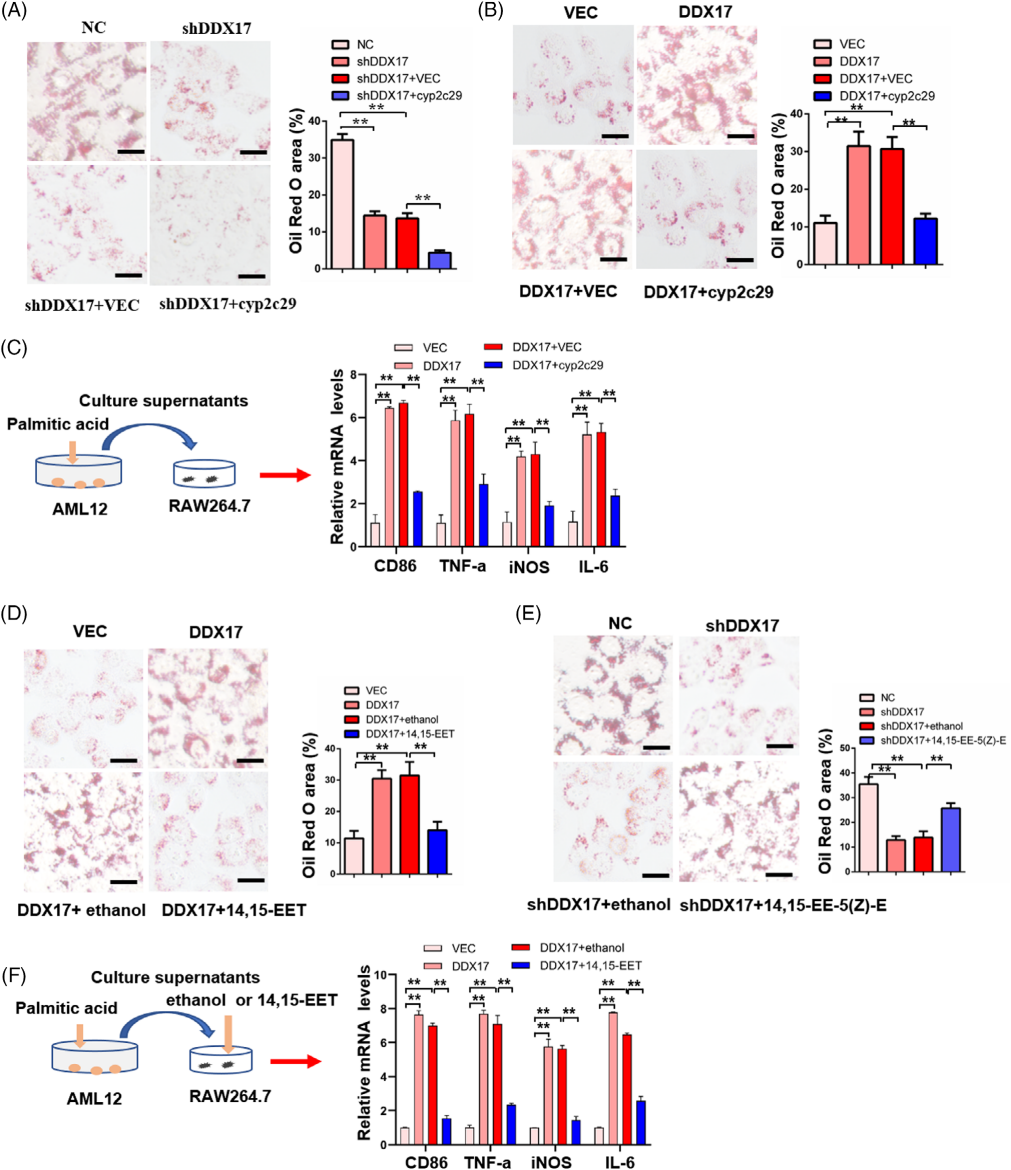
*Cyp2c29* mediates the function of *DDX17* in lipid accumulation and activation of M1 macrophages. (A and B) Representative images (left) and quantitative results (right) of Oil Red O‐stained indicted L02 hepatocytes followed by PAOA (PA, 0.25 mM; OA, 0.5 mM) treatment for 12 h (**p < .01; scale bar, 25 μm; *n* = 3 independent experiments). (C) mRNA levels of CD86, TNF‐a, iNOS and IL‐6 in RAW264.7cells followed by indicted AML12 cells culture supernatant treatment for 12 h (**p < .01; *n* = 3 independent experiments). (D and E) Representative images (left) and quantitative results (right) of Oil Red O‐stained indicted L02 hepatocytes followed by PAOA (PA, 0.25 mM; OA, 0.5 mM) treatment for 12 h (**p < .01; scale bar, 25 μm; *n* = 3 independent experiments). (F) mRNA levels of *CD86*, *TNF‐a*, *iNOS* and *IL‐6* in RAW264.7cells followed by indicted AML12 cells culture supernatant treatment for 12 h (**p < .01; *n* = 3 independent experiments); ethanol used as the solvent control of 14,15‐EET. Data are analysed by one‐way ANOVA and shown as the mean ± SD.

Next, we explored whether *DDX17* promotes lipid accumulation and inflammatory responses by regulating the expression of *Cyp2c29*. We found that overexpression of *Cyp2c29* diminished lipid accumulation induced by PAOA in both the L02‐shDDX17 and L02‐DDX17 groups when compared with their respective controls (Figures 11A and B). Furthermore, we also found that the mRNA levels of activated M1 macrophage markers including *CD86*, *TNF‐α*, *iNOS* and *IL‐6* were markedly decreased in the AML12‐DDX17 group after overexpression of *Cyp2c29* (Figure 11C).

Previous studies have reported that epoxyeicosatrienoic acids (EETs), especially 14,15‐EET, which is the main metabolite of AA and is metabolised by *Cyp2c29*, play roles in anti‐inflammation and anti‐fibrosis in different diseases.^32^, ^33^, ^41^, ^42^ First, we detected hepatic levels of 14,15‐EET (Figure S13C) were markedly increased in the livers of MCD‐fed *DDX17*‐CKO mice when compared with *DDX17*‐Flox mice. Therefore, we tested whether *Cyp2c29* metabolites and 14,15‐EET alleviates lipid accumulation. We found that lipid accumulation induced by PAOA in the L02‐DDX17 group was decreased after supplementation with 14,15‐EET; however, was increased by 14,15‐EET antagonist (14,15‐EE‐5(Z)‐E) treatment in the L02‐shDDX17 group (Figures 11D and E). Furthermore, the mRNA levels of activated M1 macrophage markers including *CD86, TNF‐α, iNOS* and *IL‐6* were also alleviated by 14,15‐EET supplementation, but aggravated by the 14,15‐EET antagonist (14,15‐EE‐5(Z)‐E) treatment (Figure 11F). These results suggested that *Cyp2c29* and its metabolites 14,15‐EET might mediate the function of DDX17 in lipid accumulation in hepatocytes and macrophage activation in order to promotes the NASH progression.

### 3.11 Clinical relationship between *DDX17*, *Cyp2c29* and liver steatosis and fibrosis

The above results indicate the up‐regulated expression of *DDX17* and the down‐regulated expression of *Cyp2c29* in the livers of human individuals and murine NASH models, when compared with their corresponding controls. Next, we ascertained the clinical impact of *DDX17* and *Cyp2c29* in patients with NASH. We found that DDX17 was substantially and positively correlated with steatosis (rho: 0.710, *p* < .001), lobular inflammation (rho: 0.515, *p* = .001), ballooning (rho: 0.625, *p* < .001) and fibrosis (rho: 0.418, *p* < .001), which are the major histological features of NASH (Table 1). Conversely, *Cyp2c29* expression was substantially and negatively correlated with steatosis (rho: −0.794, *p* < .001), lobular inflammation (rho: −0.666, *p* < .001), ballooning (rho: −0.695, *p* < .001) and fibrosis (rho: −0.470, *p* = .004) (Table 2).

**TABLE 1:** Correlations with *DDX17* in patients.

	DDX17	
	Rho	*p* Value
Age	−0.094	.587
BMI	0.199	.244
ALT	0.329	.05
AST	0.283	.094
Cholesterol	−0.182	.288
Triglycerides	0.134	.437
HDL	−0.055	.749
LDL	0.037	.833
FBG	0.098	.568
Steatosis	0.710	<.001
Lobular inflammation	0.515	.001
Ballooning	0.625	<.001
Fibrosis	0.418	.011

**TABLE 2:** Correlations with *CYP2C19* in patients.

	CYP2C19	
	Rho	*p* Value
Age	0.137	.425
BMI	−0.458	.005
ALT	−0.240	.158
AST	−0.243	.154
Cholesterol	−0.181	.29
Triglycerides	−0.292	.084
HDL	0.113	.512
LDL	−0.389	.019
FBG	−0.29	.086
Steatosis	−0.794	<.001
Lobular inflammation	−0.666	<.001
Ballooning	−0.695	<.001
Fibrosis	−0.470	.004

**FIGURE 12:**
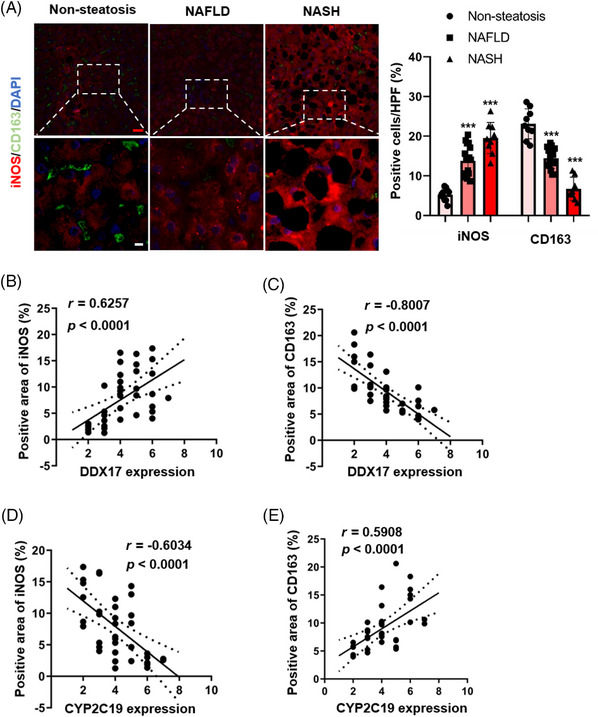
Clinical relationship between *DDX17*, *Cyp2c29* and liver steatosis and fibrosis (A) Representative immunofluorescence images of iNOS+ (red), CD163+ (green) and DAPI (blue) in liver sections from individuals without steatosis (non‐steatosis; *n* = 10), with NAFLD (NAFLD; *n* = 17) or with NASH (*n* = 9) (***p < .001; red bar, 100 μm, white bar 20 μm). (B) The scatter plot showed significantly positive correlation between *DDX17* and *iNOS*, the marker of M1 macrophages in the livers of individuals without steatosis (non‐steatosis; *n* = 10), with NAFLD (NAFLD; *n* = 17) or with NASH (*n* = 9). (C) The scatter plot showed significantly negative correlation between *DDX17* and *CD163*, the marker of M2 macrophages in the livers of individuals without steatosis (non‐steatosis; *n* = 10), with NAFLD (NAFLD; *n* = 17) or with NASH (*n* = 9). (D) The scatter plot showed significantly negative correlation between *CYP2C19* and *iNOS*, the marker of M1 macrophages in the livers of individuals without steatosis (non‐steatosis; *n* = 10), with NAFLD (NAFLD; *n* = 17) or with NASH (*n* = 9). (E) The scatter plot showed significantly positive correlation between *CYP2C19* and *CD163*, the marker of M2 macrophages in the livers of individuals without steatosis (non‐steatosis; *n* = 10), with NAFLD (NAFLD; *n* = 17) or with NASH (*n* = 9). The correlation coefficient and p value plotted on the chart.

Following the findings outlined above, we proceeded to investigate the correlation between the activation of M1 macrophages and *DDX17* and *CYP2C19* in the livers of human subjects. The quantification of mIF revealed an elevation in iNOS, a marker associated with M1 macrophages, and a reduction in CD163, a marker for M2 macrophages, within the livers of individuals afflicted with NAFLD or NASH, as opposed to those with non‐steatotic livers (Figure 12A). In addition, *DDX17* was significantly positively correlated with *iNOS* (*r* = 0.6257, *p* < .0001) and negatively correlated with *CD163* (*r* = −0.8007, *p* < .0001), and *CYP2C19* was significantly negatively correlated with *iNOS* (*r* = −0.6034, *p* < .0001) and positively correlated with *CD163* (*r* = 0.5908, *p* < .0001) (Figures 12B–E). These results further proved that *DDX17* plays a role in lipid metabolism, activation of M1 macrophages and subsequent inflammatory responses through the regulation of *Cyp2c29* during the progression of NASH.

## 4 DISCUSSION

In this research, we identified that *DDX17* in hepatocytes is a novel NASH promoter that stimulates lipid accumulation, inflammation and fibrosis in the liver by cooperating with *DDX5* and *CTCF* in order to repress the transcription of *Cyp2c29*, which ultimately diminishes the production of 14,15‐EETs. In vivo studies have shown that hepatocyte‐specific knockout of DDX17 attenuates MCD‐ or HFD‐diet‐induced hepatic steatosis, inflammation and fibrosis. Mechanistically, we found that hepatic *DDX17* affects lipid accumulation by repressing the transcription of *Cyp2c29*, which metabolises AA to several beneficial metabolites such as EETs and specialised proresolving mediators (SPMs).^32^, ^33^, ^41^, ^42^ CUT&Tag analysis, ChIP and luciferase reporter assays were conducted to explore the transcription of *DDX17*, and a series of rescue experiments were performed by supplementing with 14, 15‐EET and its antagonist to confirm the role of *DDX17*/*Cyp2c29*/ 14, 15‐EET pathway in lipid metabolism and inflammation. Hence, our investigation unveils a fresh mechanism, shedding light on the DDX17 targets within the context of NASH, thus offering a promising avenue for NASH treatment.

*DDX17* (p72), and its paralog *DDX5* (p68), which are members of the RNA helicase family, can interact with several components of transcriptional machinery such as RNA polymerase II, histone deacetylases and CBP/p300^5^, ^13^, ^14^ in order to play the roles in transcription. Recently several studies about HCC and few studies about metabolic diseases have been reported.^8^, ^15^, ^16^, ^17^ In our previous work,^18^ we found that *DDX17* was up‐regulated in the livers or HCC tissues of DEN treated C57 mice that had been fed a HFD diet for 8 months compared with mice simply treated with DEN alone. Consequently, our conjecture postulates the role of DDX17 in the progression of both NASH and NASH‐HCC.

In our present study, hepatocyte‐specific knockout of *DDX17* attenuated MCD or HFD‐diet‐induced hepatic steatosis, inflammation and fibrosis. Our analysis of RNA‐seq and CUT&Tag, combined with ChIP and luciferase reporter assays, indicated that *DDX17* transcriptionally represses *Cyp2c29* gene expression by cooperating with *CTCF* and *DDX5*. Using absolute quantitative lipidomics analysis, we found that a hepatocyte‐specific *DDX17* deficiency decreased lipid accumulation and altered lipid composition in the livers of mice after MCD administration. Combined with RNA‐seq analysis and lipidome analysis, we found that *DDX17* may play a role in the regulation of lipid metabolism and M1 macrophage activation in murine NASH models. Furthermore, a global RNA landscape was detected in the livers of MCD‐administered *DDX17*‐CKO mice and *DDX17*‐Flox mice using RNA‐seq. Bulk RNA‐seq revealed the role of *DDX17* in lipid metabolism, inflammation and fibrosis. Furthermore, differential lipid metabolism and macrophage activation‐related genes were analysed and visualised to identify the specific mechanisms. Among them, NAMs signature markers, such as *Gpnmb, Trem2, C1qa and Apoe*,^39^ were also identified via our bulk RNA‐seq and related analysis. This result implies that *DDX17* could potentially influence hepatic macrophage activation and may even promote the progression of NASH‐HCC by remodelling the tumour‐prone liver microenvironment. Moreover, we demonstrated that *DDX17* may promote the activation of M1 macrophages and inhibit the activation of M2 macrophages by causing the metabolic disorders. Recently, Deng et al.^43^ reported that M2 macrophages possess the capacity to suppress stellate cells activation by secreting exosomes containing microRNA‐411‐5p. We found that differential activation of macrophages may promote the advancement of liver inflammation and fibrosis in murine NASH models. These results indicate that pro‐inflammatory genes and fibrotic genes were significantly down‐regulated in the liver tissues of *DDX17*‐CKO mice. Thus, *DDX17* may not only regulate lipid metabolism in hepatocyte, but also indirectly regulate the activation of M1 macrophages and the inhibition of M2 macrophages, as well as subsequent HSCs activation. We combined RNA‐seq analysis in murine NASH liver and CUT&Tag in L02 cells with ChIP and luciferase reporter assays in order to validate the transcriptional regulation of *Cyp2c29* by *DDX17*. Consistent with previous studies,^22^, ^23^, ^24^ our results indicated that DDX17 might cooperate with *DDX5* and *CTCF* to regulate the transcription of *Cyp2c29* by binding the classic CTCF motif “CCCTC” at the promoter of *Cyp2c29*. In our experiments, we cannot exclude the possibility that other TF or RNA polymerase can interact with *DDX17*, *DDX5* and *CTCF* to regulate the transcription of *Cyp2c29*. Therefore, more specific binding modes and epigenetic regulations should be elucidated.

*Cyp2c29*, the mouse ortholog of both *CYP2C9* and *CYP2C19*, belongs to the CYP2C subfamily and cytochromes P450 superfamily (CYP450s).^29^, ^30^ The CYP2C subfamily in humans comprises four isoforms (*CYP2C19, CYP2C9, CYP2C8 and CYP2C18*) that account for approximately 20% of the total liver P450 contents.^44^ They play a critical role in the oxidative metabolism of xenobiotics and endogenous substrates. Recent research findings have indicated that *Cyp2c29* plays a critical role in the metabolism of AA, leading to the formation of bioactive EETs. These compounds have demonstrated significant anti‐inflammatory properties and exhibit protective effects in a range of conditions, encompassing NASH and cardiovascular diseases.^29^, ^30^, ^45^, ^46^ In our current investigation, the mRNA and protein expression of *Cyp2c29* tended to decrease with NAFLD progression, which is consistent with our results in the HFD‐and MCD‐induced NASH models (Figures S7A–H). Collectively, these studies indicate that the inhibition of EET biosynthesis mediated by the hepatic CYP450s, a pivotal inflammatory risk factor in the fatty liver disease, and that the CYP epoxygenase pathway is a primary regulator of the inflammatory response in the progression of NAFLD/NASH. Furthermore, some evidence has indicated that *Cyp2c29* is down‐regulated during human HCC progression and is considered a novel gene involved in liver injury and inflammation.^47^, ^48^, ^49^, ^50^ Based on the above studies, we performed a series of rescue experiments in which overexpression of *Cyp2c29* or supplementation with 14,15‐EET alleviated lipid accumulation and macrophage activation in L02 and AML12 cells. Furthermore, we found negative relationship between *DDX17* and *Cyp2c29* expression in HFD‐ and MCD‐induced murine NASH models and in patients with NASH. Overall, these findings suggest that the down‐regulation of *Cyp2c29* expression by DDX17 and the subsequent low EET levels may trigger lipid metabolism dysfunction and anti‐inflammatory effects and may be a critical pathological consequence of NASH in vivo.

NAFLD is a complex disease with highly heterogeneous factors and clinical manifestations among individuals. The ‘multiple‐hits’ hypothesis involved various risk factors provides a more acceptable interpretation of the pathogenesis of NAFLD. In addition to genetic factors, inflammation, innate immunity, metabolic homeostasis, lipotoxicity and cell death have been found to be related to NASH.^1^, ^3^ Recently, it is reported that DDX17 has a protective role in hepatocytes when confronted with lipid accumulation induced by oleic acid and palmitic acid.^51^ In view of the differences, we considered the following factors. First, our study chosen the normal liver cell line (L02), and a human and mouse hepatic carcinoma cell line (HepG2 and Hep1‐6) were used in the literature, which may be due to the differences in the genetic background and metabolic mode between normal liver cells and tumour cells. In addition, the mechanism of DDX helicase family is very complex in different cell and disease background. DDX helicase regulate the expression of target gene by binding to different TF or chromatin complexes. In view of such a precise regulatory mechanism, it is difficult to accurately simulate the physiological function of DDX helicase by simply performing the experiments in vitro. Based on these factors, we adopt the hepatocyte‐specific *DDX17* knockout mice and two high‐fat mice models to explore the metabolic phenotype and molecular mechanism, which may make up for the shortcomings of cell‐line models. In this study, we demonstrated that *DDX17* promotes lipid accumulation in hepatocytes. The mRNA and protein levels were markedly up‐regulated in the progression of HFD‐ and MCD‐induced NASH in mice. However, there are still some shortcomings, such as the therapeutic effects of *DDX17* in NASH mice and the exclusion of other metabolites of *Cyp2c29*, which remain to be solved. What is more, the MCD diet model, which was chosen in our experiments and further analyses, exists several limitations during to causing the acute weight loss and without the insulin resistance. Therefore, reasonable NASH models should be conducted to validate our results in the future.

In conclusion, our findings revealed a substantial increase in DDX17 expression within the livers of both human subjects and murine models with NASH. Further, hepatic *DDX17* transcriptionally represses *Cyp2c29* gene expression by cooperating with *CTCF* and *DDX5*. The results indicate that DDX17 contributes to the progression of NASH by stimulating the lipid accumulation in hepatocytes and inducing the activation of M1 macrophages as well as promoting a subsequent inflammatory response and fibrosis through the transcriptional repression of *Cyp2c29* in mice. Overall, our study reveals the role of the *DDX17/Cyp2c29*/ 14, 15‐EET pathway in lipid metabolism and subsequent inflammatory response and provides promising targets for the treatment of hepatic steatosis, inflammation and fibrosis.

## AUTHOR CONTRIBUTIONS

*Methodology; investigation; writing original draft*: Deng Ning. *Methodology; investigation; writing review and editing*: Jie Jin. *Methodology; investigation; writing review and editing*: Yuanyuan Fang. *Methodology; formal analysis*: Pengcheng Du. *Methodology; investigation*: Chaoyi Yuan. *Methodology; investigation*: Jin Chen. *Methodology*: Qibo Huang. *Methodology*: Kun Cheng. *Methodology; investigation*: Jie Mo. *Methodology; investigation*: Lei Xu. *Resources*: Hui Guo. *Review and editing*: Mia Jiming Yang. *Writing—review and editing*: Xiaoping Chen. *Methodology; investigation; writing—review and editing*: Huifang Liang. *Methodology; investigation; writing—review and editing*: Bixiang Zhang. *Conceptualisation; writing—original draft; writing—review and editing; funding acquisition; resources; supervision*: Wanguang Zhang. All authors read and approved the final manuscript.

## CONFLICT OF INTEREST STATEMENT

The authors declare that they have no conflict of interest.

## ETHICS STATEMENT AND CONSENT TO PARTICIPATE

All animal experiments were conducted in accordance with the protocols were approved by the Tongji Hospital Animal Care and Use Committee (TJH‐202101106). All procedures involving human samples were approved by the Tongji Hospital of Huazhong University of Science and Technology Review Board, Wuhan, China and were consistent with the principles outlined in the Declaration of Helsinki. Informed consent was written by the subjects or immediate families of the liver donors.

## Supporting information

Supplementary Figures

Supplementary Materials

Additional Files

## Data Availability

The data presented in this study are available on reasonable request from the corresponding author.
