# Supplementary figures and images for: DEAD‐Box Helicase 17 exacerbates non‐alcoholic steatohepatitis via transcriptional repression of cyp2c29, inducing hepatic lipid metabolism disorder and eliciting the activation of M1 macrophages
Source: Clin Transl Med. 2024 Feb 1;14(2):e1529. doi: 10.1002/ctm2.1529 (PMC10835191; doi:10.1002/ctm2.1529)

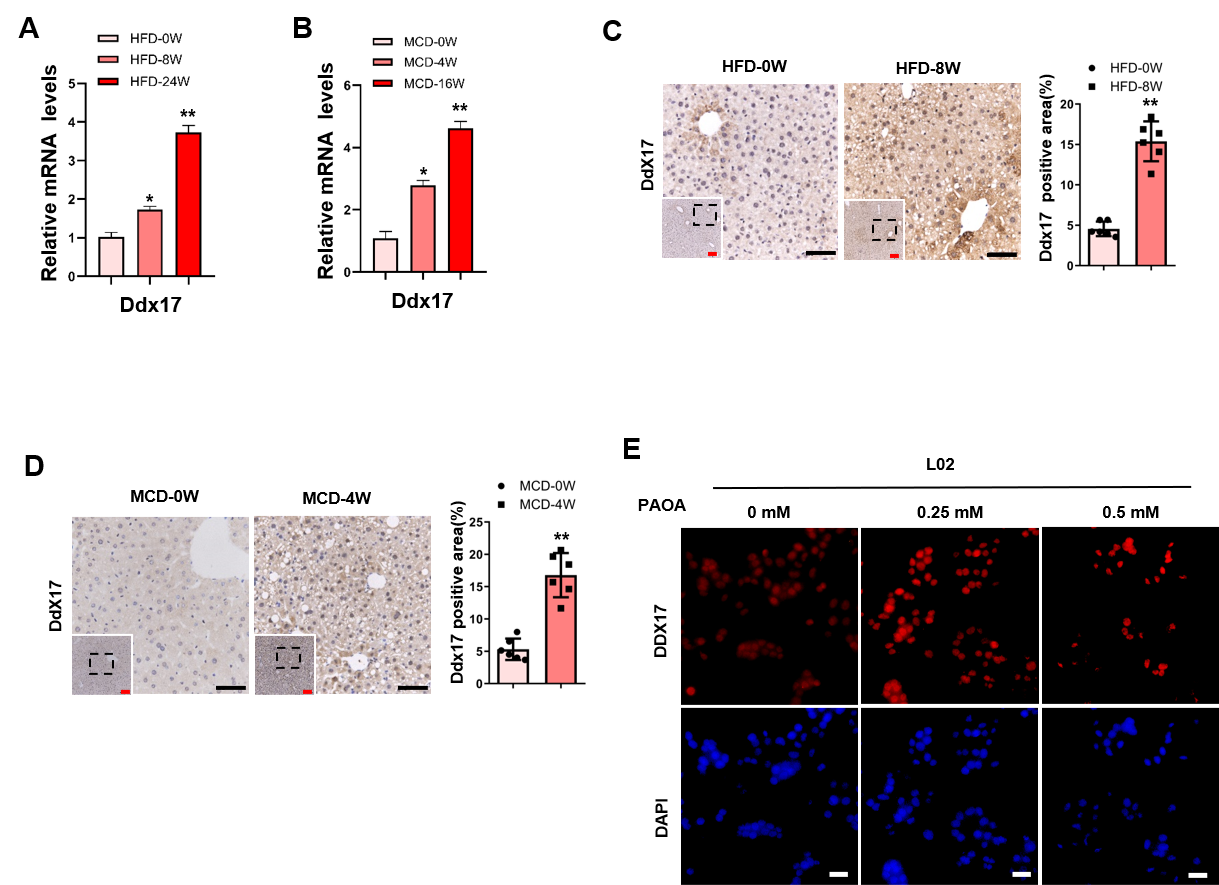

Supplement: Supplementary file 1 — Supplementary Figures [file CTM2-14-e1529-s003.zip › ctm/ctm21529-sup-0001-figureS1.png]

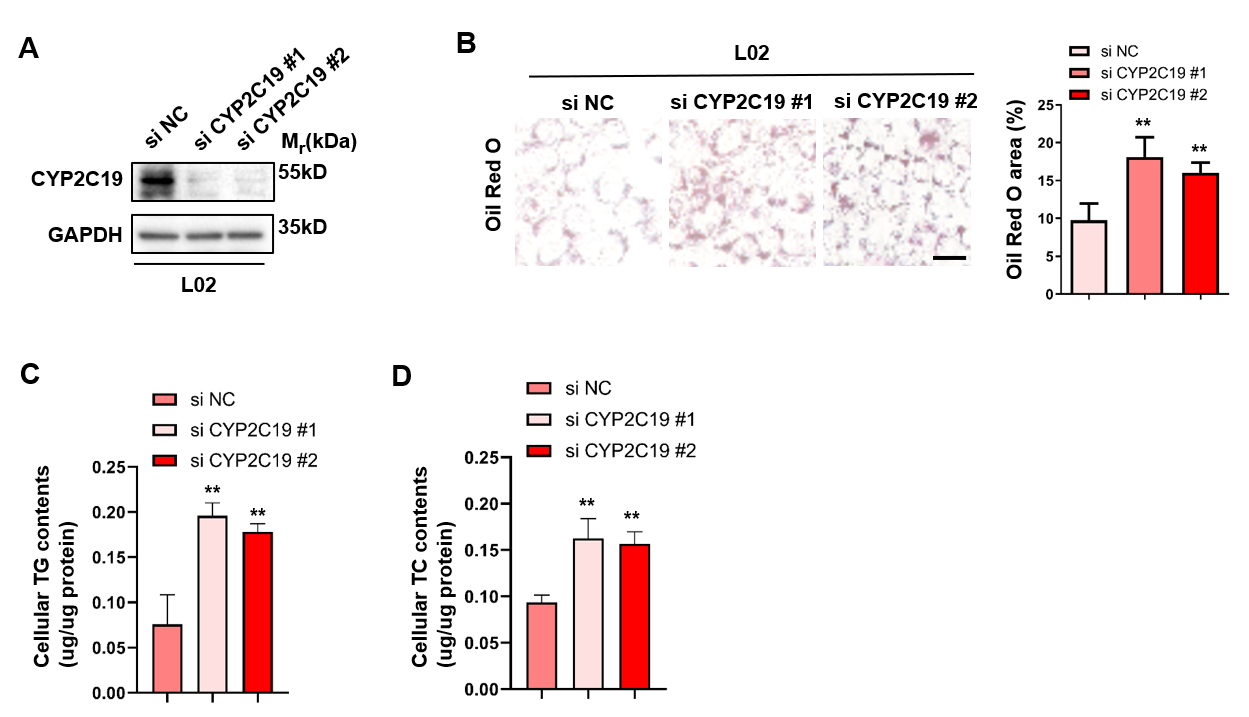

Supplement: Supplementary file 1 — Supplementary Figures [file CTM2-14-e1529-s003.zip › ctm/ctm21529-sup-0001-figureS10.png]

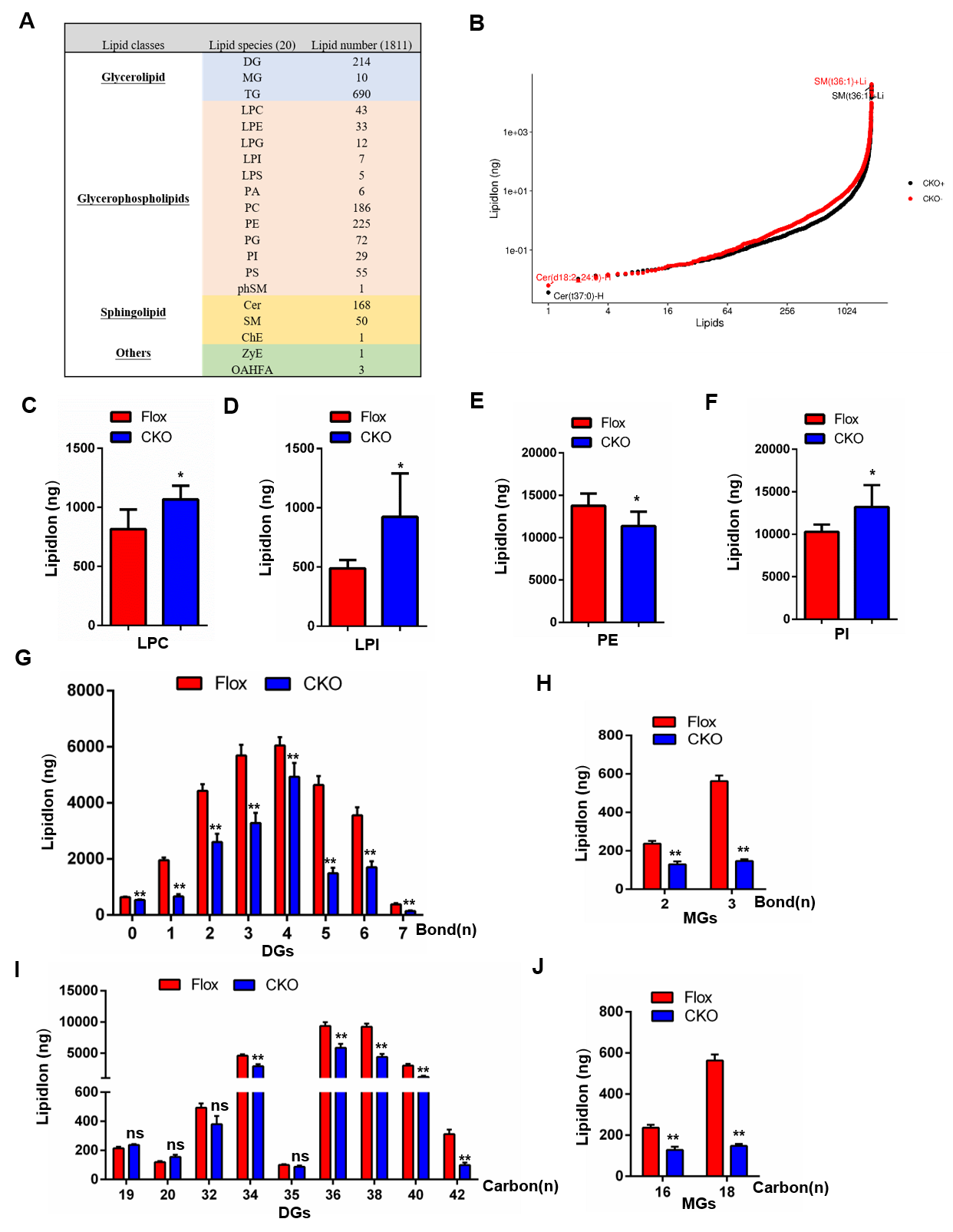

Supplement: Supplementary file 1 — Supplementary Figures [file CTM2-14-e1529-s003.zip › ctm/ctm21529-sup-0001-figureS11.png]

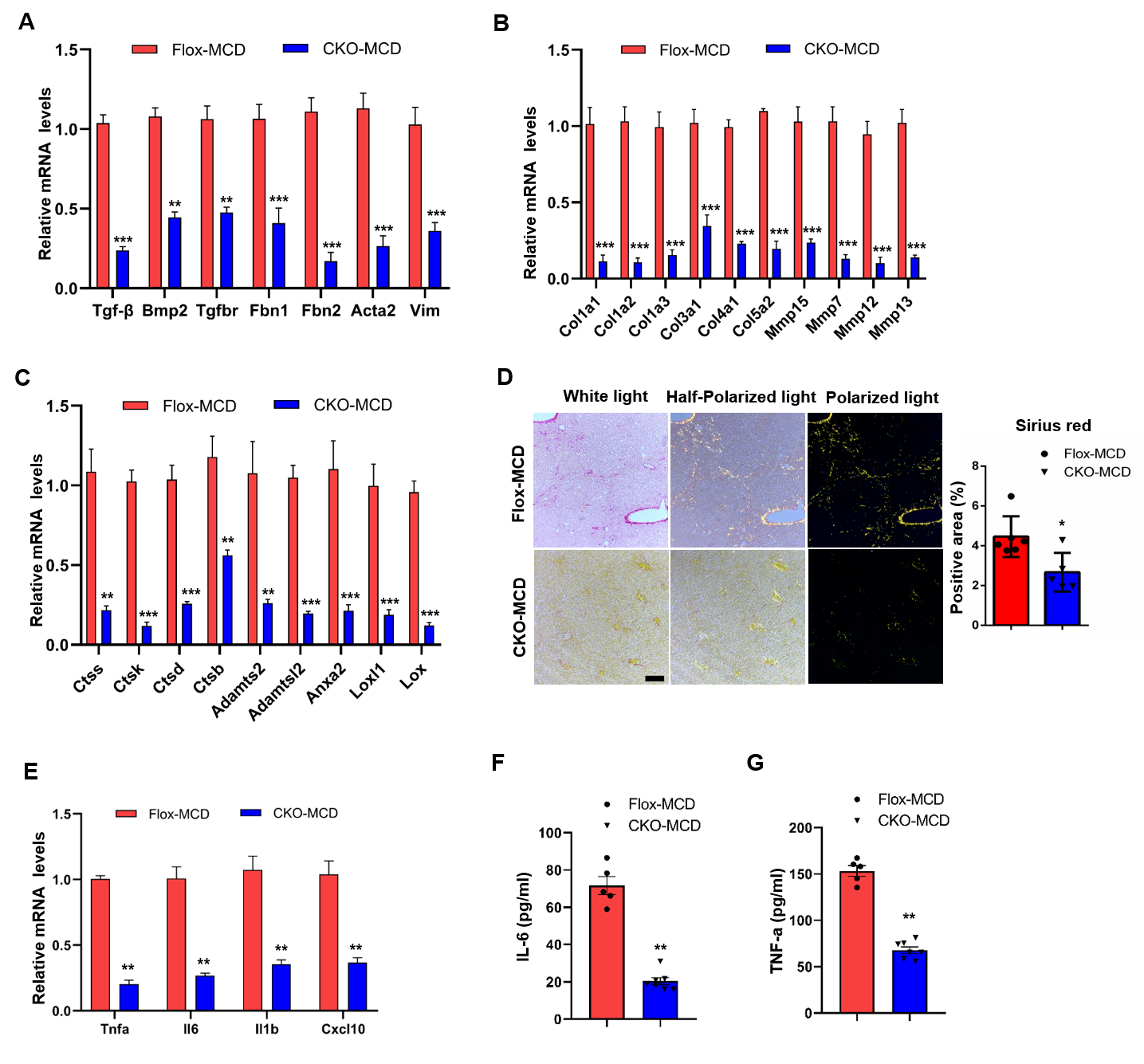

Supplement: Supplementary file 1 — Supplementary Figures [file CTM2-14-e1529-s003.zip › ctm/ctm21529-sup-0001-figureS12.png]

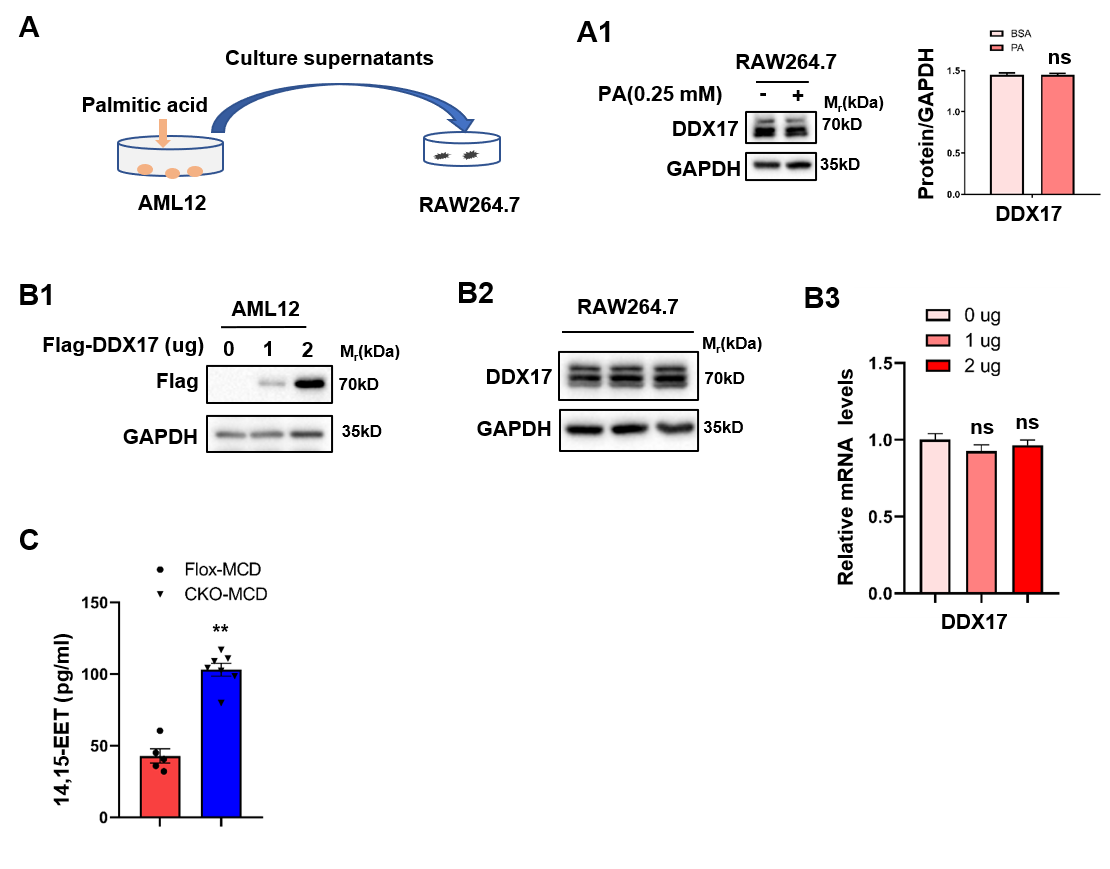

Supplement: Supplementary file 1 — Supplementary Figures [file CTM2-14-e1529-s003.zip › ctm/ctm21529-sup-0001-figureS13.png]

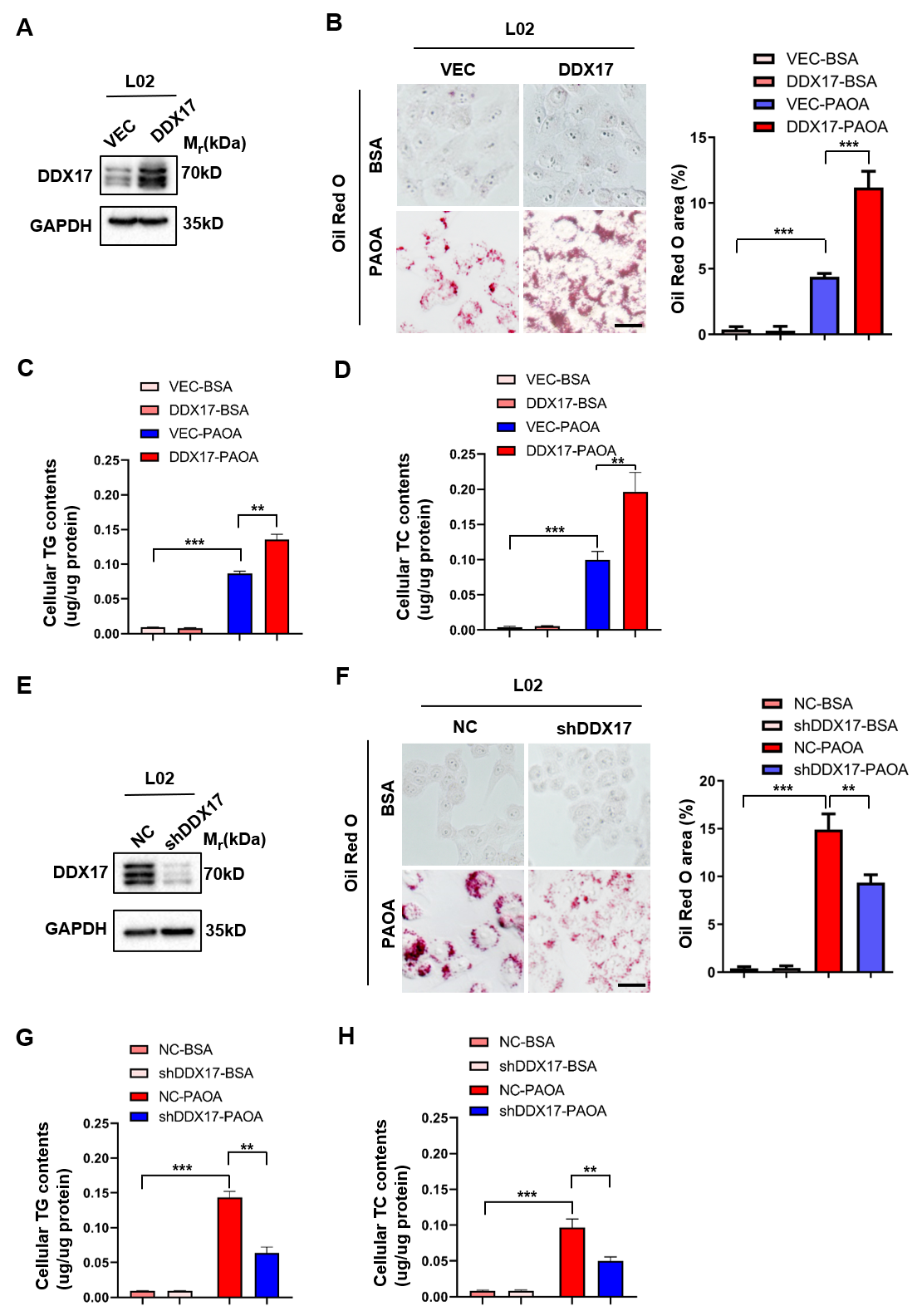

Supplement: Supplementary file 1 — Supplementary Figures [file CTM2-14-e1529-s003.zip › ctm/ctm21529-sup-0001-figureS2.png]

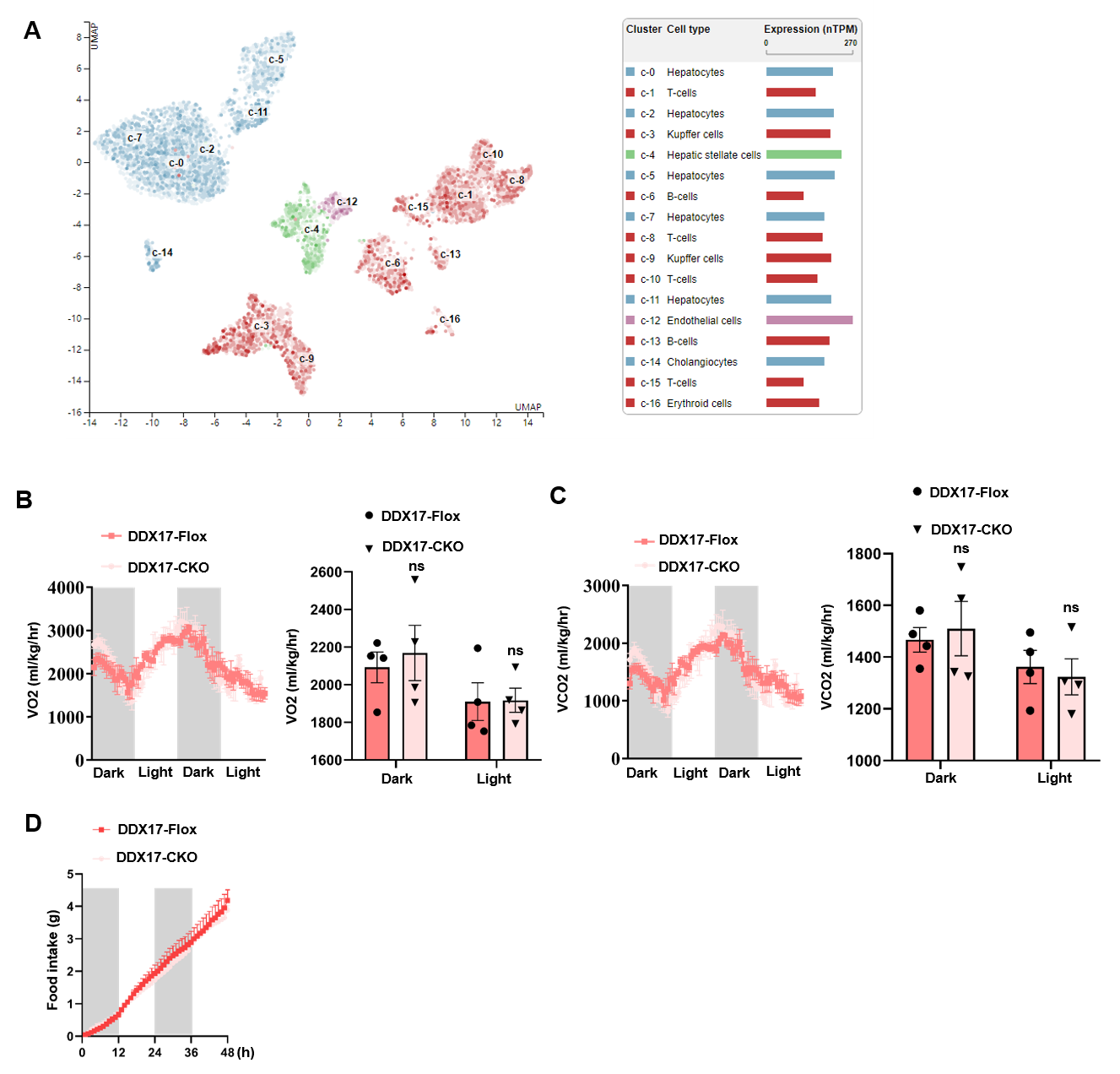

Supplement: Supplementary file 1 — Supplementary Figures [file CTM2-14-e1529-s003.zip › ctm/ctm21529-sup-0001-figureS3.png]

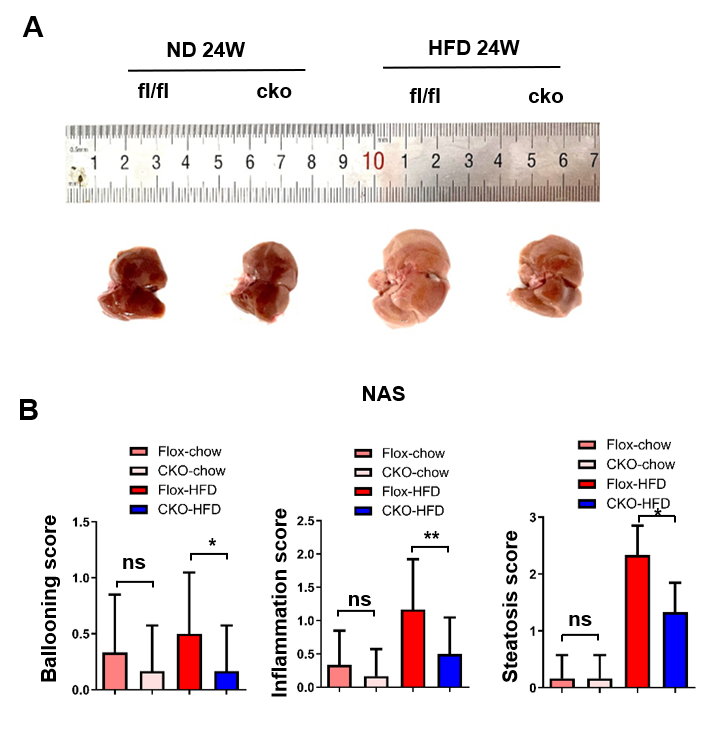

Supplement: Supplementary file 1 — Supplementary Figures [file CTM2-14-e1529-s003.zip › ctm/ctm21529-sup-0001-figureS4.png]

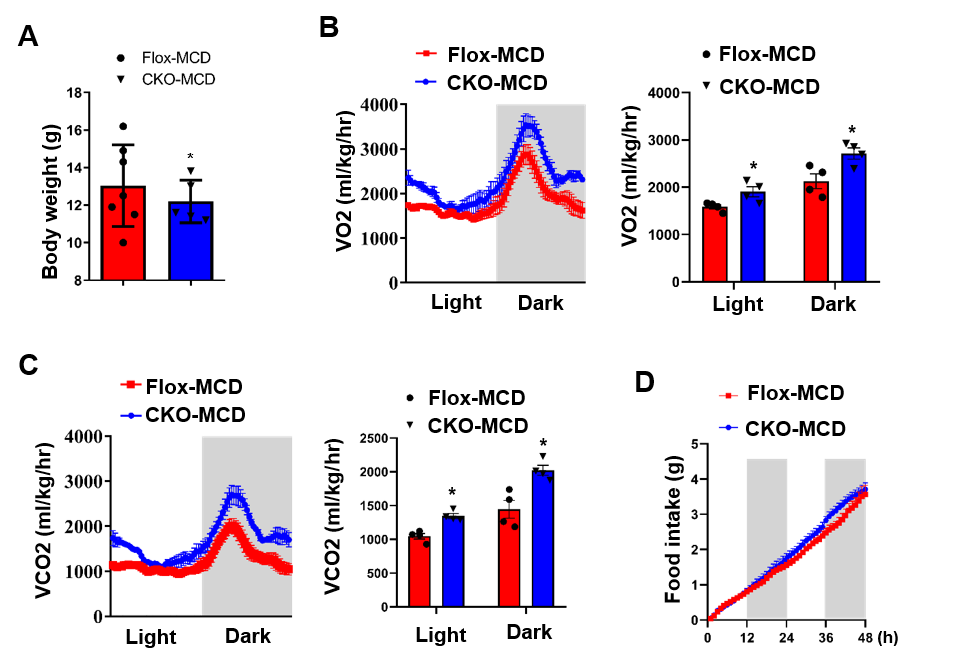

Supplement: Supplementary file 1 — Supplementary Figures [file CTM2-14-e1529-s003.zip › ctm/ctm21529-sup-0001-figureS5.png]

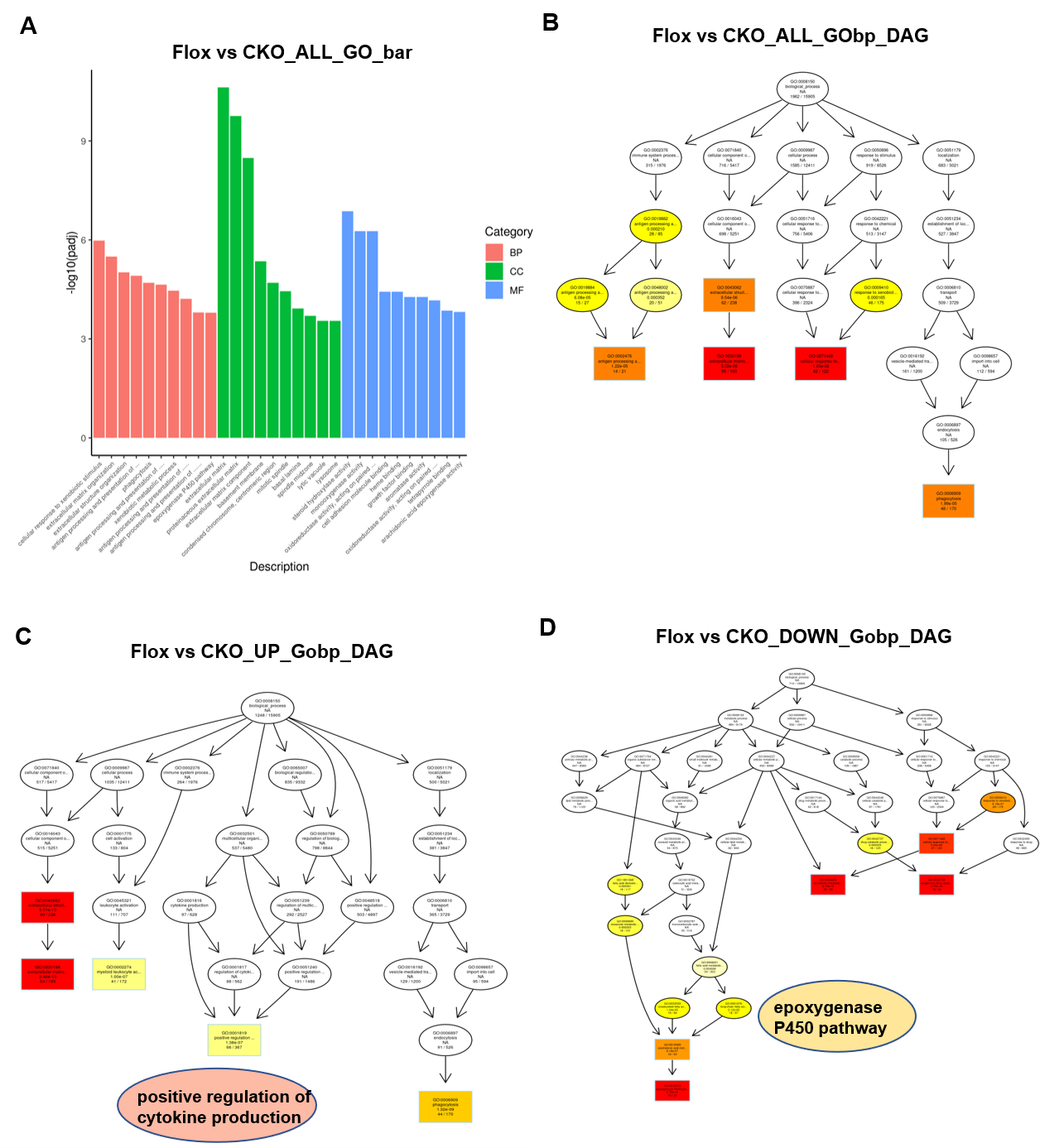

Supplement: Supplementary file 1 — Supplementary Figures [file CTM2-14-e1529-s003.zip › ctm/ctm21529-sup-0001-figureS6.png]

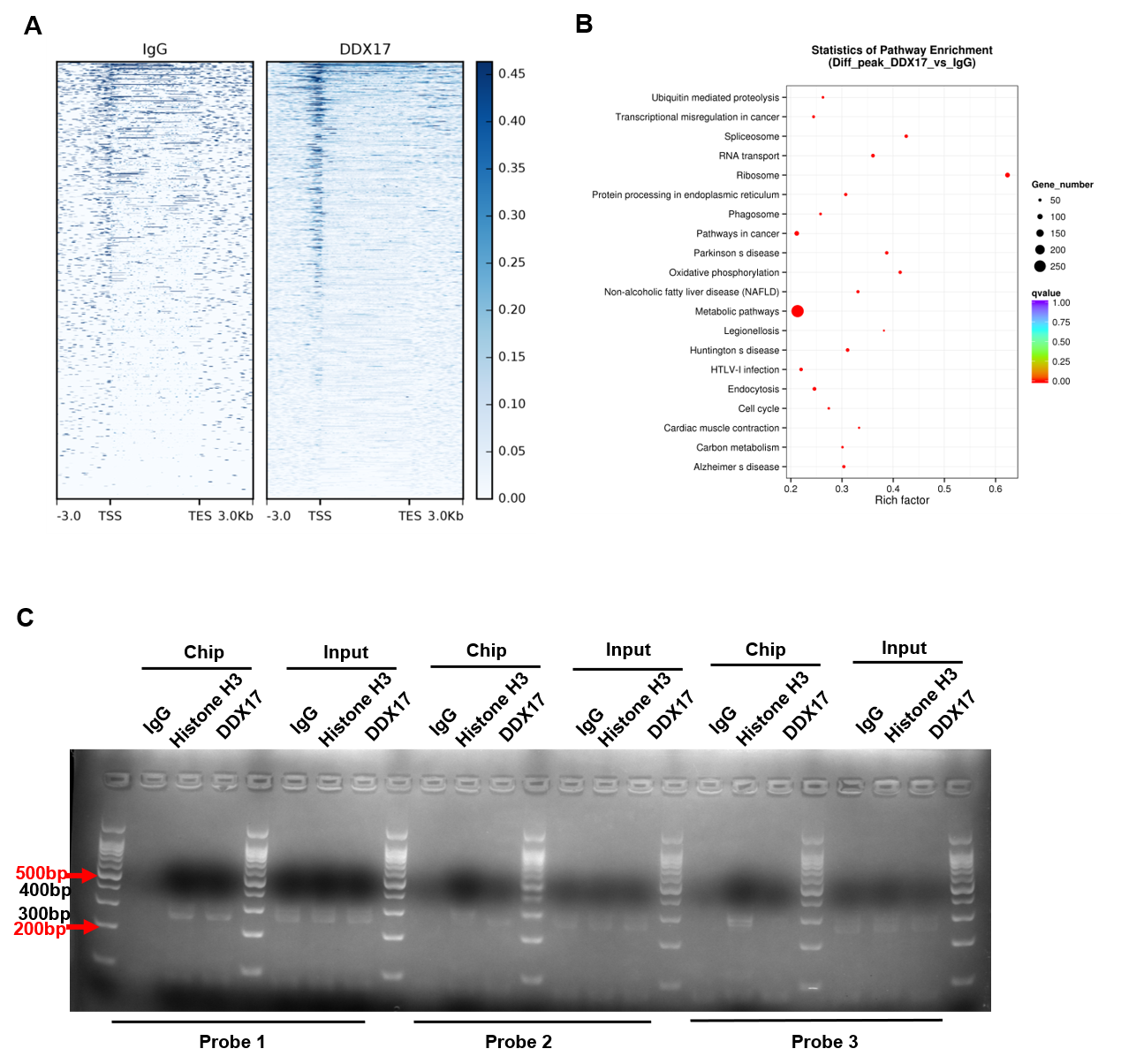

Supplement: Supplementary file 1 — Supplementary Figures [file CTM2-14-e1529-s003.zip › ctm/ctm21529-sup-0001-figureS7.png]

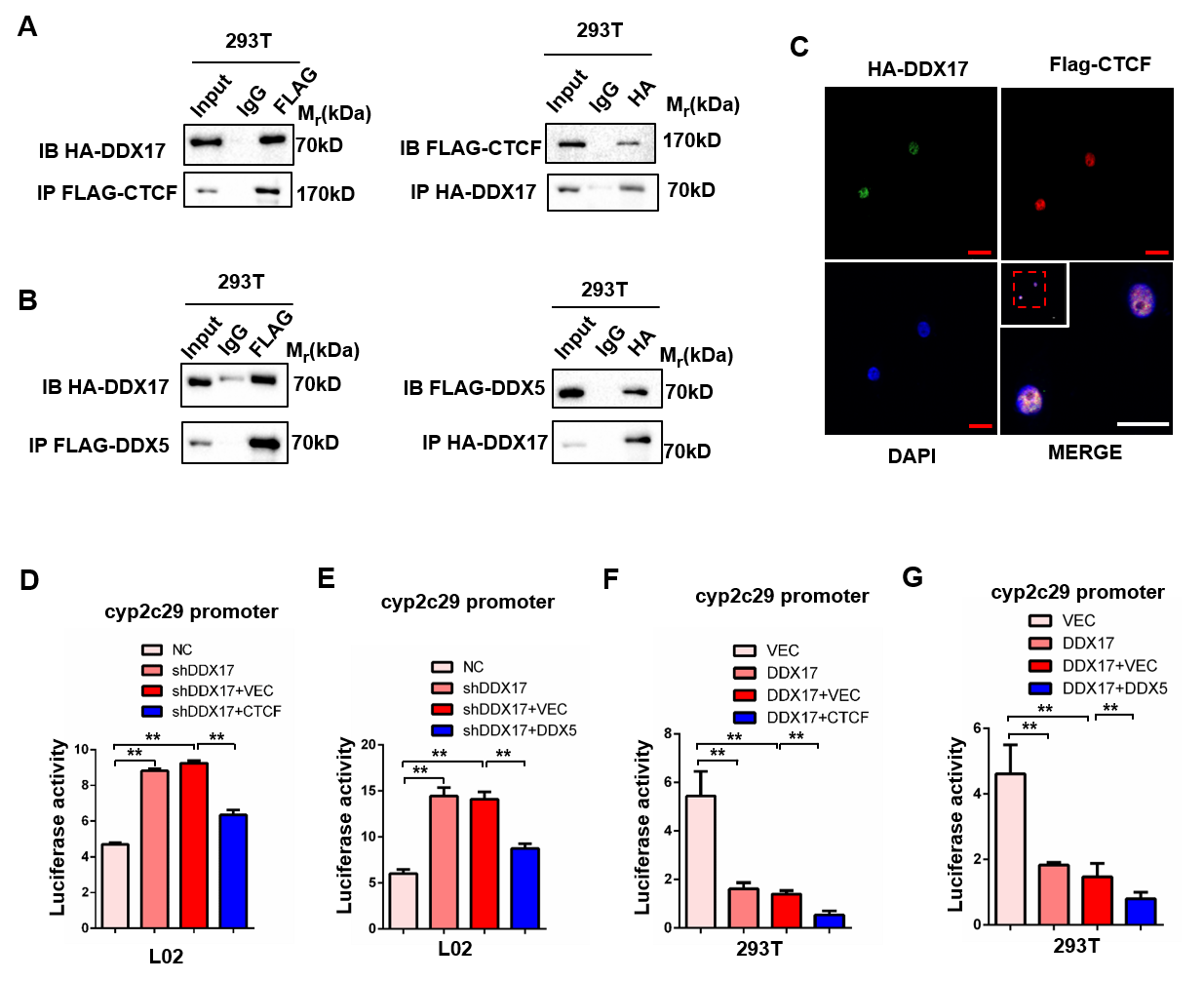

Supplement: Supplementary file 1 — Supplementary Figures [file CTM2-14-e1529-s003.zip › ctm/ctm21529-sup-0001-figureS8.png]

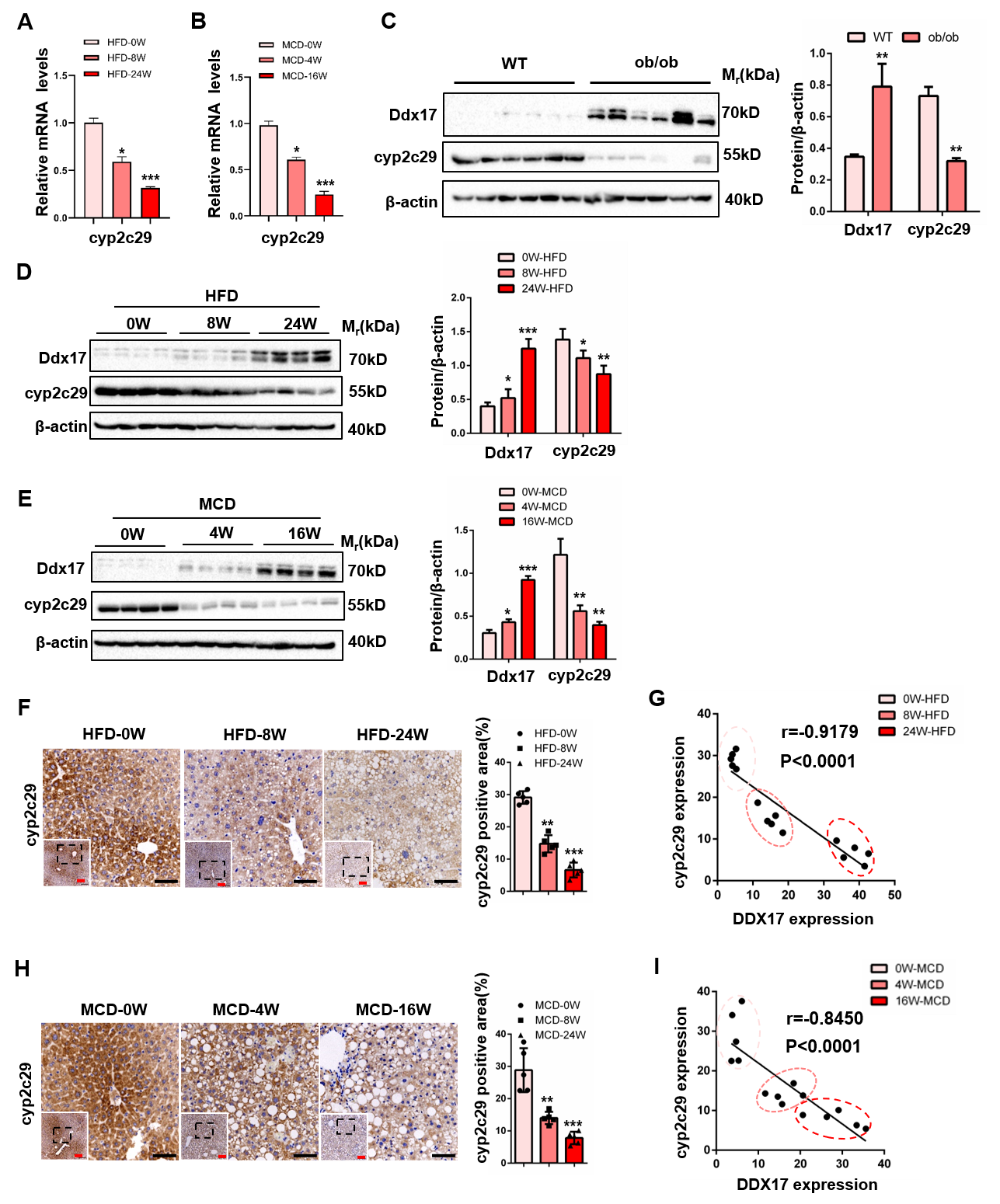

Supplement: Supplementary file 1 — Supplementary Figures [file CTM2-14-e1529-s003.zip › ctm/ctm21529-sup-0001-figureS9.png]
