# Supplementary material for: DEAD‐Box Helicase 17 exacerbates non‐alcoholic steatohepatitis via transcriptional repression of cyp2c29, inducing hepatic lipid metabolism disorder and eliciting the activation of M1 macrophages
Source: Clin Transl Med. 2024 Feb 1;14(2):e1529. doi: 10.1002/ctm2.1529 (PMC10835191; doi:10.1002/ctm2.1529)
Supplement: Supplementary file 2 — Supplementary Materials [file CTM2-14-e1529-s001.docx]

**DEAD-Box Helicase 17 exacerbates nonalcoholic steatohepatitis via transcriptional repression of cyp2c29, inducing hepatic lipid metabolism disorder and eliciting the activation of M1 macrophages**

**Table of Contents**

**Supplemental figures and legends**

Fig. S1. DDX17 expression is upregulated in fatty liver and correlates with NASH progression. (Page 3)

Fig. S2. DDX17 plays a role in lipid accumulation in hepatocytes. (Page 4)
Fig S3. The construction of hepatocyte-specific DDX17 knockout mice. (Page 6)

Fig. S4. **Representative morphology of livers and NAS scores from DDX17-Flox and DDX17-CKO mice** **fed with ND or HFD.** (Page 7)

Fig. S5. **Metabolism detection and food intake of DDX17-Flox and DDX17-CKO mice that were fed MCD.** (Page 8)

Fig. S6. **DDX17 alters the landscape of lipid metabolism, inflammation and fibrosis in murine NASH.**(Page 9)

Fig. S7. **DDX17 binds the promoter of cyp2c29.** (Page 10)

Fig. S8. **DDX17 cooperates with CTCF and DDX5 in repressing the cyp2c29 gene expression**  (Page 11)

Fig. S9. **The negative correlation between DDX17 and cyp2c29 in NASH mice** (Page 12)

Fig. S10. **The negative correlation between DDX17 and cyp2c29 in NASH mice** (Page 14)

Fig. S11. **Hepatocyte-specific DDX17 deficiency alters lipid composition in murine NASH.** (Page 15)

Fig. S12. **DDX17 promotes the progression of liver inflammation and fibrosis in murine NASH.** (Page 16)

Fig. S13. **DDX17 promotes the progression of liver inflammation and fibrosis in murine NASH.** (Page 17)

**Supplemental Tables**

Supplementary Table 1 The clinical information and histologic features of subjects indicated in this study. (Page 18)

Supplementary Table 2 Information about expression vectors used in this study. (Page 19)

Supplementary Table 3 Information about reagents utilized in this study. (Page 20)

Supplementary Table 4. Information about antibodies utilized in this study (Page 21)

Supplementary Table 5. Sequence information of shRNAs, siRNAs, and PCR primers. (Page 22 -27)

Supplementary Table 6. Lipid metabolism related genes of DDX17 between Flox-MCD and CKO-MCD group. (Page 28-30)

Supplementary Table 7. Inflammation related genes of DDX17 between Flox-MCD and CKO-MCD group. (Page 30-32)

Supplementary Table 8. Liver fibrosis related genes of DDX17 between Flox-MCD and CKO-MCD group. (Page 32-34)

**Additional files**

Additional files 1. Flow diagram for participants included in the study. (Page 33)

Additional files 2. CUT&Tag analysis: (X101SC21061883-Z01-J001-B1-36). (This file is uploaded separately as an attachment)

Additional files 3. Additional files of lipidomic analysis: Additional files: (01-06). (This file is uploaded separately as an attachment)

**Supplementary figure legends**


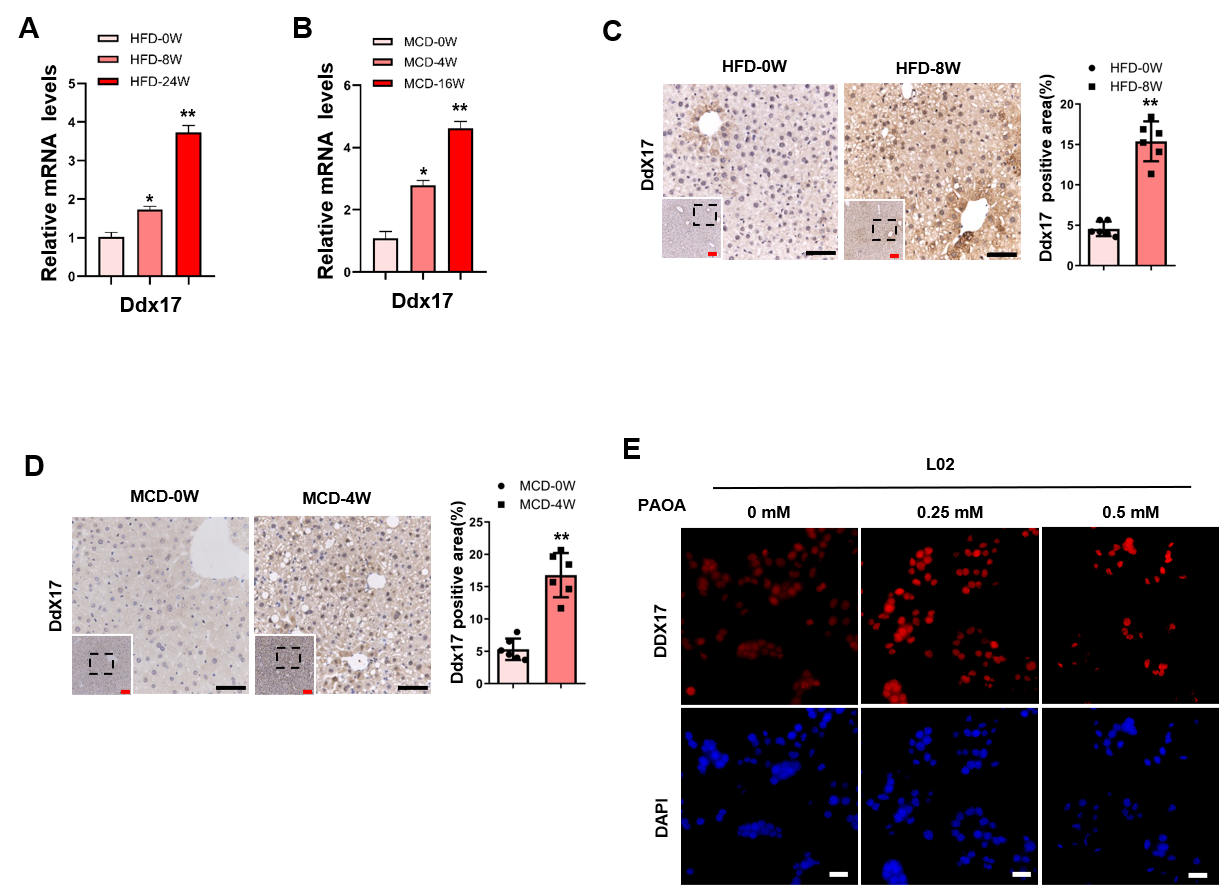


**Fig. S1. DDX17 expression is upregulated in fatty liver and correlates with NASH progression.**

(A) mRNA levels of Ddx17 in the livers of C57BL/6J mice that were on a HFD over time (*p<0.05, **p<0.01). (B) mRNA levels of Ddx17 in the livers of C57BL/6J mice that were on a MCD over time (*p<0.05, **p<0.01). (C) Representative immunohistochemistry images (left) and quantification (right) of Ddx17 expression in the livers of C57BL/6J mice treated with ND or a HFD for 8 weeks (**p<0.01; Red bar, 100um; Black bar, 100um; n=6 per group). (D) Representative immunohistochemistry images (left) and quantification (right) of Ddx17 expression in the livers of C57BL/6J mice treated with ND or a MCD for 4 weeks (**p<0.01; Red bar, 100um; Black bar, 100um; n=6 per group). (E) Immunofluorescence of DDX17 expression in L02 hepatocytes that was treated with PAOA for 24h (PA;0.25mM, OA; 0.5mM; White bar, 50um). For (A) and (B), statistical analysis was carried out by one-way ANOVA. For (C-D), statistical analysis was carried out by two-tailed Student’s t test. All data are shown as the mean ± SD.


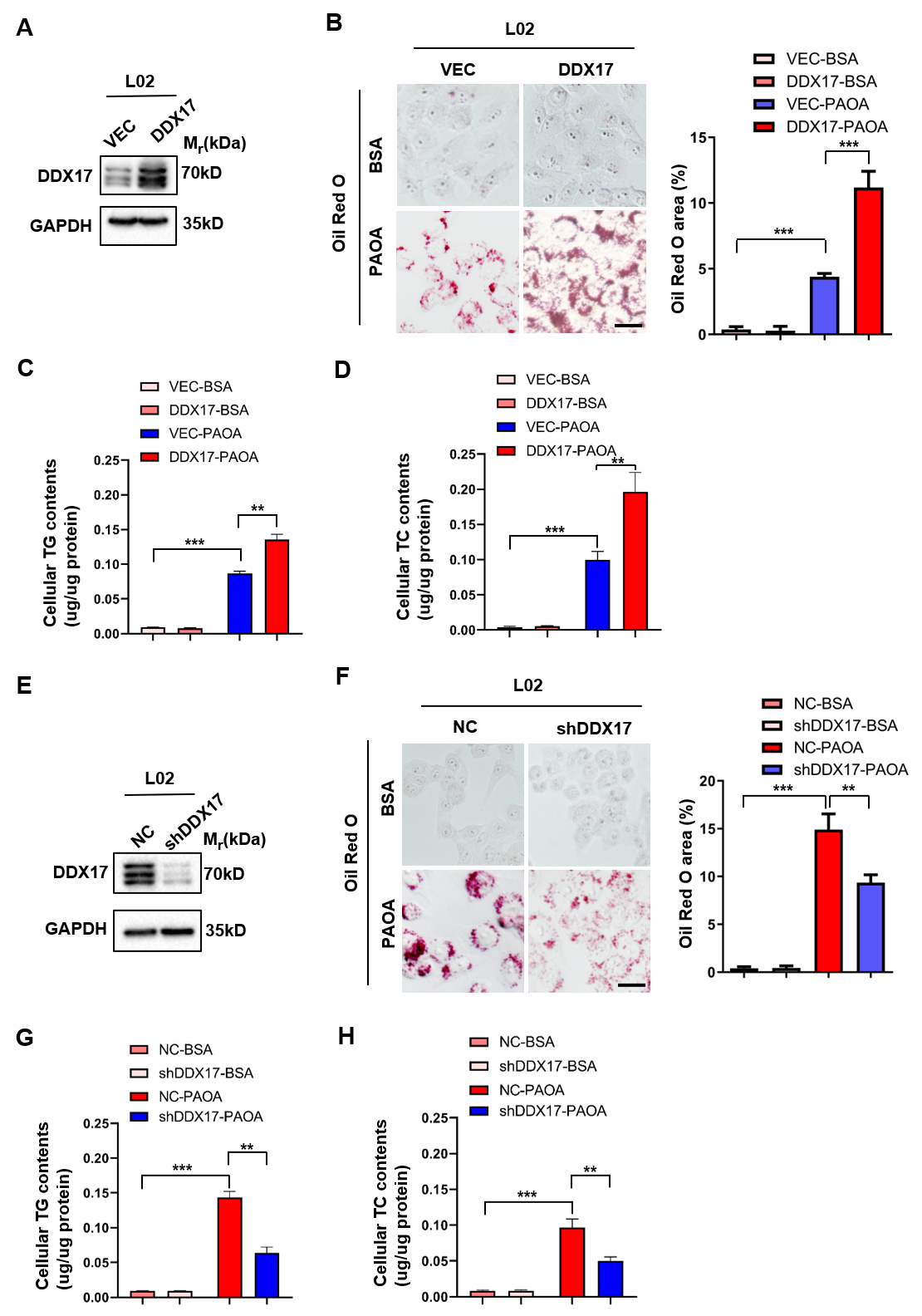


**Fig. S2. DDX17 plays a role in lipid accumulation in hepatocytes**

(A) Western blot analysis of DDX17 protein expression in L02 hepatocytes with DDX17 gene overexpression by lentiviral vector or vector control; n=3 independent experiments.

(B) Representative images (left) and quantitative results (right) of Oil Red O-stained L02 hepatocytes subjected to control or DDX17 lentiviral infection followed by BSA or PAOA (PA;0.25mM, OA; 0.5mM) treatment for 12 hours (***p<0.01; n=3 independent experiments; Scale bar, 25um).

(C-D) Cellular TG (C) and Cellular TC (D) levels from L02 hepatocytes subjected to control or DDX17 lentiviral infection followed by BSA or PAOA (PA;0.25mM, OA; 0.5mM) treatment for 12 hours (**p<0.01, ***p<0.001; n=3 independent experiments).

(E) Western blot analysis of DDX17 protein expression in L02 hepatocytes infected with pLKO.1 control vector or shDDX17; n=3 independent experiments. (F) Representative images (left) and quantitative results (right) of Oil Red O-stained L02 hepatocytes subjected to pLKO.1 control vector or shDDX17 lentiviral infection followed by BSA or PAOA (PA;0.25Mm, OA; 0.5mM) treatment for 12 hours (**p<0.05, ***p<0.001; n=3 independent experiments; Scale bar, 25um).

(G-H) Cellular TG (G) and Cellular TC (H) levels from L02 hepatocytes infected with pLKO.1 control vector or shDDX17 followed by BSA or PAOA (PA;0.25mM, OA; 0.5mM) treatment for 12 hours (**p<0.01, ***p<0.001; n=3 independent experiments). For (B-D) and (F-H), statistical analysis was carried out by one-way ANOVA. All data are shown as the mean ± SD.


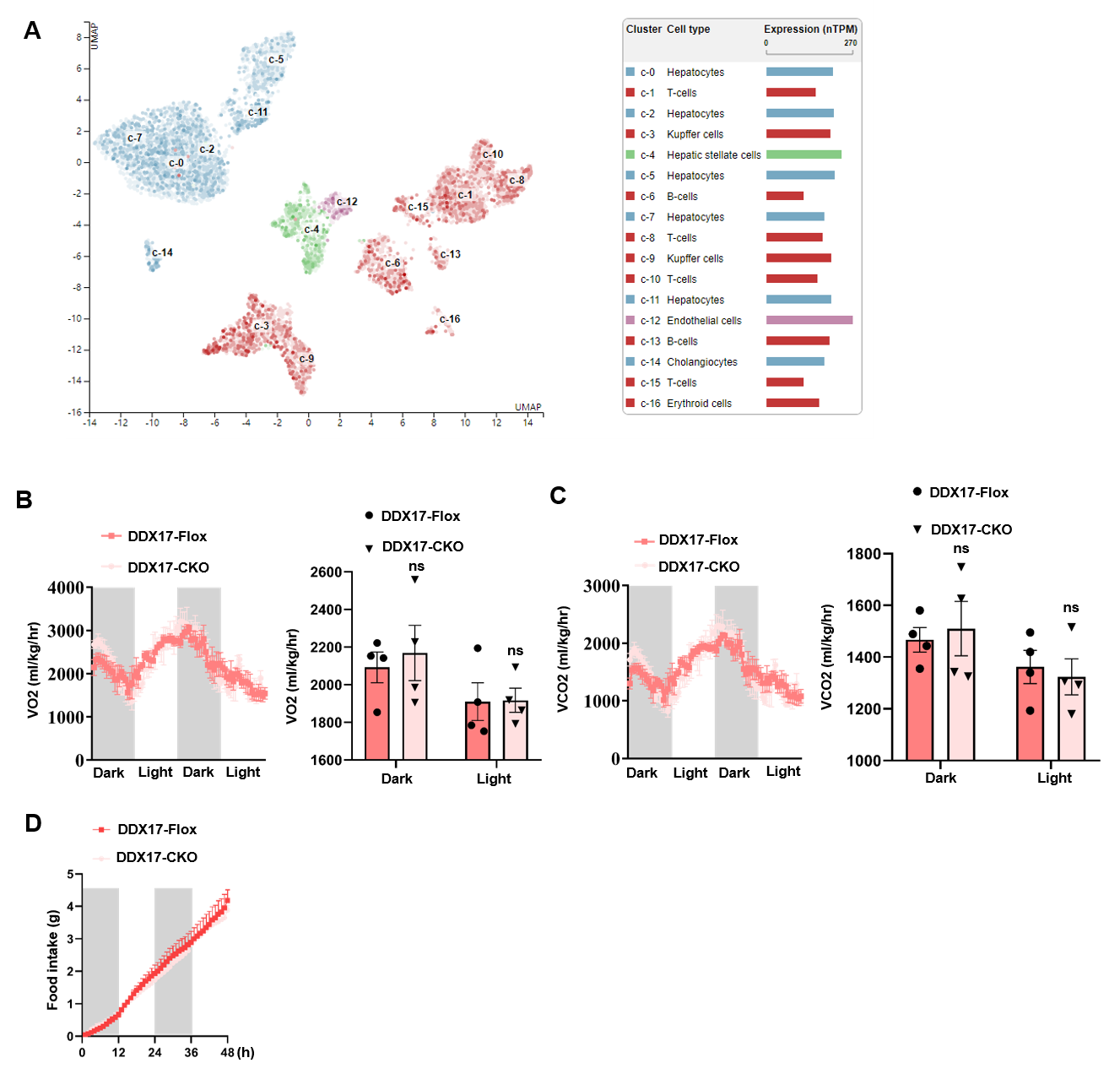


**Fig S3. The construction of hepatocyte-specific DDX17 knockout mice.**

(A) Uniform manifold approximation and projection (UMAP) plot and histogram showing the expression of DDX17 in different liver cells, including hepatocytes, Kupffer cells, Hepatic stellate cells, Endothelial cells, Cholangiocytes, Erythroid cells, T-cells, and B-cells.

(B and D) Metabolism detection of the two groups. Oxygen consumption (B) and quantification in right panel. Carbon dioxide emission (C) and quantification in right panel (n = 4). (D) Food intake (g) in DDX17-Flox and DDX17-CKO mice that were fed chow diet measured in metabolic cages (n = 4 per group).


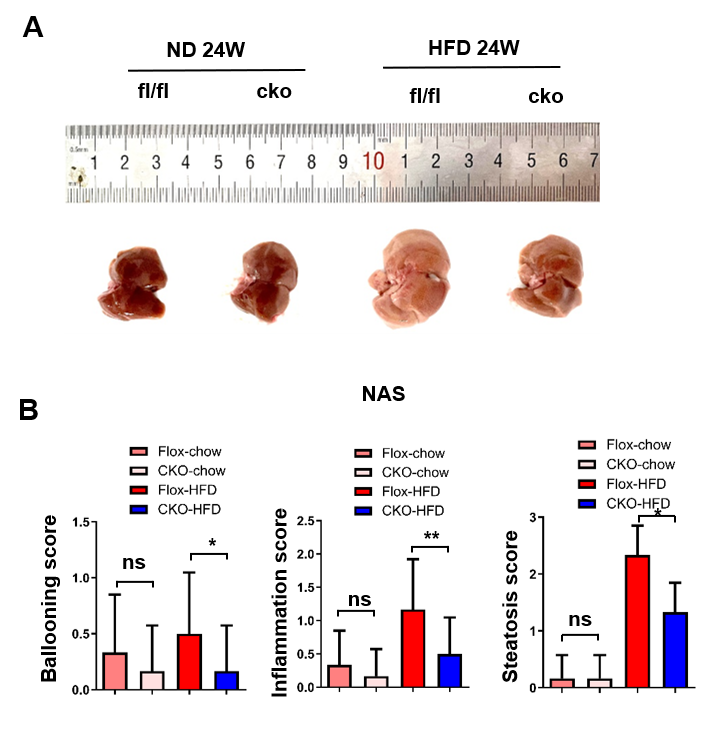


**Fig S4. Representative morphology of livers and NAS scores from DDX17-Flox and DDX17-CKO mice** **fed with ND or HFD.**

(A) Representative morphology of livers from DDX17-Flox and DDX17-CKO mice were fed with ND (n=6 mice) or HFD (n=6 mice) for 24 weeks.

(B) The specific NAS scores including ballooning scores, inflammation scores and steatosis scores of DDX17-Flox and DDX17-CKO mice that were fed chow or HFD for 24 weeks (*p<0.05; **p<0.01, n.s., not significant; n=6 mice per group).


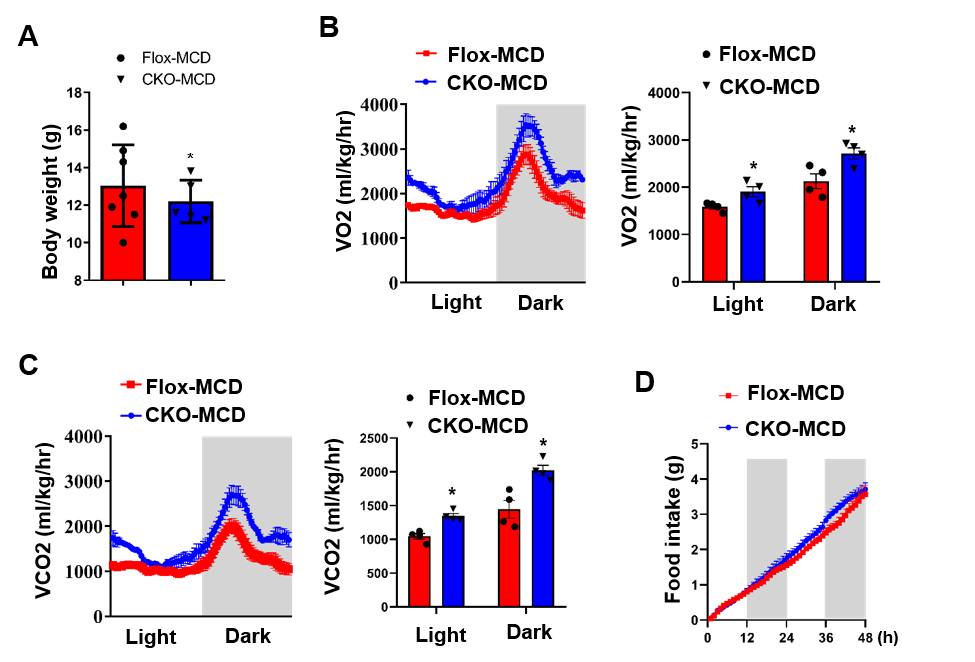


**Fig S5. Metabolism detection and food intake of DDX17-Flox and DDX17-CKO mice that were fed MCD.**

(A and B) Metabolism detection of DDX17-Flox and DDX17-CKO mice that were fed MCD measured in metabolic cages (n = 4 per group). Oxygen consumption (A) and quantification in right panel. Carbon dioxide emission (B) and quantification in right panel (n = 4). (C) Food intake (g) in DDX17-Flox and DDX17-CKO mice that were fed MCD measured in metabolic cages (n = 4 per group).


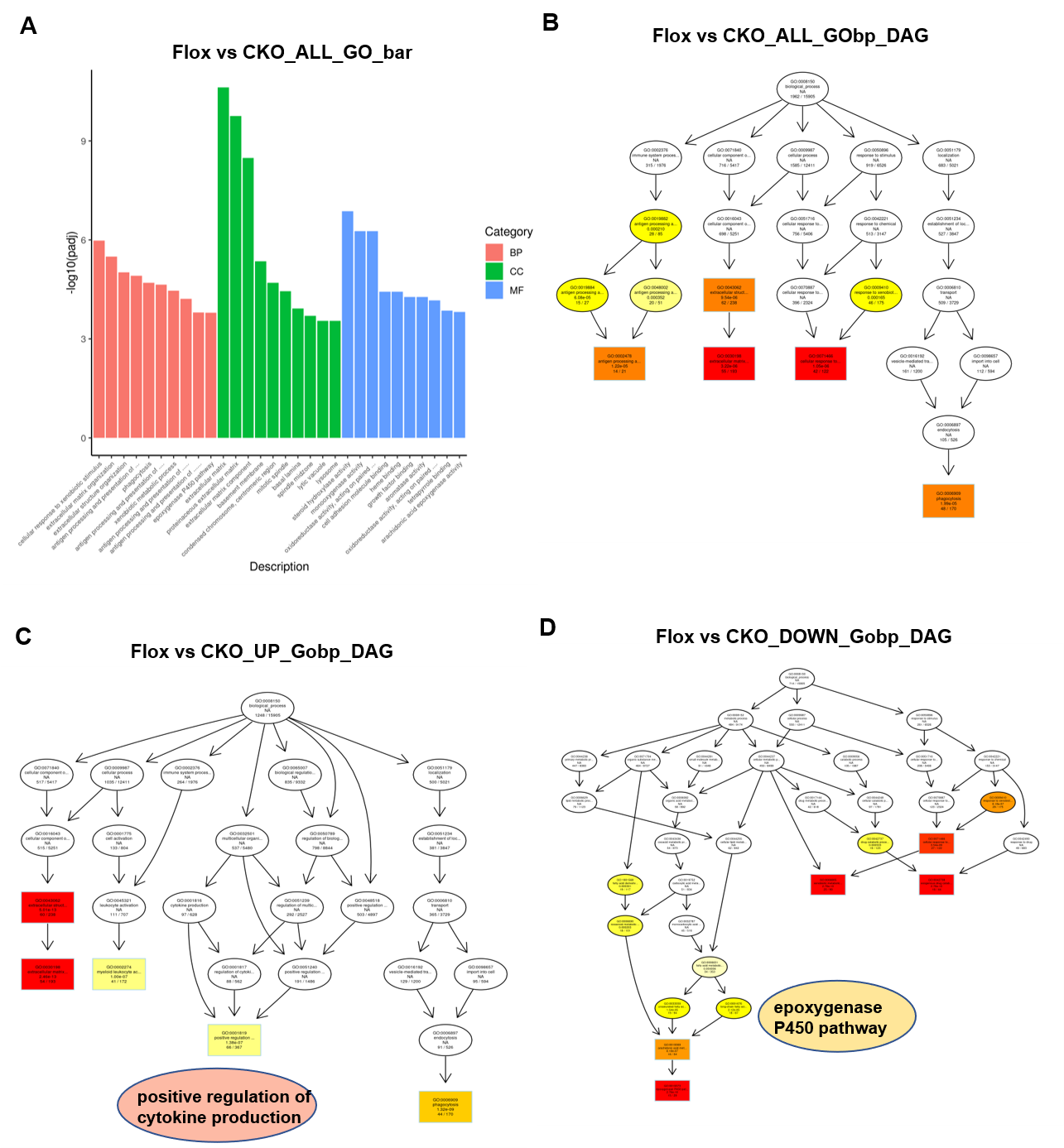


**Fig. S6. DDX17 alters the landscape of lipid metabolism, inflammation and fibrosis in murine NASH.**

(A-D) GO function enrichment analysis of differential genes between DDX17-CKO and DDX17-Flox mice were shown in bar(A) and DAG (GO biological process, GO cellular component and molecular function) (B-D).


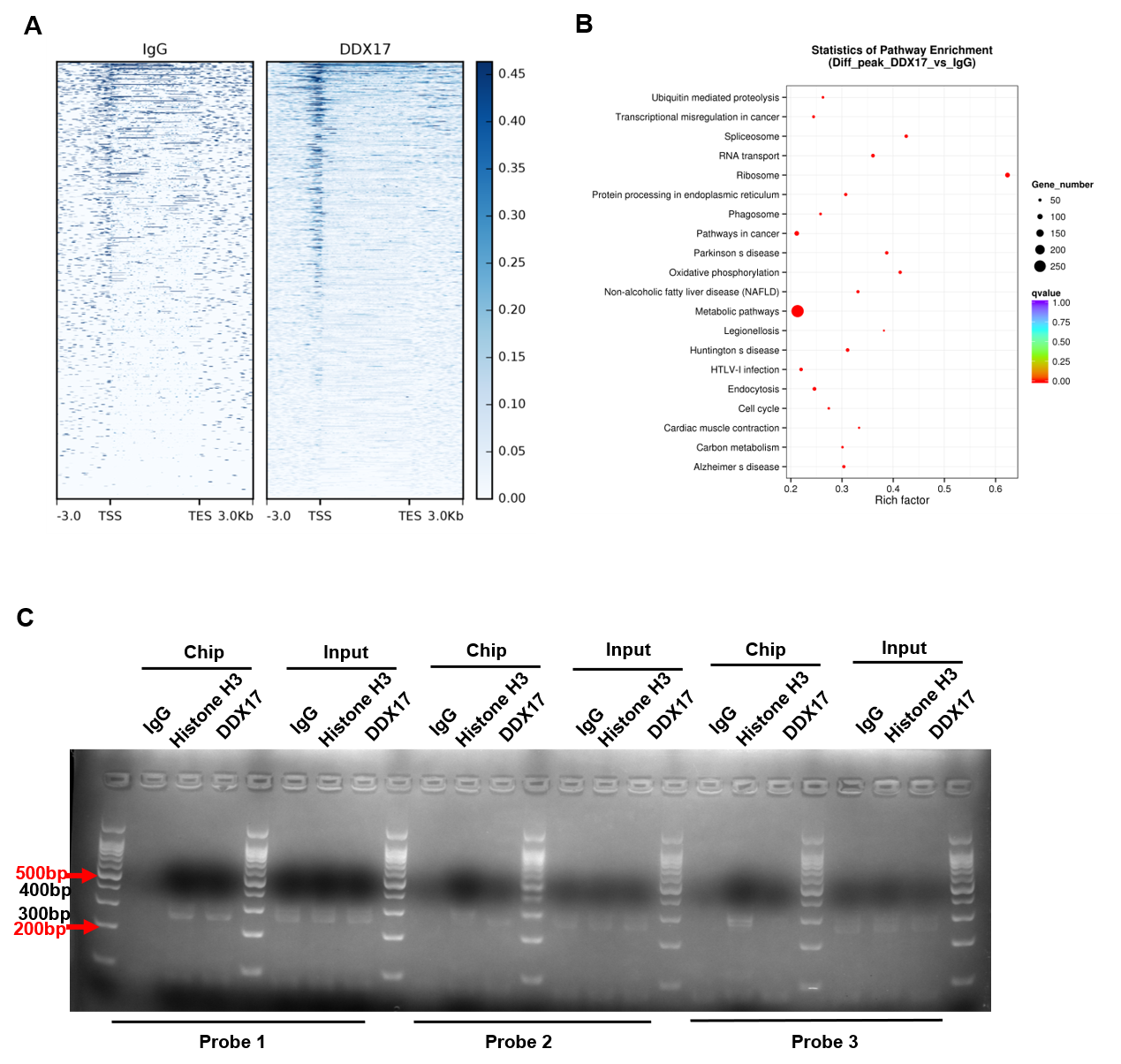


**Fig. S7. DDX17 binds the promoter of cyp2c29.**

(A) Heatmap of the binding sites of DDX17 and IgG at the positions -3.0 kb upstream to +3.0 kb downstream relative to transcription start site (TSS) and transcription end sites (TES). (B) KEGG enrichment analysis based on differential peaks between DDX17 and IgG.

(C) The PCR of Chip immunoprecipitation of L02 cells showing the binding sites.


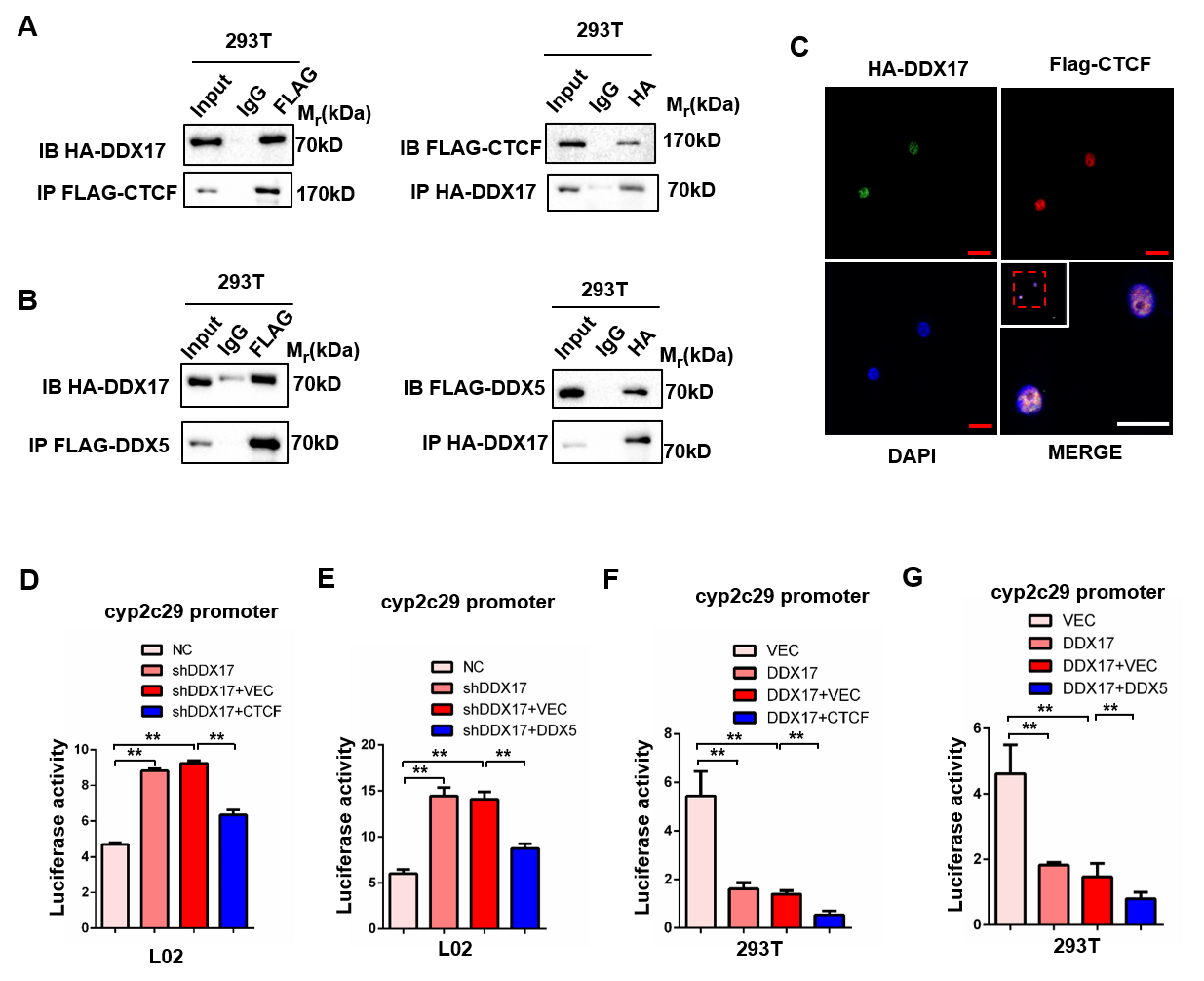


**Fig. S8. DDX17 cooperates with CTCF and DDX5 in repressing the cyp2c29 gene expression**

(A) CO-IP analysis of 293T transfected with HA-DDX17 and Flag-CTCF. (B) CO-IP analysis of 293T transfected with HA-DDX17 and Flag-DDX5. (C) Co-localization of DDX17 and CTCF was analyzed by immunofluorescence assays in 293T cell transfected with HA-DDX17 and Flag-CTCF (red bar, 20um, white bar, 20um). (D-G) The relative luciferase activity of cyp2c29 promoter were respectively detected in indicted cell groups in L02(D, E), and 293T (F, G) cells (**p<0.01; n=3 independent experiments). Statistical analysis was carried out by one-way ANOVA. All data are shown as the mean ± SD.


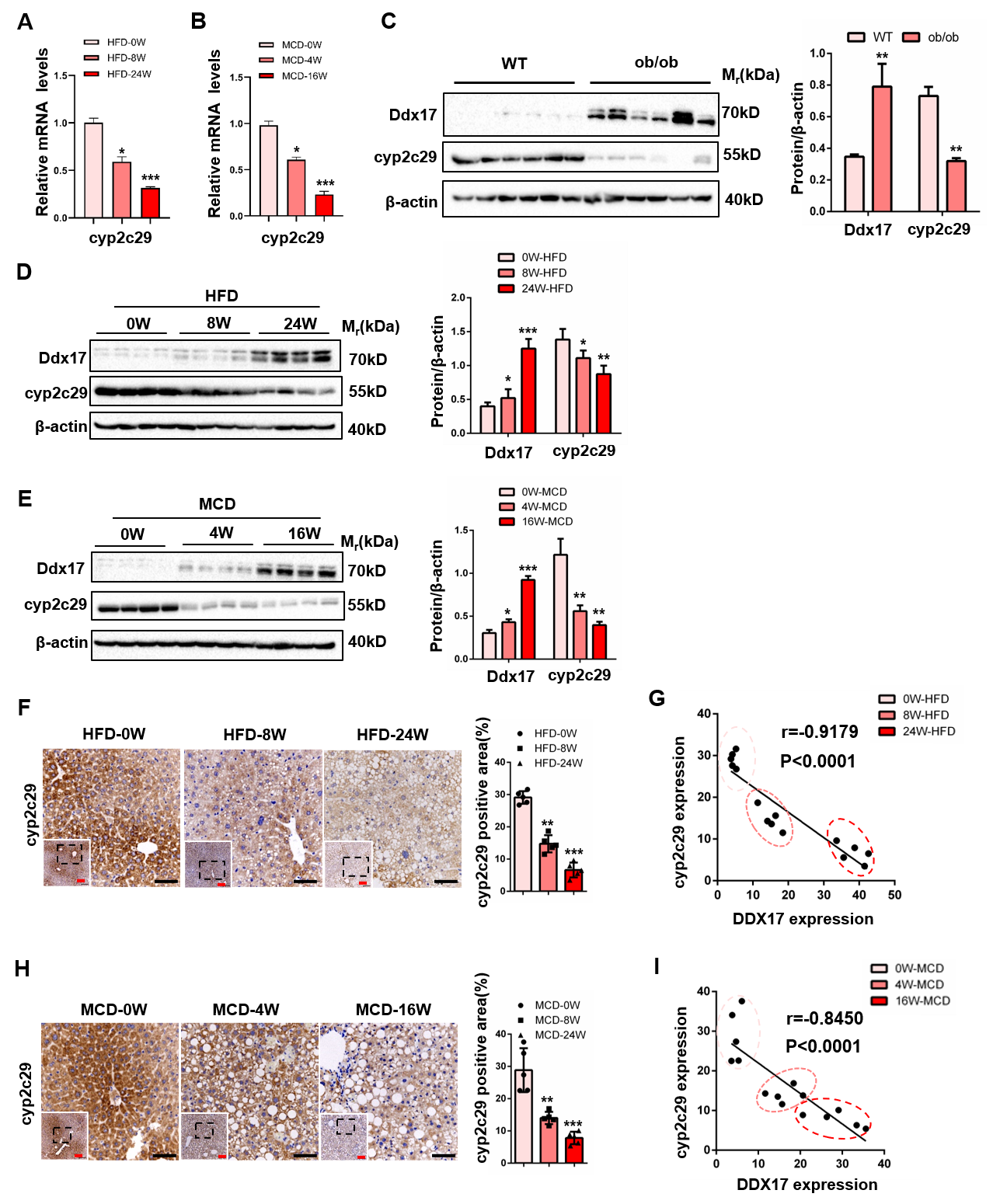


**Fig. S9. The negative correlation between DDX17 and cyp2c29 in NASH mice**

(A) mRNA levels of cyp2c29 in the livers of C57BL/6J mice that were on a HFD over time (*p<0.05; ***p<0.001; n = 4 mice per group). (B) mRNA levels of cyp2c29 in the livers of C57BL/6J mice that were on a MCD over time (*p<0.05; ***p<0.001; n = 4 mice per group).

(C) Representative western blots (left) and quantification (right) of cyp2c29 expression in the livers of C57BL/6J wild-type mice and ob/ob mice fed for 8 weeks (**p<0.01; n=6 per group). (D) Representative western blots (left) and quantification (right) of Ddx17 and cyp2c2c9 expression in the livers of C57BL/6J mice that were on a HFD over time (*p<0.05; **p<0.01; ***p<0.001; n = 4 western blots). (E) Representative western blots (left) and quantification (right) of Ddx17 and cyp2c2c9 expression in the livers of C57BL/6J mice that were on a MCD over time (*p<0.05; **p<0.01; ***p<0.001; n = 4 western blots). (F) Representative immunohistochemistry images (left) and quantification (right) of Ddx17 and cyp2c2c9 expression in the livers of C57BL/6J mice treated with normal diet (ND) or a HFD over time (**p<0.01, ***p<0.001; Bar, 100um; n = 6 per group). (G) The scatter plot showed significantly negative correlation between Ddx17 and Cyp2c29 in the livers of C57BL/6J mice treated with ND or a HFD over time. The correlation coefficient and p value plotted on the chart. (H) Representative immunohistochemistry images (left) and quantification (right) of Ddx17 and cyp2c2c9 expression in the livers of C57BL/6J mice treated with ND or a MCD over time (**p<0.01, ***p<0.001; Bar, 100um; n = 6 per group). (I) The scatter plot showed significantly negative correlation between Ddx17 and Cyp2c29 in the livers of C57BL/6J mice treated with ND or a MCD over time. The correlation coefficient and p value plotted on the chart. For (A-B), (D-E), (F) and (H) statistical analysis was carried out by one-way ANOVA.


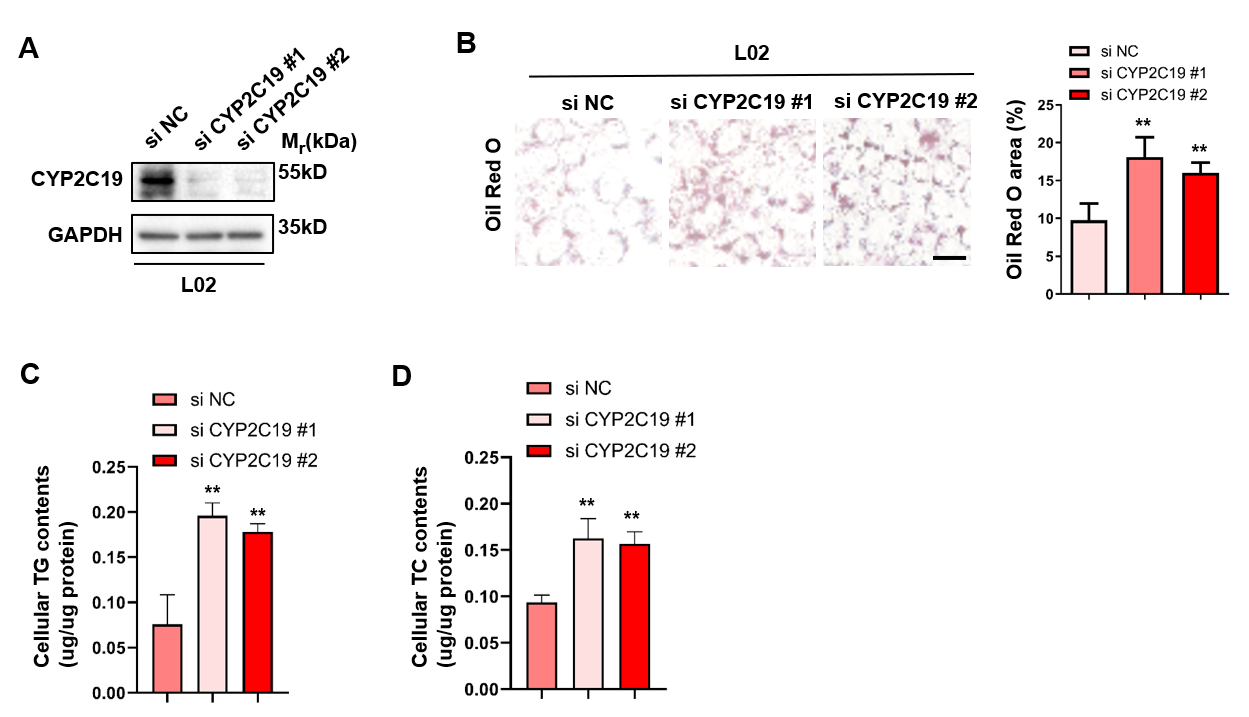


**Fig. S10. The negative correlation between DDX17 and cyp2c29 in NASH mice**

1. Western blot analysis of DDX17 protein expression in L02 hepatocytes infected with si NC or si CYP2C19; n=3 independent experiments. (B) Representative images (left) and quantitative results (right) of Oil Red O-stained L02 hepatocytes nfected with si NC or si CYP2C19 followed by PAOA (PA;0.25Mm, OA; 0.5mM) treatment for 12 hours (**p<0.05; n=3 independent experiments; Scale bar, 25um). (C) Cellular TG (C) and Cellular TC (D) levels from L02 hepatocytes infected with si NC or si CYP2C19 followed by PAOA (PA;0.25mM, OA; 0.5mM) treatment for 12 hours (**p<0.01; n=3 independent experiments).


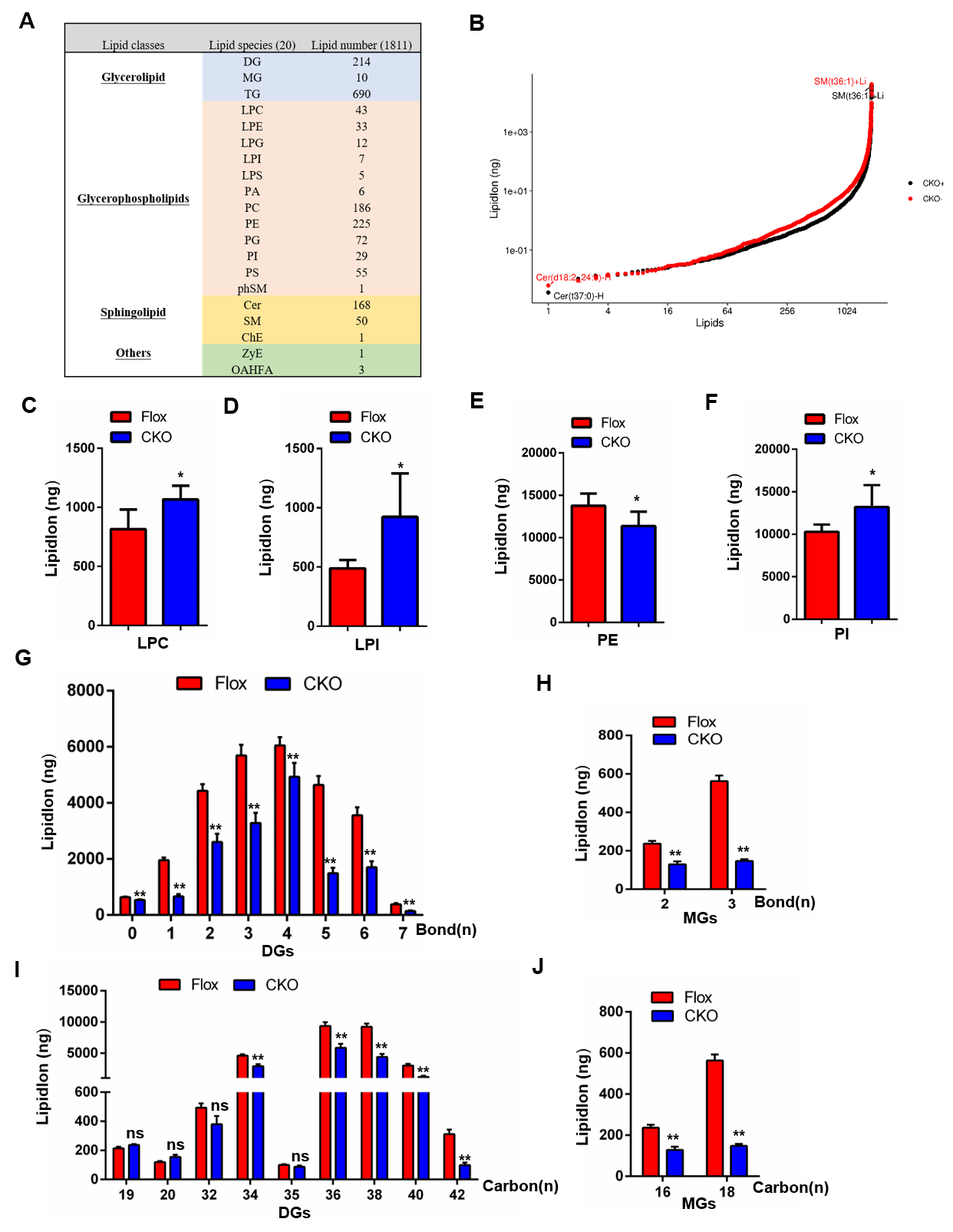


**Fig. S11. Hepatocyte-specific DDX17 deficiency alters lipid composition in murine NASH.**

(A) The numbers of total lipids and subclasses in the absolute quantitative lipidomics of DDX17-CKO mice and their corresponding DDX17-Flox controls after MCD consumption.

(B) The dynamic distribution of lipid content in the livers of DDX17-CKO mice and their corresponding DDX17-Flox controls after MCD consumption was shown. (C-F) The lipid content of LPC, LPI, PE and PI between the indicated group. (*p<0.05). (G-J) The lipid chain length and saturation analysis of DAGs and MAGs between the indicated group. The horizontal axis represents lipid molecules of different carbon chain lengths (G-H) and bond numbers (I-J), and the vertical axis represents the amount of lipid molecules (**p<0.01, n.s., not significant).


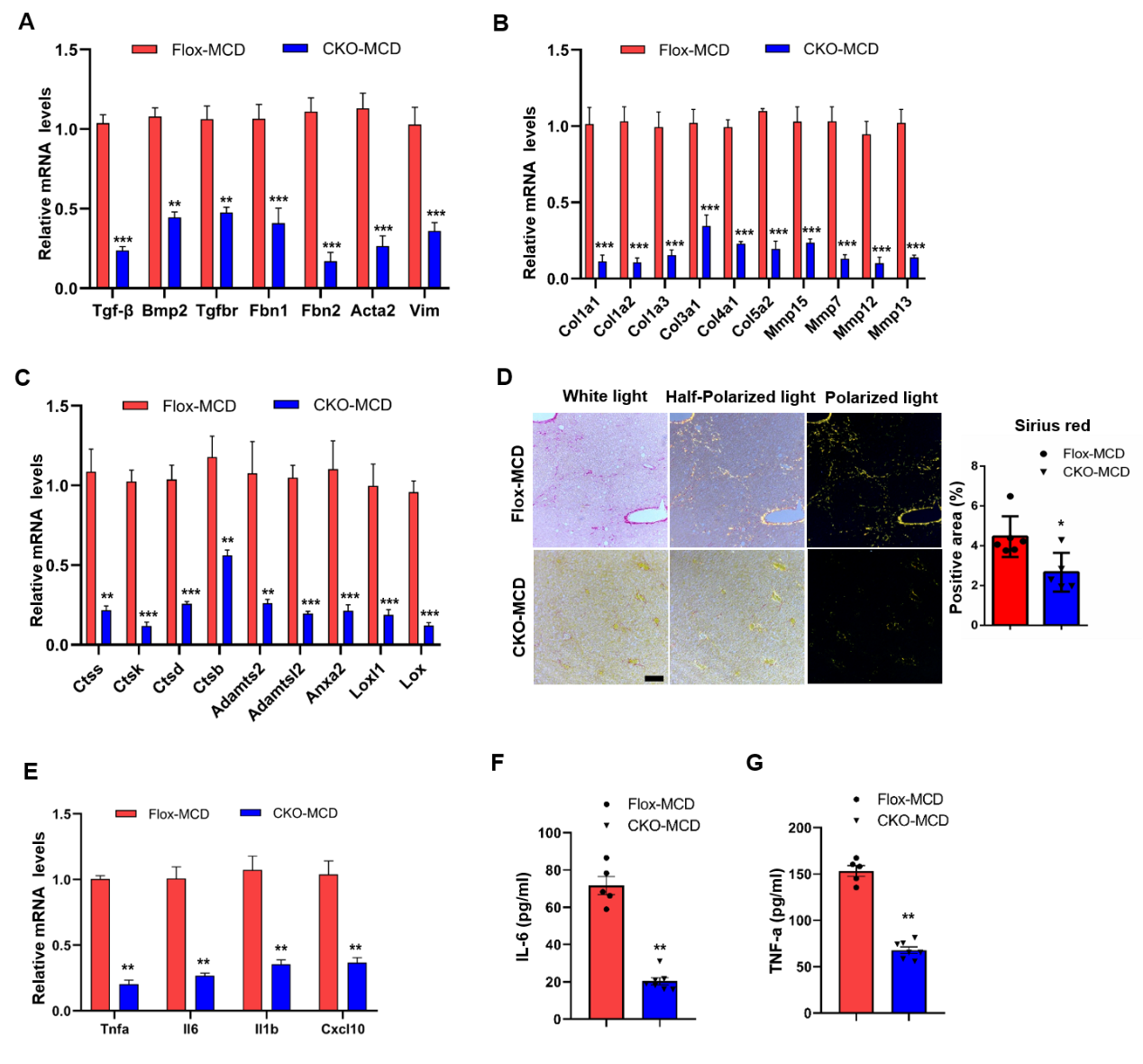


**Fig. S12. DDX17 promotes the progression of liver inflammation and fibrosis in murine NASH.**

(A-C) Relative mRNA levels of fibrosis-related genes in the livers of MCD-fed DDX17-CKO and DDX17-Flox (**p<0.01, ***p<0.01). (D) Sirius red staining of liver sections in DDX17-Flox group (n=7 mice per group) and DDX17-CKO group (n=5 mice per group) after MCD administration (*p<0.05; Scale bar, 100um). The positive areas were analyzed and quantified (right). (E) Relative mRNA levels of Tnfa, Il6, Il1b, and Cxcl10 in the livers of MCD-fed DDX17-CKO and DDX17-Flox (**p<0.01, ***p<0.01). (F and G) The serum ELISA levels of pro-inflammatory cytokines IL-6 and TNF-a in two groups.


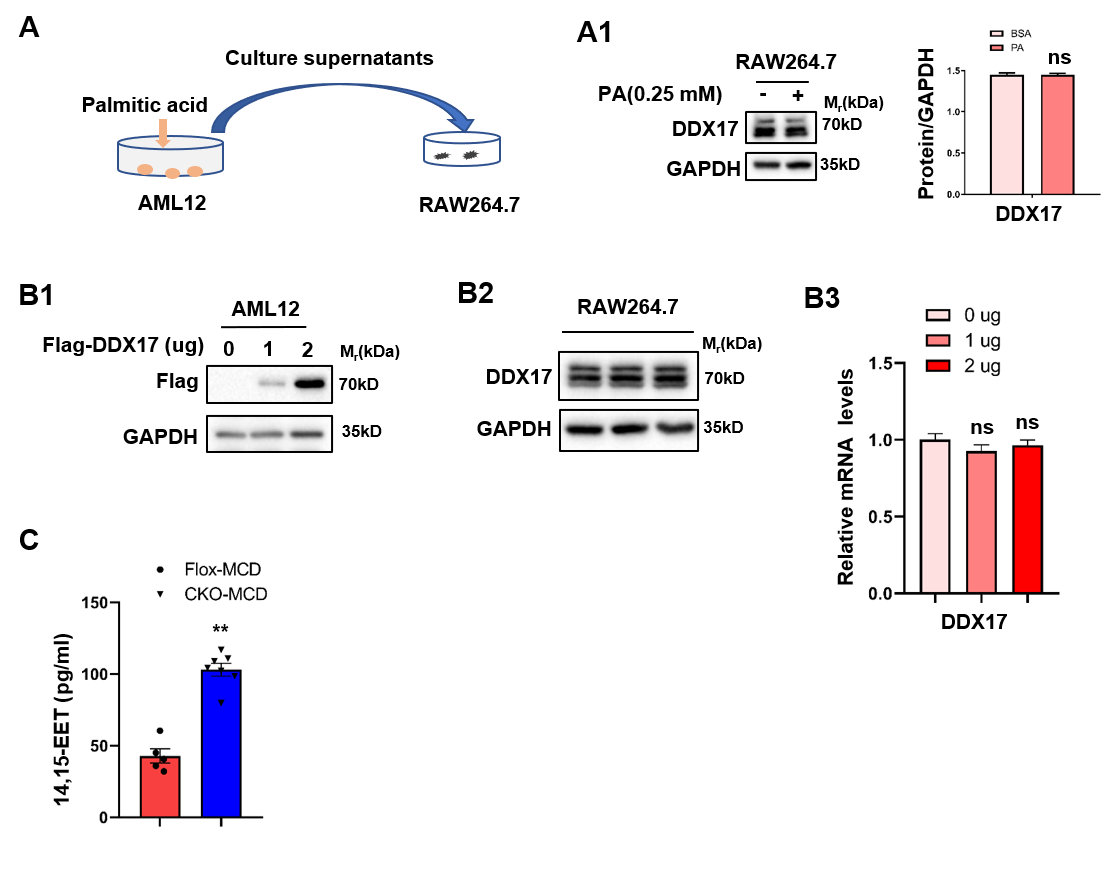


**Fig. S13. DDX17 promotes the progression of liver inflammation and fibrosis in murine NASH.**

(A) Schematic of RAW264.7 cells co-cultured culture supernatants of AML12 cells with or without PA treatment. (A1) Western blot analysis of DDX17 protein expression in RAW264.7 cells treated with culture supernatants of AML12 cells with or without PA treatment.; (B1) Western blot analysis of DDX17 protein expression in AML12 cells transfected with DDX17 plasmid. (B2) Western blot and RT-PCR (B3) analysis of DDX17 expression in RAW264.7 cells transfected with culture supernatants of AML12 cells transfected with DDX17 plasmid. (C) The serum ELISA levels of 14,15-EET in two groups.

**Supplemental Tables**

**Supplementary Table S1.** The clinical information and histologic features of subjects indicated in this study.

|  | **Non-steatosis** | ***NAFLD*** | ***NASH*** |
| --- | --- | --- | --- |
| All | 10 | 17 | 9 |
| Gender (male) | 8(80.0%) | 14(76.4%) | 4(44.4%) |
| Age (years) | 50.4±17.3 | 42.8±12.2 | 42.56±15.7 |
| BMI (kg/m2) | 21.87±2.5 | 26.5±3.1 | 24.9±3.9 |
| ALT (IU/L) | 27.2±24.0 | 76.1±75.5 | 50.7±48.0 |
| AST (IU/L) | 34.2±19.8 | 59.6±39.4 | 55.7±32 |
| Cholesterol (mmol/L) | 3.5±0.9 | 3.9±1.5 | 4.0±2.1 |
| Triglycerides (mmol/L) | 1.25±0.3 | 2.0±1.5 | 2.3±1.2 |
| HDL (mmol/L) | 0.66±0.2 | 0.69±0.3 | 0.6±0.3 |
| LDL (mmol/L) | 1.53±0.5 | 2.2±1.0 | 2.4±1.5 |
| FBG (mmol/L) | 6.9±3.2 | 10.0±8.2 | 12.8±10.2 |
| Steatosis grade (1/2/3) | 0 | 13/4/0 | 1/3/5 |
| Lobular inflammation (0/1/2/3) | 0 | 13/4/0/0 | 1/2/4/2 |
| Ballooning (0/1/2/3) | 0 | 0/17 | 1/4/5 |
| Fibrosis (0/1/2/3) | 0 | 0 | 4/5 |

Data are expressed as mean± SD unless otherwise indicated.

BMI, body mass index; AST, aspartate amino transferase; ALT, alanine amino transferase; HDL, high-density lipoprotein; LDL, low-density lipoprotein; FBG, fasting blood glucose.

**Supplementary Table 2** Information about expression vectors used in this study

| Plasmids | sourses | vectors |
| --- | --- | --- |
| shNC (Human) | Self-cloned | plko.1 |
| shDDX17 (Human) | Self-cloned | plko.1 |
| DDX17 (Lentivirus) (Human) | Genechem | GV350 |
| pCMV-MCS-3*flag vector | ViGene Biosciences | pCMV-MCS-3*flag |
| pCMV-MCS-3*flag-hCTCF | ViGene Biosciences | pCMV-MCS-3*flag |
| pcDNA3.1-3*Flag vector | ViGene Biosciences | pcDNA3.1-3*Flag |
| pcDNA3.1-3*Flag-mCyp2c29 | ViGene Biosciences | pcDNA3.1-3*Flag |
| cyp2c29 promoter | Tsingke | pGL4.17 |
| Flag-DDX17 | Self-cloned | pCDNA3.1- |
| HA-DDX17 | Self-cloned | pCDNA3.1- |
| Flag-DDX5 | Self-cloned | pCDNA3.1- |
| AAV8-TBG-DDX17 | Biodesigngene | AAV8-TBG |

**Supplementary Table 3** Information about reagents utilized in this study

| Reagents | Company | Cat. No. |
| --- | --- | --- |
| OA | Kunchuang Biotechnology | SYSJ-KJ005 |
| PA | Kunchuang Biotechnology | SYSJ-KJ006 |
| (±)14(15)-EET | Cayman Chemical | 197508-62-6 |
| 14,15-EE-5(Z)-E | Cayman Chemical | 519038-92-7 |
| MG-132 | MedChemExpress | HY-13259 |
| Cycloheximide | MedChemExpress | HY-12320 |
| Dexamethasone | Sigma-Aldrich | D4902 |
| ITS (100X) | Sigma-Aldrich | 13146 |
| DMEM/F12 | Gibco | C11330500BT |
| Hyperactive Universal CUT&Tag Assay Kit | Vazyme | / |
| TD903-01/02 | Vazyme | #7E570A1 |
| TD202-01 | Vazyme | #7E411J0 |
| N411-01 | Vazyme | #7E442H0 |
| EQ121-01 | Vazyme | #7E481I0 |
| SimpleChIP® Plus Sonication Chromatin IP Kit | CST | #56383 |
| Goat anti Rabbit | Abcam | #Ab206-01 |
| Cell lysis buffer for Western and IP | Beyotime Biotechnology | P0013 |
| DAPI dihydrochloride | MedChemExpress | HY-D0814 |
| TRIzol™ Reagent | Thermo Fisher Scientific | 15596018 |
| BD Matrigel™ Matrix | BD Biosciences | 356235 |
| Pierce™ ECL Western Blotting Substrate | Thermo Fisher Scientific | 32106 |
| Mouse TNF-alpha ELISA Kit | ABclonal | RK00027 |
| Mouse IL-6 ELISA Kit | ABclonal | RK00008 |
| BIOTARGET 14,15-EET/DHET HYPERTENSION ELISA KIT CAT | R&D Detroit | # DH2 |

**Supplementary Table 4** Information about antibodies utilized in this study

| Antibodies | Company | Cat# | Species |
| --- | --- | --- | --- |
| DDX17 | Proteintech | 19910-1-AP | Rabbit polyclonal |
| DDX17 | Abcam | ab70184 | Rabbit polyclonal |
| DDX17 | Bethyl Laboratories | A300-509A | Rabbit polyclonal |
| Cyp2c29 | Proteintech | 16546-1-AP | Rabbit polyclonal |
| Collagen I | CST | #72026 | Rabbit mAb |
| TGF-beta | CST | #3709 | Rabbit polyclonal |
| a-SMA | CST | #19245 | Rabbit mAb |
| F4/80 | CST | #70076 | Rabbit mAb |
| CD206 | CST | #24595 | Rabbit mAb |
| CD86 | Servicebio | GB13585 | Rabbit mAb |
| P-STAT1 Y701 | CST | # 9167 | Rabbit mAb |
| P-STAT1 S727 | CST | # 8826 | Rabbit mAb |
| STAT1 | CST | # 14994 | Rabbit mAb |
| P-STAT6 | CST | # 56554 | Rabbit mAb |
| STAT6 | CST | # 5397 | Rabbit mAb |
| HA | CST | #3724 | Rabbit mAb |
| FLAG | SIGMA | F1804 | Mouse |
| β-actin | CST | #4970 | Rabbit |
| GAPDH | Proteintech | 60004-1-Ig | Mouse mAb |
| IgG Mouse | Santa Cruz | SC2025 | Mouse |
| IgG Rabbit | CST | #2729P | Rabbit |

**Supplementary Table 5** Sequence information of shRNAs, siRNAs, and PCR primers

| Assay | Genes |  | Sequences |
| --- | --- | --- | --- |
| shRNA | sh-DDX17(Human) | #1 | GACCACAAGTTGATCCAACTA |
|  |  | #2 | GCACAGAAGAAACATGGCAAA |
|  |  | #3 | GCAGAGGATTTCCTTCGTGAT |
| RT-PCR | GAPDH（Mouse） | Forward | AGGTCGGTGTGAACGGATTTG |
|  |  | Reverse | GGGGTCGTTGATGGCAACA |
|  | Ddx17（Mouse） | Forward | TCTTCAGCCAACAATCCCAATC |
|  |  | Reverse | GGCTCTATCGGTTTCACTACG |
|  | cyp2c29 (Mouse) | Forward | ATCTGGTCGTGTTCCTAGCG |
|  |  | Reverse | CAGTAGGCTTTGAGCCCAAATA |
|  | cy3a41b (Mouse) | Forward | AAAGCCGCCTCGATTCTAAGC |
|  |  | Reverse | ACTACATCCCGTGGTACAACC |
|  | cyp2c54 (Mouse) | Forward | AGACAGAGCTATGAAAGAGGGAA |
|  |  | Reverse | GTGAGAAGTGCCTCGTGTTTT |
|  | cyp2c70 (Mouse) | Forward | AGTATGGCCCTGTGTTTACTGT |
|  |  | Reverse | GCCTTGGCTGGTTCTACTGAG |
|  | cyp2c50 (Mouse) | Forward | ACTGTGGTGTTGCATGGATATG |
|  |  | Reverse | GAGAAGCGCCTTGTGTTTTTC |
|  | cyp2j9 (Mouse) | Forward | ATGCGCCTTCCTTTCGTGG |
|  |  | Reverse | CCAGGCTTAGAACATTCCCGTA |
|  | cyp2c40 (Mouse) | Forward | GGCTCACAGCCTATTGTGGTA |
|  |  | Reverse | TCAAAAACCGGAATCCTTCCTC |
|  | cyp2c39 (Mouse) | Forward | GAGGAAGCATTCCAATGGTAGAA |
|  |  | Reverse | TGTGAAGCGCCTAATCTCTTTC |
|  | cyp3a25 (Mouse) | Forward | AAGGCCATTACCATATCTGAGGA |
|  |  | Reverse | TCTCGTCTCAAGTTTCTCACCA |
|  | Nfkb1 (Mouse) | Forward | ATGGCAGACGATGATCCCTAC |
|  |  | Reverse | CGGAATCGAAATCCCCTCTGTT |
|  | Nfkb2 (Mouse) | Forward | TGGCATCCCCGAATATGATGA |
|  |  | Reverse | TGACAGTAGGATAGGTCTTCCG |
|  | Rela (Mouse) | Forward | TGCGATTCCGCTATAAATGCG |
|  |  | Reverse | ACAAGTTCATGTGGATGAGGC |
|  | Relb (Mouse) | Forward | GTTCCAGTGACCTCTCTTCCC |
|  |  | Reverse | CCAAAGCCGTTCTCCTTAATGTA |
|  | Tnf-a (Mouse) | Forward | CAGGCGGTGCCTATGTCTC |
|  |  | Reverse | CGATCACCCCGAAGTTCAGTAG |
|  | Ccl2 (Mouse) | Forward | TAAAAACCTGGATCGGAACCAAA |
|  |  | Reverse | GCATTAGCTTCAGATTTACGGGT |
|  | Ccl3 (Mouse) | Forward | TGTACCATGACACTCTGCAAC |
|  |  | Reverse | CAACGATGAATTGGCGTGGAA |
|  | Ccl4 (Mouse) | Forward | TTCCTGCTGTTTCTCTTACACCT |
|  |  | Reverse | CTGTCTGCCTCTTTTGGTCAG |
|  | Ccl5 (Mouse) | Forward | TTTGCCTACCTCTCCCTCG |
|  |  | Reverse | CGACTGCAAGATTGGAGCACT |
|  | COL1A1（Mouse） | Forward | GCTCCTCTTAGGGGCCACT |
|  |  | Reverse | ATTGGGGACCCTTAGGCCAT |
|  | COL1A2（Mouse） | Forward | TCGTGCCTAGCAACATGCC |
|  |  | Reverse | TTTGTCAGAATACTGAGCAGCAA |
|  | COL3A1（Mouse） | Forward | CTGTAACATGGAAACTGGGGAAA |
|  |  | Reverse | CCATAGCTGAACTGAAAACCACC |
|  | COL5A2（Mouse） | Forward | TTGGAAACCTTCTCCATGTCAGA |
|  |  | Reverse | TCCCCAGTGGGTGTTATAGGA |
|  | COL4A1（Mouse） | Forward | CCTGGCACAAAAGGGACGA |
|  |  | Reverse | ACGTGGCCGAGAATTTCACC |
|  | MMP15（Mouse） | Forward | ATGAAGAGACGAAAACGTGGATG |
|  |  | Reverse | TGGAAGACCAATGGTGTGACC |
|  | MMP7（Mouse） | Forward | CTTACCTCGGATCGTAGTGGA |
|  |  | Reverse | CCCCAACTAACCCTCTTGAAGT |
|  | MMP2（Mouse） | Forward | ACCTGAACACTTTCTATGGCTG |
|  |  | Reverse | CTTCCGCATGGTCTCGATG |
|  | MMP13（Mouse） | Forward | TGTTTGCAGAGCACTACTTGAA |
|  |  | Reverse | CAGTCACCTCTAAGCCAAAGAAA |
|  | Tgf-beta（Mouse） | Forward | CCACCTGCAAGACCATCGAC |
|  |  | Reverse | CTGGCGAGCCTTAGTTTGGAC |
|  | Bmp2（Mouse） | Forward | GGGACCCGCTGTCTTCTAGT |
|  |  | Reverse | TCAACTCAAATTCGCTGAGGAC |
|  | Tgfbr（Mouse） | Forward | TCTGCATTGCACTTATGCTGA |
|  |  | Reverse | AAAGGGCGATCTAGTGATGGA |
|  | fbn1（Mouse） | Forward | TGTGGGGATGGATTCTGCTC |
|  |  | Reverse | AGTGCCGATGTACCCTTTCTG |
|  | acta2（Mouse） | Forward | CCCAGACATCAGGGAGTAATGG |
|  |  | Reverse | TCTATCGGATACTTCAGCGTCA |
|  | vim（Mouse） | Forward | CGTCCACACGCACCTACAG |
|  |  | Reverse | GGGGGATGAGGAATAGAGGCT |
|  | CTSS（Mouse） | Forward | CCATTGGGATCTCTGGAAGAAAA |
|  |  | Reverse | TCATGCCCACTTGGTAGGTAT |
|  | CTSK（Mouse） | Forward | CTCGGCGTTTAATTTGGGAGA |
|  |  | Reverse | TCGAGAGGGAGGTATTCTGAGT |
|  | CTSD（Mouse） | Forward | GCTTCCGGTCTTTGACAACCT |
|  |  | Reverse | CACCAAGCATTAGTTCTCCTCC |
|  | CTSB（Mouse） | Forward | CAGGCTGGACGCAACTTCTAC |
|  |  | Reverse | TCACCGAACGCAACCCTTC |
|  | ADAMTS2（Mouse） | Forward | GAGACGTGGCAGACTTACCTA |
|  |  | Reverse | CCAGAGGTTCGATAAAGAACTCC |
|  | ADAMTSL2（Mouse） | Forward | CTTCAACTCCCGTGTGTATGAC |
|  |  | Reverse | GCAAACCCCTCTCAGGTCG |
|  | ANXA2（Mouse） | Forward | ATGTCTACTGTCCACGAAATCCT |
|  |  | Reverse | TGACTGACCCGTAGGCACTT |
|  | LOXL1（Mouse） | Forward | GAGTGCTATTGCGCTTCCC |
|  |  | Reverse | GGTTGCCGAAGTCACAGGT |
|  | LOX（Mouse） | Forward | CAGCCACATAGATCGCATGGT |
|  |  | Reverse | GCCGTATCCAGGTCGGTTC |
|  | CD86 (Mouse) | Forward | TCAATGGGACTGCATATCTGCC |
|  |  | Reverse | GCCAAAATACTACCAGCTCACT |
|  | iNOS (Mouse) | Forward | GTTCTCAGCCCAACAATACAAGA |
|  |  | Reverse | GTGGACGGGTCGATGTCAC |
|  | IL-6 (Mouse) | Forward | CTGCAAGAGACTTCCATCCAG |
|  |  | Reverse | AGTGGTATAGACAGGTCTGTTGG |
|  | Trem2(Mouse) | Forward | CTGGAACCGTCACCATCACTC |
|  |  | Reverse | CGAAACTCGATGACTCCTCGG |
|  | C1qa (Mouse) | Forward | TTCGGCAGAACCCAATGACG |
|  |  | Reverse | TGGTATGGACTCTCCTGGTTG |
|  | Apoe (Mouse) | Forward | CTCCCAAGTCACACAAGAACTG |
|  |  | Reverse | CCAGCTCCTTTTTGTAAGCCTTT |
|  | Ms4a7 (Mouse) | Forward | CTCCAATGTAGCAAGCTCTGTT |
|  |  | Reverse | CTTCCAATGGTGGGATTTCAAGT |
|  | Ccr2 (Mouse) | Forward | ATCCACGGCATACTATCAACATC |
|  |  | Reverse | TCGTAGTCATACGGTGTGGTG |
|  | Mmp12 (Mouse) | Forward | GGGCTGCTCCCATGAATGAC |
|  |  | Reverse | CCAGAGTTGAGTTGTCCAGTTG |
|  | H2-Ab1 (Mouse) | Forward | AGCCCCATCACTGTGGAGT |
|  |  | Reverse | GATGCCGCTCAACATCTTGC |
|  | H2-Aa (Mouse) | Forward | GACCACGTAGGCACCTATGG |
|  |  | Reverse | CTACAGCTATGTTTTGCAGTCCA |
|  | IL-1B (Mouse) | Forward | GAAATGCCACCTTTTGACAGTG |
|  |  | Reverse | TGGATGCTCTCATCAGGACAG |
|  | CXCL10 (Mouse) | Forward | CCAAGTGCTGCCGTCATTTTC |
|  |  | Reverse | GGCTCGCAGGGATGATTTCAA |
|  | DDX17 (Human) | Forward | GATGTTTGTCCTAAACCCGTGT |
|  |  | Reverse | CCAACGGAAATCCCTGGCA |
|  | CYP2C19 (Human) | Forward | GGAAAACGGATTTGTGTGGGA |
|  |  | Reverse | GGTCCTTTGGGTCAATCAGAGA |
|  | CYP2C9 (Human) | Forward | GCCTGAAACCCATAGTGGTG |
|  |  | Reverse | GGGGCTGCTCAAAATCTTGATG |
|  | CYP2C8 (Human) | Forward | CATTACTGACTTCCGTGCTACAT |
|  |  | Reverse | CTCCTGCACAAATTCGTTTTCC |
| siRNAs | si-CTCF (Human) | sense | GGAGCCUGCCGUAGAAAUUTT |
|  |  | antisense | AAUUUCUACGGCAGGUCCTC |
|  | si-DDX5 (Human) | sense | ACGAAGUAUAUAGAAAAGCGU |
|  |  | antisense | GCUUUUCUAUAUACUUCGUUC |
|  | si-CYP2C19 #1 (Human) | sense | GGAAAGAGAUGGAAGGAGATT |
|  |  | antisense | UCUCCUUCCAUCUCUUUCCTT |
|  | si-CYP2C19 #2 (Human) | sense | UCAAAAUGGAGAAGGAAAATT |
|  |  | antisense | UUUUCCUUCUCCAUUUUGATT |
|  | si-CYP2C19 #3 (Human) | sense | GGAUGAAGGUGGAAAUUUUTT |
|  |  | antisense | AAAAUUUCCACCUUCAUCCTT |
| CHIP | cyp2c29 Probe 1 | Forward | TCACAGAGAAAGGAGCTTCAGT |
|  |  | Reverse | TCCCCCTTGATCACTAATTGAGAA |
|  | cyp2c29 Probe 2 | Forward | GCTTTTGATTGTGACTGTGCCT |
|  |  | Reverse | AGTCTCTTAACTGTGGGCTCC |
|  | cyp2c29 Probe 3 | Forward | GCTAAGCAAGCCAGTAAAGAGC |
|  |  | Reverse | AGAACTCAAGCAGGTCAGGGA |

**Supplementary Table 6.** Lipid metabolism related genes of DDX17 between Flox-MCD and CKO-MCD group

| gene_name | flox1 | flox2 | flox3 | CKO1 | CKO2 | CKO3 |
| --- | --- | --- | --- | --- | --- | --- |
| Cyp2c29 | 114.6634 | 80.52771 | 104.5173 | 498.6457 | 472.4293 | 384.7088 |
| Cyp2c37 | 116.4518 | 86.24975 | 162.755 | 593.2465 | 558.3901 | 927.1049 |
| Cyp3a41a | 1.795986 | 1.556982 | 1.710403 | 8.262103 | 17.75242 | 50.84918 |
| Cyp2c70 | 102.2241 | 92.81801 | 103.968 | 233.6852 | 282.8404 | 341.4381 |
| Cyp2c68 | 156.7223 | 162.193 | 214.1482 | 515.5674 | 452.6922 | 448.2853 |
| Cyp2c50 | 110.27 | 68.90547 | 83.82116 | 213.2056 | 300.5364 | 301.4164 |
| Cyp2j9 | 2.494575 | 1.181751 | 1.075065 | 6.046829 | 5.528135 | 8.47898 |
| Cyp2b9 | 140.3408 | 71.13443 | 84.07333 | 299.3599 | 307.708 | 260.4991 |
| Cyp2c40 | 12.15295 | 9.834562 | 19.43571 | 33.0483 | 47.38537 | 46.36637 |
| Cyp2c39 | 3.623195 | 2.980598 | 5.605898 | 11.0087 | 16.19421 | 10.89673 |
| Cyp3a25 | 285.2557 | 131.387 | 175.7333 | 636.0927 | 453.66 | 628.1469 |
| Cyp3a41b | 2.741479 | 0.891619 | 0.962177 | 7.821035 | 87.86627 | 37.78751 |
| Cyp2c38 | 12.81969 | 5.223185 | 6.966699 | 21.7112 | 26.64786 | 21.70594 |
| Cyp2c69 | 0.338128 | 0.9506 | 1.483931 | 4.692237 | 14.82602 | 64.84903 |
| Akr1c14 | 80.2931 | 99.39726 | 98.85265 | 276.6205 | 201.4646 | 130.0829 |
| Cyp2b13 | 8.647215 | 1.121708 | 8.404983 | 78.76349 | 108.0514 | 34.67564 |
| Cyp3a59 | 27.72026 | 8.614516 | 13.31166 | 54.80669 | 55.64377 | 30.50466 |
| Cyp3a11 | 565.7323 | 643.6592 | 523.0775 | 1268.08 | 1370.61 | 751.6 |
| Cyp2c54 | 7.258476 | 7.074084 | 3.693817 | 15.40717 | 35.43366 | 114.4953 |
| Cyp46a1 | 0.281391 | 0.207953 | 0.097448 | 0.907476 | 0.770693 | 0.671543 |
| Cyp2d40 | 23.75255 | 8.243855 | 19.87531 | 35.02784 | 35.69858 | 58.32933 |
| Cyp2r1 | 5.607992 | 2.908684 | 4.089088 | 9.734129 | 7.524406 | 8.633994 |
| Cyp2c67 | 79.41279 | 42.41013 | 67.94606 | 129.9427 | 106.4341 | 118.8722 |
| Cyp26a1 | 17.85976 | 13.84935 | 6.287001 | 31.50355 | 19.41707 | 34.78417 |
| Cyp2a12 | 120.7926 | 109.1147 | 109.1303 | 159.086 | 236.5045 | 152.0643 |
| Fmo3 | 28.65824 | 32.2279 | 92.70858 | 108.8071 | 254.2501 | 599.8731 |
| Cyp2a22 | 36.87728 | 11.87439 | 26.81508 | 123.5118 | 132.1172 | 34.75548 |
| Cyp2j8 | 0.17992 | 0 | 0.042369 | 0.541553 | 0.4296 | 0.663589 |
| Msmo1 | 26.20324 | 24.39017 | 32.41698 | 31.70942 | 45.19615 | 56.15515 |
| Rdh10 | 3.884404 | 3.753913 | 3.464912 | 7.086014 | 8.150677 | 7.203509 |
| Ugt2a3 | 48.05981 | 50.09636 | 62.3375 | 91.83431 | 94.77931 | 105.0267 |
| Ugt1a9 | 5.341406 | 5.882244 | 2.457187 | 17.51961 | 22.12642 | 4.184426 |
| Hsd17b6 | 38.0682 | 59.4572 | 59.18143 | 70.37096 | 100.6708 | 122.6524 |
| Ugt2b35 | 37.40031 | 37.99101 | 43.31219 | 121.2763 | 103.4764 | 35.84445 |
| Ugt2b36 | 191.1804 | 190.4527 | 180.4263 | 338.6835 | 285.344 | 232.441 |
| Ugt1a5 | 7.483235 | 5.179243 | 6.775548 | 6.026653 | 14.65407 | 18.5518 |
| Hsd11b1 | 63.39259 | 54.89304 | 76.90197 | 149.088 | 158.6071 | 137.7132 |

**Supplementary Table 7.** Inflammation related genes of DDX17 between Flox-MCD and CKO-MCD group

| gene_name | flox1 | flox2 | flox3 | CKO1 | CKO2 | CKO3 |
| --- | --- | --- | --- | --- | --- | --- |
| Gpnmb | 27.93892064 | 71.8072197 | 65.70474544 | 2.837292744 | 1.844762502 | 2.485003788 |
| Apoa4 | 255.5818181 | 485.5037215 | 384.3389399 | 29.35665484 | 35.07243625 | 44.33517645 |
| Mmp12 | 11.88596791 | 24.83330814 | 29.61899944 | 1.648680168 | 1.08603366 | 0.896546057 |
| Adam8 | 2.463484345 | 8.922058771 | 5.815062288 | 0.088273859 | 0.168061018 | 0.432663196 |
| C3ar1 | 2.616124729 | 5.935867596 | 6.011892812 | 0.624956076 | 0.3017681 | 0.481668207 |
| Cyba | 18.11848194 | 30.51566624 | 30.52057216 | 6.192156964 | 6.439292682 | 8.240505724 |
| Cebpb | 110.4073407 | 117.151454 | 123.0133858 | 53.16006427 | 50.2714755 | 56.74953162 |
| C5ar1 | 1.630117306 | 3.314385214 | 3.14080115 | 0.273094492 | 0.303294553 | 0.598624043 |
| Rab7b | 0.858946849 | 2.012688349 | 1.648664002 | 0.265623374 | 0.294997243 | 0.216986581 |
| Cybb | 6.285750936 | 11.06281602 | 11.04105286 | 2.880077965 | 2.348600642 | 3.650834096 |
| Cd74 | 50.24040455 | 68.09304448 | 82.8267414 | 28.28981328 | 25.75189444 | 17.7423823 |
| Cyp1b1 | 0.806356019 | 1.712338204 | 1.349207537 | 0.353572514 | 0.228003218 | 0.208704306 |
| Ly9 | 1.661683988 | 2.257746829 | 2.731416555 | 0.61901143 | 0.432120604 | 0.667481431 |
| Src | 2.477042888 | 3.512555027 | 2.901933148 | 0.936660169 | 1.010519258 | 1.224245803 |
| Cd34 | 0.880675136 | 2.228702205 | 1.265429584 | 0.377081433 | 0.302947852 | 0.367021838 |
| F2r | 12.36244635 | 22.58609966 | 17.53265775 | 7.819403706 | 6.892768749 | 5.293431875 |
| Tlr7 | 1.759390275 | 2.318572051 | 2.322692591 | 0.587456448 | 0.700158175 | 0.852098472 |
| Anxa1 | 7.572866385 | 14.29419623 | 15.08216902 | 5.090765464 | 3.063472686 | 4.026720713 |
| Tlr8 | 1.395850153 | 3.19644547 | 1.911509725 | 0.636090839 | 0.561523449 | 0.484978522 |
| Cd300c2 | 6.18755502 | 7.134392689 | 9.446059656 | 2.660618631 | 2.496332227 | 3.147752003 |
| Zc3hav1 | 7.721268108 | 9.228534921 | 7.646598026 | 4.675070735 | 4.464575237 | 3.920202038 |
| Irf7 | 7.132379935 | 21.24925164 | 6.752336949 | 3.60901556 | 3.646870682 | 2.816596154 |
| Tlr2 | 2.36477216 | 2.302776426 | 3.188240179 | 1.14491589 | 0.818948962 | 0.9542994 |
| Gprc5b | 0.669815389 | 3.032154748 | 1.605018408 | 0.366337878 | 0.462966362 | 0.130023233 |
| Cd276 | 1.353502142 | 2.472326577 | 1.803833894 | 0.735493687 | 0.461685394 | 0.585147994 |
| Hk1 | 0.561168237 | 0.936728713 | 0.593170117 | 0.212508187 | 0.26646102 | 0.188156765 |
| Ptafr | 0.912531343 | 1.4456987 | 1.232984901 | 0.450276778 | 0.28575456 | 0.165523115 |
| Clu | 586.5342529 | 723.0714628 | 710.0727812 | 211.1140142 | 232.4755985 | 436.4472174 |
| Fcgr3 | 8.430703492 | 16.20410888 | 15.49896113 | 2.560523054 | 2.768843996 | 7.051163941 |
| Cd84 | 2.862655892 | 4.929380787 | 5.270437547 | 1.948969557 | 1.315267714 | 2.122298514 |

**Supplementary Table 8.** Liver fibrosis related genes of DDX17 between Flox-MCD and CKO-MCD group

| gene_name | flox1 | flox2 | flox3 | CKO1 | CKO2 | CKO3 |
| --- | --- | --- | --- | --- | --- | --- |
| Col1a1 | 5.620675928 | 12.70638091 | 7.486795647 | 0.668380357 | 0.831368126 | 0.560556933 |
| Lgals3 | 22.34292793 | 55.18359546 | 51.51194013 | 3.494154372 | 3.600874648 | 5.292136878 |
| Col3a1 | 8.502606221 | 14.44666121 | 10.78800958 | 2.907703313 | 2.097689482 | 1.131183292 |
| Ctss | 80.77035917 | 138.303373 | 172.6570574 | 27.90872877 | 24.82470384 | 39.4541044 |
| Col1a2 | 2.570206445 | 5.314707672 | 4.547160204 | 1.01198 | 1.155253818 | 0.979714379 |
| Ccdc80 | 5.107465173 | 8.524510357 | 6.774823478 | 2.343324199 | 2.299848285 | 2.18135621 |
| Anxa2 | 23.78427777 | 20.80090354 | 21.08512293 | 8.135924839 | 7.695086543 | 3.800756573 |
| Loxl1 | 1.666723976 | 3.85937972 | 2.020772447 | 0.585409562 | 0.275819887 | 0.18259253 |
| Lox | 0.904911624 | 2.076967095 | 0.918982061 | 0.243192254 | 0.256581307 | 0.097344721 |
| Cyp1b1 | 0.806356019 | 1.712338204 | 1.349207537 | 0.353572514 | 0.228003218 | 0.208704306 |
| Col5a2 | 1.248015692 | 1.984567026 | 1.576344484 | 0.634211726 | 0.270902235 | 0.397530457 |
| Loxl2 | 1.40926801 | 2.743307568 | 1.647487475 | 0.530231104 | 0.336495159 | 0.594025355 |
| Adamts2 | 2.016399179 | 2.545649946 | 2.223244296 | 1.01489004 | 1.036673301 | 1.046047621 |
| Col4a1 | 13.55821666 | 18.68902783 | 16.99403776 | 8.243448482 | 7.484164225 | 4.37328353 |
| Serpinh1 | 9.309236659 | 15.49637513 | 8.10285434 | 3.543260375 | 4.945451709 | 2.43683421 |
| Efemp2 | 2.897092676 | 4.348577613 | 2.733779498 | 1.082288187 | 1.422743142 | 1.086200299 |
| Thbs2 | 1.173151438 | 1.42656172 | 1.106889489 | 0.60133044 | 0.389566544 | 0.362960123 |
| Fbln2 | 0.529557976 | 1.774680262 | 0.864892315 | 0.302997696 | 0.244730613 | 0.168011881 |
| Mmp13 | 0.386737155 | 0.806503004 | 0.623128204 | 0.065642803 | 0.02430063 | 0 |
| Mfap4 | 0.934161174 | 2.544196535 | 0.799946381 | 0.213014651 | 0.236570803 | 0.069604298 |
| Ddr1 | 1.891851003 | 6.29991523 | 4.307503483 | 1.666177681 | 1.197337944 | 0.840675284 |
| App | 20.4845661 | 29.36488827 | 32.81988898 | 13.17279214 | 16.40383387 | 9.274799443 |
| Adamtsl2 | 4.090966426 | 8.953016891 | 6.147358918 | 3.24159484 | 2.584245658 | 2.527298696 |
| Ctgf | 9.356393988 | 27.23650873 | 21.00332246 | 6.766669344 | 8.96800261 | 4.091472478 |
| Tgfb1 | 2.136601875 | 3.837656231 | 3.621137685 | 1.542814169 | 1.455767737 | 0.981721544 |
| Fbln5 | 1.976032507 | 2.191152912 | 2.595494769 | 1.320226739 | 1.033875587 | 0.813013649 |
| Col14a1 | 12.55266488 | 10.78142432 | 12.61516279 | 6.963818695 | 7.863580545 | 6.094757951 |
| Eln | 1.384046573 | 4.239908657 | 1.131257013 | 0.816141553 | 0.6898222 | 0.355181328 |
| Crispld2 | 0.432802351 | 2.542598756 | 1.475164537 | 0.416282827 | 0.339939206 | 0.266046655 |
| Sh3pxd2b | 0.762124045 | 1.418887236 | 1.431819432 | 0.343372234 | 0.307751245 | 0.654498337 |

**Additional files**

**Additional files 1**. **Flow diagram for participants included in the study.**


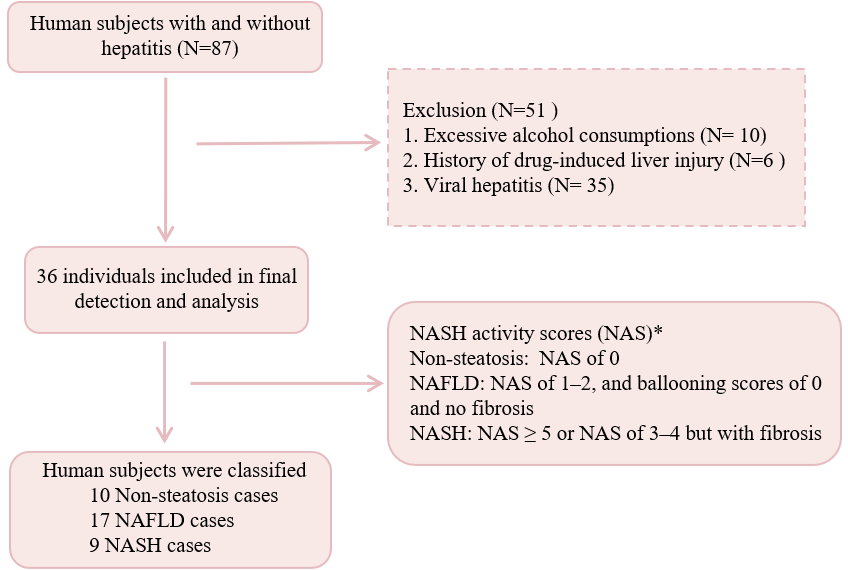


* Non-steatosis, NAFLD and NASH were independently diagnosed by two pathologists according to the scoring system of standard histological criteria established by the NASH Clinical Research Network.
